# Effects of Empagliflozin on Fluid Overload, Weight, and Blood Pressure in CKD

**DOI:** 10.1681/ASN.0000000000000271

**Published:** 2023-12-12

**Authors:** Kaitlin J. Mayne, Natalie Staplin, David F. Keane, Christoph Wanner, Susanne Brenner, Vladimir Cejka, Johannes Stegbauer, Parminder K. Judge, David Preiss, Jonathan Emberson, Daniele Trinca, Rejive Dayanandan, Ryonfa Lee, John Nolan, Akiko Omata, Jennifer B. Green, David Z.I. Cherney, Lai Seong Hooi, Roberto Pontremoli, Katherine R. Tuttle, Jennifer S. Lees, Patrick B. Mark, Simon J. Davies, Sibylle J. Hauske, Dominik Steubl, Martina Brückmann, Martin J. Landray, Colin Baigent, Richard Haynes, William G. Herrington

**Affiliations:** 1 Medical Research Council Population Health Research Unit, Clinical Trial Service Unit and Epidemiological Studies Unit (CTSU), Nuffield Department of Population Health, University of Oxford, Oxford, United Kingdom; 2 School of Cardiovascular and Metabolic Health, College of Medical and Veterinary Life Sciences, University of Glasgow, Glasgow, United Kingdom; 3 CÚRAM SFI Research Centre for Medical Devices, HRB-Clinical Research Facility Galway, National University of Ireland Galway, Galway, Ireland; 4 Würzburg University Clinic, Würzburg, Germany; 5 Department of Nephrology, Medical Faculty, University Hospital Düsseldorf, Heinrich-Heine-University Düsseldorf, Düsseldorf, Germany; 6 CARID, Cardiovascular Research Institute Düsseldorf, Medical Faculty and University Hospital Düsseldorf, Heinrich-Heine-University Düsseldorf, Dusseldorf, Germany; 7 Oxford Kidney Unit, Oxford University Hospitals NHS Foundation Trust, Oxford, United Kingdom; 8 Leeds Teaching Hospitals NHS Trust, Leeds, United Kingdom; 9 Duke Clinical Research Institute, Durham, North Carolina; 10 University of Toronto, Toronto, Canada; 11 Department of Medicine and Haemodialysis Unit, Sultanah Aminah Hospital, Johor Bahru, Malaysia; 12 Università degli Studi and IRCCS Ospedale Policlinico San Martino di Genova, Genoa, Italy; 13 Providence Inland Northwest Health, University of Washington, Spokane, Washington; 14 School of Medicine, Keele University, Newcastle, United Kingdom; 15 Boehringer Ingelheim International GmbH, Ingelheim upon Rhein, Germany; 16 The Fifth Department of Medicine, University Medical Center Mannheim, Mannheim, Germany; 17 University of Heidelberg, Mannheim, Germany; 18 Department of Nephrology, Hospital Rechts der Isar, Technical University of Munich, Munich, Germany; 19 The First Department of Medicine, University Medical Center Mannheim, Mannheim, Germany

**Keywords:** BP, cardiovascular, CKD, clinical trial, diabetes, heart failure

## Abstract

**Significance Statement:**

SGLT2 inhibitors reduce risk of kidney progression, AKI, and cardiovascular disease, but the mechanisms of benefit are incompletely understood. Bioimpedance spectroscopy can estimate body water and fat mass. One quarter of the EMPA-KIDNEY bioimpedance substudy CKD population had clinically significant levels of bioimpedance-derived “Fluid Overload” at recruitment. Empagliflozin induced a prompt and sustained reduction in “Fluid Overload,” irrespective of sex, diabetes, and baseline N-terminal pro B-type natriuretic peptide or eGFR. No significant effect on bioimpedance-derived fat mass was observed. The effects of SGLT2 inhibitors on body water may be one of the contributing mechanisms by which they mediate effects on cardiovascular risk.

**Background:**

CKD is associated with fluid excess that can be estimated by bioimpedance spectroscopy. We aimed to assess effects of sodium glucose co-transporter 2 inhibition on bioimpedance-derived “Fluid Overload” and adiposity in a CKD population.

**Methods:**

EMPA-KIDNEY was a double-blind placebo-controlled trial of empagliflozin 10 mg once daily in patients with CKD at risk of progression. In a substudy, bioimpedance measurements were added to the main trial procedures at randomization and at 2- and 18-month follow-up visits. The substudy's primary outcome was the study-average difference in absolute “Fluid Overload” (an estimate of excess extracellular water) analyzed using a mixed model repeated measures approach.

**Results:**

The 660 substudy participants were broadly representative of the 6609-participant trial population. Substudy mean baseline absolute “Fluid Overload” was 0.4±1.7 L. Compared with placebo, the overall mean absolute “Fluid Overload” difference among those allocated empagliflozin was −0.24 L (95% confidence interval [CI], −0.38 to −0.11), with similar sized differences at 2 and 18 months, and in prespecified subgroups. Total body water differences comprised between-group differences in extracellular water of −0.49 L (95% CI, −0.69 to −0.30, including the −0.24 L “Fluid Overload” difference) and a −0.30 L (95% CI, −0.57 to −0.03) difference in intracellular water. There was no significant effect of empagliflozin on bioimpedance-derived adipose tissue mass (−0.28 kg [95% CI, −1.41 to 0.85]). The between-group difference in weight was −0.7 kg (95% CI, −1.3 to −0.1).

**Conclusions:**

In a broad range of patients with CKD, empagliflozin resulted in a sustained reduction in a bioimpedance-derived estimate of fluid overload, with no statistically significant effect on fat mass.

**Trial Registration:**

Clinicaltrials.gov: NCT03594110; EuDRACT: 2017-002971-24 (https://eudract.ema.europa.eu/).

## Visual Abstract

**Figure:**
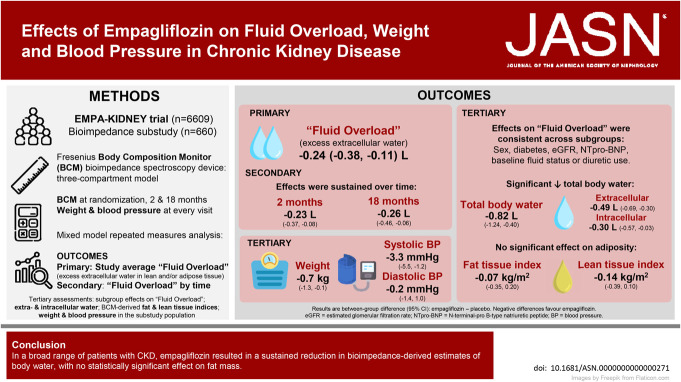

## Introduction

**Figure 1:**
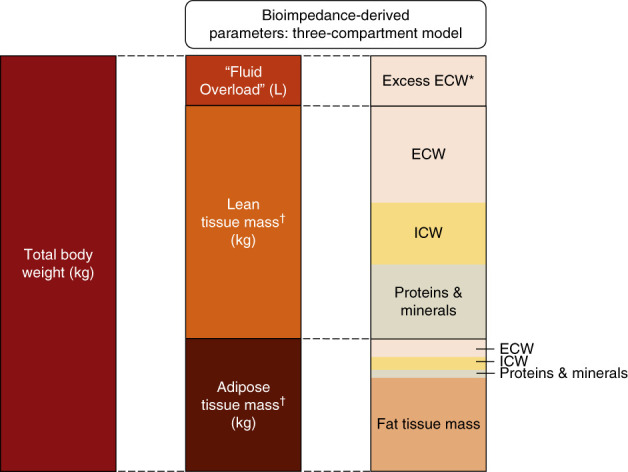
**Relationship of the derived “Fluid Overload” parameter to body weight and tissue mass.** Based on the three-compartment model described by Chamney *et al.*^9^ *Excess ECW accumulates both in tissues and in the blood (although blood volume is not specifically conceptualized in the three-compartment model), so changes in Fluid Overload could reflect changes in excess ECW that might be residing in adipose tissue, lean tissue, or both. ^†^Refers to normally hydrated lean and adipose tissue mass. Total body water (TBW) is the sum of ECW and ICW, although TBW is not conceptualized in the three-compartment model. The figure is not to scale because compartment proportions vary between individuals and “Fluid Overload” is usually smaller than depicted (and can be a negative value in fluid depletion). The mean baseline values in the EMPA-KIDNEY substudy were total body weight 88.8 kg, “Fluid Overload” 0.4 L, lean tissue mass 38.8 kg, and adipose tissue mass 49.6 kg. In the EMPA-KIDNEY substudy, mean total ECW at baseline was 18.7 L and ICW 20.4 L. ECW, extracellular water; ICW, intracellular water.

Patients with CKD are at increased risk of cardiovascular disease,^1,2^ key features of which are structural heart disease, heart failure, and sudden death.^3–5^ These risks increase progressively as eGFR decreases,^6^ with risk of death from cardiovascular disease exceeding risk of progression to kidney failure for many people with CKD. Fluid excess is common in CKD, especially when heart failure coexists,^7^ and can be quantified using bioimpedance spectroscopy.^8^ Bioimpedance can estimate a number of fluid- and adiposity-related parameters, including the excess constituent of total body extracellular water (ECW) over and above what is considered normohydration. We refer to this parameter as “Fluid Overload” (refer to Figure 1 and the Supplemental Methods for more details about bioimpedance spectroscopy and a glossary of fluid-related terms).^9^ “Fluid Overload” can be used to guide dialysis prescription,^10^ and epidemiologically there are positive associations between bioimpedance-measured “Fluid Overload” with cardiovascular outcomes and mortality in patients on dialysis, with nondialysis CKD, or with heart failure.^8^

The double-blind international multicenter Study of Heart and Kidney Protection With Empagliflozin (EMPA-KIDNEY) demonstrated that, compared with matching placebo, empagliflozin 10 mg once daily reduced the risk of kidney disease progression or cardiovascular death by 28% (95% confidence interval [CI], 18% to 36%) in 6609 patients with CKD at risk of progression.^11^ A meta-analysis of large placebo-controlled trials extended these findings and showed that in people with CKD, heart failure, or type 2 diabetes at high cardiovascular risk, sodium glucose co-transporter 2 (SGLT2) inhibitors safely reduce the risk of kidney disease progression by about two fifths and AKI by about a quarter, with consistent effects irrespective of diabetes status.^12^ SGLT2 inhibitors also reduce the risk of cardiovascular outcomes, particularly hospitalization for heart failure.^12^ These cardiovascular benefits are particularly large in patients with preexisting heart failure,^12,13^ but smaller numbers of cardiovascular events in patients with CKD without diabetes and at low levels of eGFR mean effects are less certain in these populations.^11,12^ The amount of glycosuria induced by SGLT2 inhibition falls with decreasing eGFR and with ambient normoglycemia,^14^ so it is reasonable to hypothesize that other effects of SGLT2 inhibitors could also be attenuated in such patients.^11,15^ To address uncertainty about the effects of SGLT2 inhibitors on fluid status and adiposity in CKD, we embedded a bioimpedance-based substudy within the EMPA-KIDNEY trial.^11^ The primary aim was to assess the effects of empagliflozin 10 mg once daily versus placebo on fluid status using the bioimpedance-derived parameter of absolute “Fluid Overload” (*i.e.*, estimated excess ECW). We also aimed to assess effects on this “Fluid Overload” parameter over time and in different types of patients with CKD. In this report, we also put the substudy findings regarding empagliflozin's effects on bioimpedance-derived fluid and adiposity parameters in the context of its potentially related effects on weight, BP, glycated hemoglobin and hematocrit (as observed in the full trial cohort).

## Methods

### Substudy Design and Population

The full methods of the EMPA-KIDNEY trial and the main results have been reported elsewhere (ClinicalTrials.gov number, NCT03594110; EudraCT number, 2017-002971-24).^11,16^ In brief, patients with CKD at risk of progression were identified based on historical and screening local laboratory measurements of an eGFR ≥20 but <45 ml/min per 1.73 m^2^, or an eGFR ≥45 but <90 ml/min per 1.73 m^2^ with a urinary albumin-creatinine ratio (uACR) ≥200 mg/g. This report details the results of an optional substudy conducted in a subset of sites in the United Kingdom and Germany which added bioimpedance measurements at the randomization, 2- and 18-month follow-up visits to the trial's main protocol-specified procedures (substudy protocol supplement available in the Supplemental Materials). All participants provided written informed consent. Regulatory authorities, as well as ethics committees at each center, approved the trial and the substudy which adhere to the Declaration of Helsinki.

### Bioimpedance Measurements

Bioimpedance spectroscopy is a tool used in the clinical care of patients requiring dialysis to monitor fluid status.^17^ We used the Fresenius Medical Care Body Composition Monitor (BCM) bioimpedance spectroscopy device because it has been extensively validated for fluid status assessment in kidney failure populations and used in randomized controlled trials.^18–20^ The device passes low-level electrical current at frequencies of 5–1000 kHz (with results extrapolated from zero to infinity kHz) between electrodes attached to patients' hands and feet.^8^ All substudy bioimpedance measurements were performed by trained local research coordinators. Body fluid and adiposity indices were then derived centrally using age, sex, a paired weight measurement, and height data combined with bioimpedance measurements of electrical resistance, and a validated three-compartment model formula using proprietary coefficients.^9,21^

The primary outcome was based on the bioimpedance-derived estimate of excess ECW which we refer to as absolute “Fluid Overload” (sometimes referred to as “overhydration”). It is reported in liters and can have positive or negative values (Figure 1). Its reference range estimated from the 10th and 90th centiles of a reference general population distribution is −1.1 L to +1.1 L.^22^ “Fluid Overload” can be indexed to ECW volume and referred to as percentage relative “Fluid Overload.” An absolute value of +1.1 L approximately corresponds to relative “Fluid Overload” of +7%.^23^ Values above this threshold have been consistently associated with an increased risk of death and cardiovascular events,^8^ and we refer to it as moderate “Fluid Overload” (>7%, ≤15%) or severe “Fluid Overload” (>15%).^8,23,24^ Bioimpedance measurements were also used to derive estimates of extracellular and intracellular water (ICW) volume, lean tissue index (LTI), and fat tissue index (FTI) (see Supplemental Methods for more details).

Local research coordinators were trained to repeat measurements when the BCM device's automated quality score (the Q value) was below 80 (out of 100). Visual inspection of reactance versus resistance plots (known as Cole–Cole plots) were additionally used to assess data quality.^25^ It was not always possible to obtain a Q value ≥80, so any measurement with a Q value <80 had its Cole–Cole plot assessed independently by two researchers to determine data quality and inclusion in the primary assessment using prespecified rules blind to treatment allocation (see prespecified Data Analysis Plan provided in the Supplemental Materials for details). Absolute “Fluid Overload” values lower than −5 L were consistently associated with low-quality bioimpedance measurement and were considered invalid.

### Outcomes

The substudy's prespecified primary outcome was the effect of empagliflozin versus placebo on mean absolute “Fluid Overload” averaged over time, with effects on relative “Fluid Overload” provided for completeness. It was estimated that at least 382 participants would provide >90% power (at a two-sided *P* value of 0.05) to detect at least a 0.3-L difference in absolute “Fluid Overload” between treatment groups. The key secondary outcome was the effect of empagliflozin versus placebo on time to the first event of a cardiovascular composite defined as death from heart failure, heart failure hospitalization, or development of new moderate or severe “Fluid Overload” (in participants without this level of “Fluid Overload” at baseline). The other secondary outcomes were the effects of empagliflozin versus placebo on “Fluid Overload” at the different measurement time points. Tertiary assessments are detailed in the Supplemental Methods and include analyses of the effects of empagliflozin versus placebo on all ECW (of which “Fluid Overload” is a constituent) and ICW. In addition, the effects of empagliflozin versus placebo on total body water (the sum of all ECW and ICW) were assessed as a *post hoc* analysis to contextualize effects on “Fluid Overload.”

In order for inferences from the bioimpedance substudy to be put in the context of findings from all the available EMPA-KIDNEY data, additional analyses included assessments of the effects of empagliflozin versus placebo on weight, body mass index (BMI), waist-to-hip ratio, glycated hemoglobin, hematocrit, and BP (systolic and diastolic) in the full trial cohort. Analyses emphasized results of study-average effects including all available measurements from routine trial visit time points (with effects at 2 and 18 months also presented). The full cohort results are emphasized because of greater statistical power and wider generalizability than the substudy. Substudy results were compared with results from the full cohort using standard statistical tests of heterogeneity. Analyses of weight and systolic BP also considered results for the same subgroups as the substudy (plus self-reported race, to explore effects by race in the full trial cohort because the substudy took place in the United Kingdom and Germany only). Prespecified sensitivity analysis for the primary outcome included three analyses assessing any effect of data quality assessments. Analyses of effects of empagliflozin on diuretic use were included *post hoc*.

### Statistical Analysis

Substudy analyses followed the intention-to-treat principle and required a consenting participant to have provided at least one valid bioimpedance measurement. The primary outcome was prespecified to be assessed using a mixed model repeated measures (MMRM) approach adjusted for age, sex, previous diabetes, eGFR, and uACR in the categories used in the minimized randomization algorithm.^11^ The MMRM model also included fixed categorical effects of time (to avoid assuming a linear association between treatment allocation and “Fluid Overload” over time), treatment allocation, and treatment-by-time interaction, and continuous effects of baseline (randomization) measurements and baseline-by-time interaction. The within-person error correlations were assumed to be unstructured. Analyses of the full trial cohort were additionally adjusted for region.^11^ Effects at each follow-up time point were estimated and used to derive study-average effects (with weights proportional to the amount of time between visits). All between-group differences are reported as empagliflozin minus placebo. To assess effect modification, subgroup-specific treatment effects were estimated by fitting interaction terms in the MMRM models. The null hypothesis was that the treatment effect is the same across all subgroups. This was tested by calculating a heterogeneity or trend statistic from subgroup-specific means and standard errors, without correction for multiplicity of testing.

The key secondary outcome and its components were analyzed using an adjusted Cox proportional hazards regression using the same covariates in the minimization algorithm (age, sex, previous diabetes, eGFR, and uACR) and included the complete substudy population of 660 participants (*i.e.*, it included participants without a valid follow-up bioimpedance measurement who were excluded from MMRM analyses but were at risk of clinical outcomes). Tertiary analyses used the same MMRM approach as described for the primary outcome and assessed effects on ECW, ICW, LTI, FTI, body weight, and BMI. Waist and hip circumference measurements were obtained at a single follow-up time point (18 months) and were therefore analyzed by analysis of covariance, adjusted for the baseline value and minimization variables. Handling of missing data is outlined in the Supplemental Methods. *P* values for hypothesis testing for outcomes are limited to the primary outcome. *P* values for testing for any evidence of effect modification between subgroups, and between treatment effect and effects by time are provided. The prespecified Data Analysis Plan is provided in the Supplemental Materials. Analyses were performed using R Studio version 4.2.2 (RStudio: Integrated Development for R. RStudio, PBC, Boston, MA) and SAS version 9.4 (SAS Institute, Cary, NC).

## Results

### Substudy Baseline Characteristics and Adherence

Between May 22, 2019, and April 14, 2021, 668 participants consented to join the substudy. One was excluded because of a metal knee implant and no useable bioimpedance measurement at baseline excluded a further seven, leaving 660 included in analyses (Supplemental Figure 1, Supplemental Material). MMRM analyses excluded 40 consenting participants with no valid follow-up bioimpedance measurement (empagliflozin versus placebo: 21 versus 19, respectively; three due to death before first follow-up measurement, 28 with no follow-up measurement performed [*e.g.*, due to coronavirus disease 2019 precluding visits], and nine due to low data quality). This left a total of 620 participants for whom 1047 valid follow-up bioimpedance measurements were available for MMRM analyses.

In the substudy, the mean age was 64 (15) years and 205 participants (31%) were female (Table 1). At recruitment, 136 (21%) reported a diagnosis of heart failure and 256 (39%) had diabetes. The mean (SD) eGFR was 36.0 (12.4) ml/min per 1.73 m^2^ and median (Q1–Q3) N-terminal pro–B-type natriuretic peptide (NT-proBNP) was 211 (93–581) ng/L. The mean body weight was 88.8 (19.8) kg and mean BMI was 30.3 (6.2) kg/m^2^. The mean absolute “Fluid Overload” at baseline was 0.4 (1.7) L with 126 (19%) and 30 (5%) participants with evidence of moderate and severe “Fluid Overload,” respectively (Table 1). Severity of “Fluid Overload” mirrored established markers of fluid excess: heart failure was twice as common in those with severe “Fluid Overload” compared with the normohydrated group and NT-proBNP was five-fold higher (Supplemental Table 2). In addition, participants with “Fluid Overload” were more likely to be older, be male, to have previous diabetes, and have a lower eGFR (Supplemental Table 2). The substudy cohort characteristics were broadly representative of the full trial cohort,^11^ although were less racially diverse due to being conducted only in the United Kingdom and Germany (Supplemental Table 3).

**Table 1:** Bioimpedance substudy cohort: baseline characteristics

Baseline Characteristic	Empagliflozin (*n*=332)	Placebo (*n*=328)
Demographics		
Age (yr)	65.2 (14.2)	64.1 (14.9)
Female sex	102 (30.7)	103 (31.4)
White race	321 (96.7)	315 (96.0)
Previous disease		
Diabetes	135 (40.7)	121 (36.9)
Heart failure	62 (18.7)	74 (22.6)
Clinical measurements		
Weight (kg)	89.8 (20.2)	87.9 (19.3)
Body mass index (kg/m^2^)	30.5 (6.2)	30.1 (6.3)
Waist-to-hip ratio	1.0 (0.1)	1.0 (0.1)
Systolic BP (mm Hg)	137.0 (18.8)	137.5 (18.9)
Diastolic BP (mm Hg)	77.8 (12.2)	78.6 (11.9)
Bioimpedance measurements^a^		
Absolute “Fluid Overload” (L)	0.45 (1.68)	0.32 (1.68)
Relative “Fluid Overload” (%)		
Mean (SD)	1.9 (8.7)	1.3 (8.3)
Moderate “Fluid Overload”	70 (21.1)	56 (17.1)
Severe “Fluid Overload”	14 (4.2)	16 (4.9)
Extracellular water (L)	19.0 (3.8)	18.4 (3.7)
Intracellular water (L)	20.7 (4.5)	20.1 (4.6)
Lean tissue index (kg/m^2^)	13.3 (3.1)	12.9 (3.0)
Fat tissue index (kg/m^2^)	12.6 (5.4)	12.5 (5.1)
Laboratory measurements		
eGFR (ml/min per 1.73 m^2^)		
Mean (SD)	36.1 (13.4)	35.8 (11.4)
Distribution		
<30	123 (37.0)	118 (36.0)
≥30 <45	148 (44.6)	154 (47.0)
≥45	61 (18.4)	56 (17.1)
Urinary albumin-creatinine ratio (mg/g)	203 (26–958)	205 (29–865)
HbA1c (mmol/mol)	43.9 (11.3)	43.5 (10.9)
NT-proBNP (ng/L)	197 (90–596)	225 (95–550)
Medications		
RAS inhibitor	304 (91.6)	288 (87.8)
Any diuretic therapy	180 (54.2)	173 (52.7)

Data are presented as mean (SD) or median (Q1–Q3) for continuous variables and *n* (%) for categorical variables. HbA1c, glycated hemoglobin; NT-proBNP, N-terminal pro–brain-type natriuretic peptide; RAS, renin-angiotensin system.

a Bioimpedance measurements are presented for 644 of 660 participants with a baseline measurement (missing for 16/660) irrespective of validity for inclusion in the primary analysis.

Substudy adherence to study treatment was consistent with adherence in the full-trial population.^11^ At 12 months of follow-up (the approximate midpoint of the trial), of substudy participants who remained alive, 282 of 318 (88.7%) in the empagliflozin group and 292 of 320 (91.3%) in the placebo group reported taking at least 80% of their allocated study treatment.

### Effects on Bioimpedance-Derived Parameters

**Figure 2:**
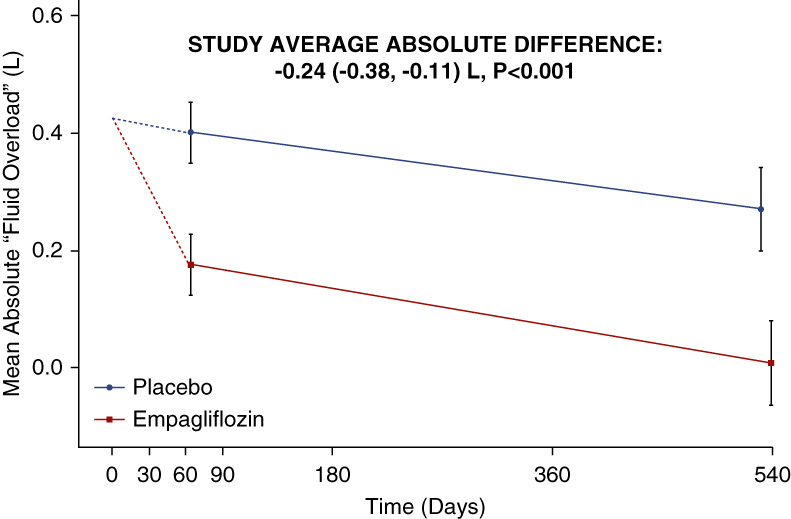
**Effects of empagliflozin on mean bioimpedance-derived absolute “Fluid Overload” by time.** The value at time 0 is the average across all randomized participants. Follow-up means (and their CIs) are derived from a repeated measures mixed model adjusted for baseline values, age, sex, diabetes, eGFR, and uACR. Follow-up values are plotted at the median follow-up day in each time window. There was no significant interaction between treatment allocation and time (*P* = 0.11). The study average is the between-group difference (empagliflozin minus placebo) in weighted averages of both time points (see Supplemental Methods). Analyses excluded 40 consenting participants with no valid follow-up measurements. Median (Q1–Q3) follow-up since randomization for empagliflozin versus placebo groups at the 2-month visit: 64 (57–74) versus 64 (57–75) days, Wilcoxon rank sum *P* = 0.871; and at the 18-month visit: 540 (519–555) versus 532 (505–554) days, *P* = 0.026. CI, confidence interval; uACR, urinary albumin-creatinine ratio.

**Figure 3:**
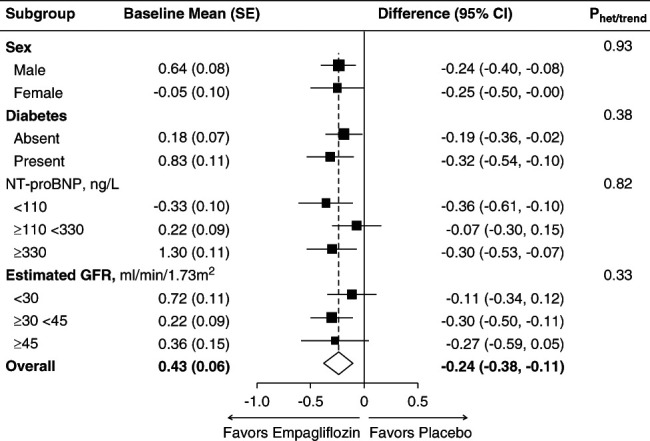
**Effects of empagliflozin on mean bioimpedance-derived absolute “Fluid Overload” (in liters) by prespecified substudy subgroups.** Study-average differences are adjusted for baseline values of the dependent variable (in continuous form) and for any differences in key baseline characteristics (categories of age, sex, diabetes, eGFR, and urinary albumin-creatinine ratio) between treatment groups and weighted in proportion to the amount of time between follow-up visits (see Supplemental Methods). Analysis excluded 40 consenting participants with no valid follow-up measurements (3 deaths before first follow-up measurement, 28 with no measurement performed, and 9 excluded because of inadequate data quality). Further details are available in Supplemental Table 5. NT-proBNP, N-terminal pro B-type natriuretic peptide; SE, standard error.

The primary assessment found that the study-average mean absolute “Fluid Overload” was 0.24 L lower in those allocated to the empagliflozin group compared with the placebo group (absolute difference in means −0.24 L, 95% CI, −0.38 to −0.11), with similar differences at 2 months (−0.23 L, 95% CI, −0.37 to −0.08) and 18 months (−0.26 L, 95% CI, −0.46 to −0.06) (Figure 2, Table 2). Findings were robust in sensitivity analyses assessing the effect of data quality assessments (Supplemental Table 4). The effect of empagliflozin on the primary outcome was similar in subgroups by sex, diabetes status, and across the spectrum of NT-proBNP and eGFR studied (*P* values for heterogeneity or trend >0.3, Figure 3 and Supplemental Table 5). Neither was there any evidence of heterogeneity in *post hoc* exploratory subgroups divided by baseline fluid status (fluid depletion, low and high normohydration, and moderate and severe “Fluid Overload”; *P* = 0.71), diuretic use (*P* = 0.07), or uACR (*P* = 0.33, Supplemental Figure 2).

**Table 2:** Effects of empagliflozin on bioimpedance-derived parameters

Bioimpedance-Derived Parameter	Empagliflozin (*n*=311)		Placebo (*n*=309)		Absolute Difference		95% CI	*P* Value for Primary Outcome
	Adjusted^a^ Mean	SE	Adjusted^a^ Mean	SE				
Primary assessments								
Absolute “Fluid Overload”, L								
Study average	0.10	0.05	0.34	0.05	−0.24	(−0.38 to −0.11)	<0.001
Relative “Fluid Overload”, %								
Study average	0.14	0.25	1.33	0.25	−1.19	(−1.90, to −0.48)	0.001
Secondary assessments								
Absolute “Fluid Overload”, L								
Randomization	0.50	0.09	0.35	0.09				
2-mo follow-up	0.18	0.05	0.40	0.05	−0.23	(−0.37 to −0.08)	
18-mo follow-up	0.01	0.07	0.27	0.07	−0.26	(−0.46 to −0.06)	
Relative “Fluid Overload”, %								
Randomization	2.24	0.47	1.39	0.45				
2-mo follow-up	0.52	0.27	1.65	0.27	−1.12	(−1.88 to −0.37)	
18-mo follow-up	−0.36	0.38	0.92	0.37	−1.28	(−2.32 to −0.23)	
Tertiary assessments								
Extracellular water, L								
Study average	18.16	0.07	18.66	0.07	−0.49	(−0.69 to −0.30)	
Intracellular water, L								
Study average	20.10	0.10	20.40	0.10	−0.30	(−0.57 to −0.03)	
Lean tissue index (LTI), kg/m^2^								
Study average	12.90	0.09	13.05	0.09	−0.14	(−0.39 to 0.10)	
Fat tissue index (FTI), kg/m^2^								
Study average	12.34	0.10	12.42	0.10	−0.07	(−0.35 to 0.20)	

CI, confidence interval; SE, standard error.

a Mean effects are adjusted for baseline values of the dependent variable (in continuous form) and for any differences in key baseline characteristics (categories of age, sex, diabetes, eGFR, and urinary albumin-creatinine ratio) between treatment groups with study averages weighted in proportion to the amount of time between follow-up visits (see Supplemental Methods). Analysis excluded 40 consenting participants with no valid follow-up measurements (3 deaths before first follow-up measurement, 28 with no measurement performed, and 9 excluded because of inadequate data quality). Effects on “Fluid Overload” did not vary by time: *P* value for interaction with time=0.11 and 0.39 for absolute and relative “Fluid Overload”, respectively.

There was no significant difference in the composite outcome between treatment groups (empagliflozin 35/332 [11%] versus placebo 38/328 [12%], hazard ratio (HR) 0.91, 95% CI, 0.57 to 1.45, *P* = 0.69) with consistent effects for its components (Table 3). The number of outcomes was low, limiting statistical power: development of new moderate “Fluid Overload” occurred in 7.8% of substudy participants allocated empagliflozin versus 10.1% allocated placebo, and development of new severe “Fluid Overload” occurred in 2.6% versus 1.3% of empagliflozin and placebo groups, respectively. The tertiary outcome of regression of moderate or severe “Fluid Overload” did not differ significantly between the empagliflozin and placebo groups (54.8% versus 48.6%; Table 3). Heart failure events were also infrequent; there were no deaths due to heart failure in the substudy population. In the full trial cohort, hospitalization for heart failure occurred in 2.7% and 3.2% of participants allocated empagliflozin and placebo, respectively (HR 0.80, 95% CI, 0.60 to 1.06), and findings from the substudy cohort considered in isolation were consistent (empagliflozin 3.3% versus placebo 4.9%; HR 0.67, 95% CI, 0.31 to 1.46; Table 3).

**Table 3:** Effects of empagliflozin on cardiovascular composite outcome (bioimpedance substudy cohort)

Outcome	Empagliflozin		Placebo		Hazard Ratio	95% CI	*P* Value
	No. of Participants / Total	%	No. of Participants / Total	%			
Key secondary assessment							
Death from heart failure, hospitalization for heart failure, development of new moderate or severe “Fluid Overload”	35/332	10.5	38/328	11.6	0.91	(0.57 to 1.45)	0.69
Death from heart failure	0/332	0.0	0/328	0.0	—	—	
Hospitalization for heart failure	11/332	3.3	16/328	4.9	0.67	(0.31 to 1.46)	
Development of new moderate “Fluid Overload”^a^	18/232	7.8	25/247	10.1	0.68	(0.37 to 1.26)	
Development of new severe “Fluid Overload”^b^	8/302	2.6	4/303	1.3	1.96	(0.57 to 6.71)	
Tertiary assessment							
Regression of “Fluid Overload”^c^	46/84	54.8	35/72	48.6	1.33	(0.82 to 2.18)	

All analyses use a time-to-first-event approach. Cox proportional hazards models include adjustment for the covariates used in the minimization algorithm: age, sex, diabetes status, eGFR, and urinary albumin-creatinine ratio. Results were consistent in *post hoc* sensitivity analyses additionally adjusted for use of any diuretic or loop diuretics at baseline (hazard ratios [95% CIs] 0.89 [0.56 to 1.42] and 0.92 [0.58 to 1.47]; respectively). CI, confidence interval.

a Requires randomization value of relative “Fluid Overload” ≤7% and follow-up value >7%, ≤15%.

b Requires randomization value of relative “Fluid Overload” ≤15% and follow-up value >15%.

c Requires randomization value consistent with moderate or severe relative “Fluid Overload” and regression to any lower hydration category at any follow-up (limited to first event). All 660 participants were included in the composite outcome analysis because all participants were at risk of the clinical components of the composite. In the full-trial cohort, there were 88 (2.7%) first hospitalizations for heart failure in the empagliflozin group versus 107 (3.2%) in the placebo group: hazard ratio 0.80, 95% CI, 0.60 to 1.06.

Bioimpedance estimated that the study-average absolute difference in total body water was −0.82 L (−1.24 to −0.40). This consisted of differences in ECW of −0.49 L (95% CI, −0.69 to −0.30) (of which the −0.24 L between-group difference in “Fluid Overload” is a constituent) and ICW of −0.30 L (95% CI, −0.57 to −0.03). There were no significant between-group differences in bioimpedance-derived fat or lean tissue index or related tissue mass parameters (lean, fat, and adipose tissue mass in kg; Table 2, Supplemental Tables 6 and 7). In the bioimpedance substudy population, the study-average between-group difference in weight was −0.7 kg (−1.3 to −0.1). Supplemental Figure 3 shows the change in weight (relative to baseline) with the change in different biompedance indices at the 2-month follow-up visit.

### Effects on Anthropometry, BP, and Relevant Laboratory Values in the Full Trial Cohort

**Figure 4:**
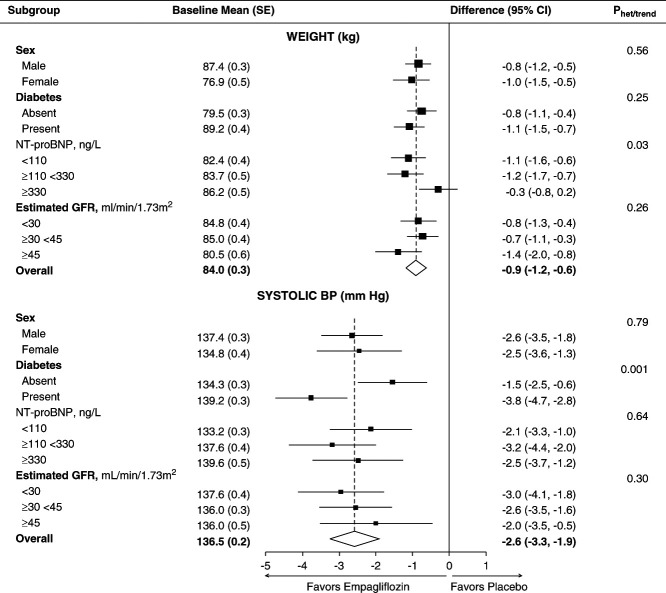
**Full trial cohort: effects of empagliflozin on weight and systolic BP overall and by key bioimpedance substudy prespecified subgroups.** Study-average differences are adjusted for baseline values of the dependent variable (in continuous form) and for any differences in key baseline characteristics (categories of age, sex, diabetes, eGFR, urinary albumin-creatinine ratio, and region) between treatment groups and weighted in proportion to the amount of time between follow-up visits (see Supplemental Methods). Each analysis includes all individuals with at least one follow-up measurement of the outcome variable with mean imputation of missing baseline measurements. For comparison, between-group differences in the substudy cohort were −0.7 (95% CI −1.3 to −0.1) kg and −3.3 (−5.5 to −1.2) mm Hg for weight and systolic BP, respectively.

In the full trial cohort, the between-group difference in weight was −0.9 kg (95% CI, −1.2 to −0.6) (Figure 4, Supplemental Table 8) and the effect of empagliflozin on weight did not vary significantly over time (interaction *P* value by time = 0.47, Supplemental Table 8). In the full cohort, there was no evidence of heterogeneity of the effect of empagliflozin on weight in subgroups by sex, baseline eGFR, or diabetes (Figure 4, or in *post hoc* analyses by race: Supplemental Figure 4). The waist-to-hip ratio at 18 months was also not significantly different between the empagliflozin versus placebo groups (Supplemental Table 9). The study-average difference in glycated hemoglobin (HbA1c) in the full cohort was −0.4 mmol/mol (95% CI, −0.8 to −0.0), with a −0.9 mmol/mol (95% CI, −1.6 to −0.1) difference in HbA1c in participants with diabetes at randomization and no significant difference in participants without diabetes (0.0 mmol/mol, 95% CI, −0.2 to 0.2; Supplemental Table 10). The full trial cohort average between-group difference in hematocrit at 18 months postrandomization was 2.3% (95% CI, 1.9 to 2.7).

The study-average between-group differences in systolic and diastolic BP were −2.6 (95% CI, −3.3 to −1.9) and −0.5 mm Hg (95% CI, −0.9 to −0.1), respectively. In the full trial cohort, there was no evidence of heterogeneity of the effect of empagliflozin on systolic BP when subdivided by sex, baseline eGFR, NT-proBNP (Figure 4), or race (Supplemental Figure 4), but there was some evidence to suggest a larger systolic BP difference in patients with diabetes (Figure 4). Effects on anthropometry, HbA1c, hematocrit, and BP in the substudy were approximately consistent with the full trial cohort results (Supplemental Tables 8–11).

### Effects on Diuretic Use

Among those participants in the full trial cohort who were not taking a loop diuretic at randomization, 159 of 2453 (6.5%) in the empagliflozin group compared with 212 of 2409 (8.8%) in the placebo group started such medication during follow-up, representing a 26% lower likelihood of a new loop diuretic prescription among the empagliflozin group (risk ratio 0.74, 95% CI, 0.60 to 0.90).

## Discussion

In the EMPA-KIDNEY substudy of 660 patients with CKD, empagliflozin resulted in a sustained reduction in bioimpedance-derived “Fluid Overload” for at least 18 months, irrespective of diabetes status or level of kidney function. Using the three-compartment model, we observed a −0.24 L between-group difference in “Fluid Overload” but no significant differences in normally hydrated lean or adipose tissue compartments. Fluid volume differences consisted of approximately 0.8 L less total body water of which approximately 0.5 L was ECW and approximately 0.3 L ICW (with the approximately 0.5 L total ECW difference including the −0.24 L between-group difference in excess ECW referred to as “Fluid Overload”). These data raise a hypothesis that an important determinant of the substudy −0.7 kg weight difference was due to effects on fluid status. Along with other mechanisms,^26^ this effect may contribute to the cardiovascular benefits of SGLT2 inhibitors.

Osmotic diuretic and natriuretic actions are considered potentially important contributing mechanisms to the cardiovascular benefits of SGLT2 inhibitors, but their effects on fluid status in CKD—where effects may be hypothesized to be attenuated by decreased kidney function—have not previously been quantified in randomized trials.^15,26–28^ In patients with type 2 diabetes without kidney disease, mechanistic trials have reported plasma volume reductions by SGLT2 inhibitors^29^ and raised a hypothesis that SGLT2 inhibitors reduce interstitial volume more than plasma volume.^28^ Previously collected bioimpedance data in patients taking SGLT2 inhibitors are limited to mainly nonrandomized studies.^30–33^ To the best of our knowledge, the 16-week DECREASE trial provides the only peer reviewed published randomized evidence on the effects of SGLT2 inhibitors on bioimpedance parameters to date. It found that, in 66 participants with type 2 diabetes—CKD status not reported—dapagliflozin reduced extracellular fluid by approximately 1 L and systolic BP by approximately 4 mm Hg at 10 days versus placebo.^34^ EMPA-KIDNEY now substantially extends these previous findings by studying longer term effects (over 18 months) in a much larger number of participants in a placebo-controlled trial.

Before the results of this substudy, attenuation of diuretic effects at low levels of kidney function was considered plausible as SGLT2 inhibitors have little effect on glycemia at lower eGFR due to attenuated levels of glycosuria.^11,14,35–37^ Despite this, we found consistent effects on “Fluid Overload” across the eGFR-based subgroups. Similarly, effects did not vary by baseline fluid status, diuretic use, or albuminuria. These findings are analogous to results from large randomized trials in heart failure populations that included a large proportion of patients with CKD and low eGFR and demonstrated consistent effects of SGLT2 inhibitors on cardiovascular death or hospitalization for heart failure irrespective of sex, diabetes, eGFR, or NT-proBNP at baseline.^13^

It is also relevant that the effect of empagliflozin on fluid loss in EMPA-KIDNEY was achieved safely. Although estimates of ECW reduction reflected loss of ECW that is not considered to be in excess by the three-compartment model, there was no increased risk of participant reports of symptomatic dehydration in the full trial or substudy cohorts (Supplemental Table 12) nor any increased risk of AKI.^11^

We also report assessments of the effects of empagliflozin on anthropometry, BP, HbA1c, and hematocrit for the full trial and substudy cohorts, with the full trial data providing better statistical power to assess for any effect modification between subgroups of participant. The effects of empagliflozin on weight and HbA1c in EMPA-KIDNEY are generally consistent with results from other CKD trials. Evaluation of the Effects of Canagliflozin on Renal and Cardiovascular Outcomes in Participants With Diabetic Nephropathy (CREDENCE) studied 4401 participants with type 2 diabetes and a mean eGFR of 56 ml/min per 1.73 m^2^_._ Compared with placebo, the mean weight was 0.80 kg (95% CI, 0.69 to 0.92) lower in the canagliflozin group, and there was a relatively modest difference in HbA1c (−0.25%, 95% CI, −0.31 to −0.20).^38^ The Study to Evaluate the Effect of Dapagliflozin on Renal Outcomes and Cardiovascular Mortality in Patients With Chronic Kidney Disease trial studied 4304 participants with a mean eGFR of 43 ml/min per 1.73 m^2^ and included 2996 participants with diabetes.^39^ The between-group difference in HbA1c in those with diabetes was −1.1 mmol/mol (95% CI, −2.1 to 0.0).^40^ The overall between-group difference in systolic BP in EMPA-KIDNEY of −2.6 mm Hg (95% CI, −3.3 to −1.9) was also similar to the other large CKD trials: CREDENCE difference −3.3 mm Hg (95% CI, −3.9 to −2.7)^38^ and DAPA-CKD difference −2.9 mm Hg (95% CI, −3.6 to −2.3).^41,42^ In EMPA-KIDNEY, there were somewhat larger antihypertensive effects in participants with diabetes (heterogeneity *P* = 0.001). This pattern was not observed in bioimpedance-derived “Fluid Overload” analyses, raising the hypothesis that SGLT2 inhibition may have additional antihypertensive effects that are more prominent in patients with diabetes, and which are distinct from their diuretic effects (possibly through effects on vascular stiffness or endothelial function).^43–45^ The lack of measured effect of empagliflozin on adiposity is consistent with its modest effects on glycated hemoglobin observed in CKD populations.

### Study Limitations

EMPA-KIDNEY demonstrated the clear benefits of SGLT2 inhibition on kidney disease progression in a wide range of patients with CKD at risk of progression, including about a one-third reduction in the risk of needing to start kidney replacement therapy.^11^ This large EMPA-KIDNEY substudy benefits from its sample size, long duration, systematic measurements, and randomized double-blind design. These help ensure between-group differences are unbiased and reliable. The BCM device has some technical limitations. For example, BCM parameters are derived, not direct measurements and are based on formulae normalized to healthy reference populations. Estimations may also be less accurate at extremes of “Fluid Overload” (although extremes of levels were uncommon in the substudy population). Furthermore, imprecision in fat mass estimates mean the lack of statistical effect on fat mass does not exclude some effect. The BCM device also does not reliably assess subtypes of adiposity (*e.g.*, visceral versus peripheral). Follow-up was affected by coronavirus disease 2019 restrictions resulting in some missed bioimpedance measurements, and the prespecified key secondary composite analysis was underpowered because of lower cardiovascular risk in the trial population than was predicted during its design. Nevertheless, this substudy collected sufficient data to provide reliable and clear results for the primary and other continuously measured outcomes. Owing to the regions contributing to the substudy, Asian, Black, Mixed, and Other races were underrepresented, but effects on weight, HbA1c, and BP for the full trial cohort were broadly similar to the substudy results across the studied races, suggesting our conclusions are likely to be generalizable. Finally, the use of other diuretics was determined by local doctors and not controlled by the protocol. We observed more new use of loop diuretics among those allocated to placebo, so the presented estimates of effects on fluid parameters, weight, and BP may be slight underestimates of the full effect of empagliflozin.

In summary, the EMPA-KIDNEY bioimpedance substudy found that fluid excess is common in a broad population of patients with CKD at risk of progression and that empagliflozin resulted in sustained reductions in “Fluid Overload,” weight, and BP in patients with CKD with and without diabetes, even in patients with low levels of kidney function.

## Supplementary Material

## Data Availability

The complete deidentified patient dataset used for presented analyses will be available in due course and the application system to apply to use data will open 6 months after publication. Departmental policy details can be found here https://www.ndph.ox.ac.uk/data-access. In adherence with the Boehringer Ingelheim Policy on Transparency and Publication of Clinical Study Data, scientific and medical researchers can request access to clinical study data after publication of the primary manuscript and secondary analyses in peer-reviewed journals and regulatory and reimbursement activities are completed, normally within 1 year after the marketing application has been granted by major regulatory authorities. Researchers should use the https://vivli.org/link to request access to study data and visit https://www.mystudywindow.com/msw/datasharing for further information.

## Supplemental Material

Members of the EMPA-KIDNEY Collaborative Group.

Supplemental Methods.

Substudy Protocol Supplement.

Substudy Data Analysis Plan.

Supplemental Table 1. Bioimpedance substudy cohort: additional baseline characteristics.

Supplemental Table 2. Bioimpedance substudy cohort: baseline characteristics by categories of baseline bioimpedance-derived “Fluid Overload.”

Supplemental Table 3. Baseline characteristics for the substudy, substudy region, and the full trial cohorts.

Supplemental Table 4. Sensitivity analyses for the effects of empagliflozin on mean bioimpedance-derived absolute “Fluid Overload” in L.

Supplemental Table 5. Bioimpedance substudy cohort: unadjusted baseline means and adjusted study averages for “Fluid Overload” for each subgroup by treatment group (additional data to accompany Figure 3).

Supplemental Table 6. Effects of empagliflozin on tertiary (and *post hoc*) bioimpedance-derived parameters assessments by time.

Supplemental Table 7. Effects of empagliflozin on other bioimpedance-derived adiposity parameters.

Supplemental Table 8. Effects of empagliflozin on weight and body mass index (bioimpedance substudy and full trial cohorts).

Supplemental Table 9. Effects of empagliflozin on waist and hip measurements (bioimpedance substudy and full trial cohorts).

Supplemental Table 10. Effects of empagliflozin on glycated hemoglobin and hematocrit (bioimpedance substudy and full trial cohorts).

Supplemental Table 11. Effects of empagliflozin on BP (bioimpedance substudy and full trial cohorts).

Supplemental Table 12. Bioimpedance substudy cohort: effects of empagliflozin on dehydration by categories of baseline bioimpedance-derived “Fluid Overload.”

Supplemental Figure 1. Bioimpedance substudy cohort CONSORT flow chart.

Supplemental Figure 2. Effects of empagliflozin on mean absolute “Fluid Overload” (bioimpedance substudy cohort: *post hoc* subgroups).

Supplemental Figure 3. Correlation between change in weight (relative to baseline) with change in different bioimpedance indices at the 2 month follow-up visit, by treatment allocation.

Supplemental Figure 4. Effects of empagliflozin on weight, body mass index, systolic BP, glycated hemoglobin, and hematocrit by race (full trial cohort).
