# Effects of Empagliflozin on Fluid Overload, Weight and Blood Pressure in Chronic Kidney Disease

**DOI:** 10.1681/ASN.0000000000000271

**Published:** 2023-12-12

**Authors:** The EMPA-KIDNEY Bioimpedance Substudy Group, Kaitlin J Mayne, Natalie Staplin, David F Keane, Christoph Wanner, Susanne Brenner, Vladimir Cejka, Johannes Stegbauer, Parminder K Judge, David Preiss, Jonathan Emberson, Daniele Trinca, Rejive Dayanandan, Ryonfa Lee, John Nolan, Akiko Omata, Jennifer B Green, David ZI Cherney, Lai Seong Hooi, Roberto Pontremoli, Katherine R Tuttle, Jennifer S Lees, Patrick B Mark, Simon J Davies, Sibylle J Hauske, Dominik Steubl, Martina Brückmann, Martin J Landray, Colin Baigent, Richard Haynes, William G Herrington, the EMPA-KIDNEY Collaborative Group, Executive Committee, Colin Baigent, Martin J. Landray, Christoph Wanner, William G. Herrington, Richard Haynes, Jennifer B. Green, Sibylle J. Hauske, Martina Brueckmann, Mark Hopley, Previous members, Maximillian von-Eynatten, Jyothis George, Steering Committee, Executive Committee members plus National Representatives, Susanne Brenner, Alfred K. Cheung, David Preiss, Zhi-Hong Liu, Jing Li, Laiseong Hooi, Wen Liu, Takashi Kadowaki, Masaomi Nangaku, Adeera Levin, David Cherney, Roberto Pontremoli, Aldo P. Maggioni, plus statistician members, Natalie Staplin, Jonathan Emberson, Stefan Hantel, plus other expert members, Shinya Goto, Rajat Deo, Katherine R. Tuttle, Non-voting members, Michael Hill, Parminder Judge, Kaitlin J. Mayne, Sarah Y.A. Ng, Xavier Rossello, Emily Sammons, Doreen Zhu, Independent Data Monitoring Committee, Peter Sandercock, Rudolf Bilous, Charles Herzog, Paul Whelton, Janet Wittes, Derrick Bennett, List of Collaborators, by Site, Administration, Patricia Achiri, Chrissie Ambrose, Cristina Badin, Jill Barton, Richard Brown, Andy Burke, Sebastian Butler, Rejive Dayanandan, Pia Donaldson, Robert Dykas, Lucy Fletcher, Kate Frederick, Hannah Kingston, Mo Gray, Emily Harding, Akiko Hashimoto, Lyn Howie, Susan Hurley, Ryonfa Lee, Nik Luker, Kevin Murphy, Mariko Nakahara, John Nolan, Michelle Nunn, Sorcha Mulligan, Akiko Omata, Sandra Pickworth, YanRu Qiao, Shraddha Shah, Karen Taylor, Alison Timadjer, Monique Willett, Liz Wincott, Qin Yan, Hui Yu, Clinical, Louise Bowman, Fang Chen, Robert Clarke, Michelle Goonasekera, Richard Haynes, William G. Herrington, Parminder Judge, Waseem Karsan, Marion Mafham, Kaitlin J. Mayne, Sarah Y.A. Ng, David Preiss, Christina Reith, Emily Sammons, Mohammed Zayed, Doreen Zhu, Data Analysis, Ritva Ellison, Rowan Moys, Will Stevens, Kevin Verdel, Karl Wallendszus, Finance, Chris Bowler, Anna Brewer, Andy Measor, IT Validation, Guanguo Cui, Charles Daniels, Angela Field, Bob Goodenough, Ashley Lawson, Youcef Mostefai, Dheeptha Radhakrishnan, Samee Syed, Shuang Xia, Laboratory, Ruth Adewuyi-Dalton, Thomas Arnold, Anne-Marie Beneat, Anoushka Bhatt, Chloe Bird, Andrew Breach, Laura Brown, Mark Caple, Tatyana Chavagnon, Karen Chung, Sarah Clark, Luminita Condurache, Katarzyna Eichstadt, Marta Espino Obrero, Scarlett Forest, Helen French, Nick Goodwin, Andrew Gordon, Joanne Gordon, Cat Guest, Tina Harding, Michael Hill, Michal Hozak, Matthew Lacey, David MacLean, Louise Messinger, Stewart Moffat, Martin Radley, Claire Shenton, Sarah Tipper, Jon Tyler, Lesley Weaving, James Wheeler, Elissa Williams, Tim Williams, Hamish Woodhouse, Monitoring, Angela Chamberlain, Jo Chambers, Joanne Davies, Denise Donaldson, Pati Faria-Shayler, Denise Fleming-Brown, Jennifer Ingell, Carol Knott, Anna Liew, Helen Lochhead, Juliette Meek, Isabel Rodriguez-Bachiller, Andrea Wilson, Patrick Zettergren, Programming, Rach AitSadi, Ian Barton, Alex Baxter, Yonghong Bu, Lukasz Danel, Sonja Grotjahn, Rijo Kurien, Michael Lay, Archie Maskill, Aleksandra Murawska, Rachel Raff, Allen Young, Principal Investigators, Colin Baigent, Richard Haynes, William G. Herrington, Martin J. Landray, David Preiss, Statistics, Jonathan Emberson, Rebecca Sardell, Natalie Staplin, Regional Coordinating Centres, Germany (Universitätsklinikum Würzburg), Christoph Wanner, Susanne Brenner, Vladimir Cejka, Marcela Fajardo-Moser, Christian Hartner, Doris Poehler, Janina Renner, Franziska Scheidemantel, United Kingdom (Clinical Trial Service Unit and Epidemiological Studies Unit [CTSU], University of Oxford), Richard Haynes, David Preiss, William G. Herrington, Parminder Judge, Doreen Zhu, Sarah Y.A. Ng, Kaitlin J. Mayne, Cristina Badin, Jo Chambers, Joanne Davies, Denise Donaldson, Mo Gray, Emily Harding, Jenny Ingell, Yanru Qiao, Shraddha Shah, Andrea Wilson, Patrick Zettergren, Local Clinical Centres, Germany, Universitatsklinikum Wurzburg, Christoph Wanner, Susanne Brenner, Vladimir Cejka, Sharang Ghavampour, Anja Knoppe, Zentrum fuer Nieren-, Hochdruck-und Stoffwechselerkrankungen Hannover, Hans Schmidt-Gurtler, Hubert Dumann, Sybille Merscher, Margret Patecki, Georg Rainer Schlieper, Anke Torp, Bianca Weber, Maja Zietz, Klinikum der Universitat Munchen, Thomas Sitter, Louise Fuessl, Julia Krappe, Jerome Loutan, Volker Vielhauer, Luciano Andriaccio, Magdalena Maurer, ClinPhenomics GmbH Co. KG, Bernhard Winkelmann, Martin Dursch, Linda Seifert, Linda Tenbusch, Universitatsmedizin Mainz, Julia Weinmann-Menke, Simone Boedecker, Wiebke Kaluza-Schilling, Daniel Kraus, Carina Krieger, Margit Schmude, Anne Schreiber, Ewelina Eckrich, Herz-und Diabeteszentrum Nordrhein-Westfalen, Diethelm Tschope, Abdulwahab Arbi, Young Lee-Barkey, Bernd Stratmann, Natalie Prib, Sina Rolfsmeier, Irina Schneider, Universitatsklinikum Dusseldorf, Lars Rump, Johannes Stegbauer, Christine Pötz, Mara Schemmelmann, Claudia Schmidt, Studienzentrum fur Nieren-und Hochdruckerkrankungen Hannover, Hermann Haller, Jessica Kaufeld, Jan Menne, Elisabeth Bahlmann-Kroll, Angela Bergner, UK, Oxford University Hospitals, Richard Haynes, William G Herrington, Doreen Zhu, Madita Gavrila, Kathryn Lafferty, Ria Rabara, Sally Ruse, Maria Weetman, Nottingham City Hospital, Cath Byrne, Mark Jesky, Alison Cowley, Emma McHaffie, Holly Waterfall, Dorset County Hospital, Jo Taylor, Laura Bough, Thomas Phillips, Barbara Winter-Goodwin, Imperial College Healthcare NHS Trust, Andrew Frankel, James Tomlinson, Marlon Alegata, Rashid Almasarwah, Anthoula Apostolidi, Maria Vourvou, Thomas Walters, The Queen Elizabeth Hospital, King’s Lynn, Shiva Ugni, Smita Gunda, Rotimi Oluyombo, Vicki Brindle, Ping Coutts, Tracy Fuller, Evelyn Nadar, University Hospital Aintree, Christopher Wong, Christopher Goldsmith, Sherald Barnes, Ann Bennett, Claire Burston, Samantha Hope, Nicola Hunt, Lini Kurian, UHNM Royal Stoke University Hospital, Richard Fish, Daniela Farrugia, Judy Lee, Emma Sadler, Hannah Turner, Royal Devon and Exeter Hospital, Helen Clarke, Victoria Carnall, Sarah Benyon, Caroline Blake, Stephanie Estcourt, Jane Piper, Daisy Hill Hospital, Neal Morgan, Carolyn Hutchinson, Teresa McKinley, Kent & Canterbury Hospital, Tim Doulton, Michael Delaney, Mahmoud Montasser, Jenny Hansen, David Loader, Angela Moon, Frances Morris, University Hospital of Wales, Cardiff, Donald Fraser, Mohammad Alhadj Ali, Sian Griffin, Farah Latif, Justyna Witczak, Alexa Wonnacott, Lynda Jeffers, Yvette Webley, Ninewells Hospital, Samira Bell, Leanne Cosgrove, Rachel Craik, Shona Murray, Northern General Hospital, Sheffield, Arif Khwaja, Yvonne Jackson, Angeline Mbuyisa, Rachel Sellars, St. James’s University Hospital, Leeds, Andrew Lewington, Richard Baker, Suzannah Dorey, Kay Tobin, Rosalyn Wheatley, Glasgow Clinical Research Facility, Queen Elizabeth University Hospital, Rajan Patel, Patrick Mark, Alastair Rankin, Michael Sullivan, Kirsty Forsyth, Rowan McDougall

**Affiliations:** Germany; United States; United Kingdom; China; Malaysia; Japan; Canada; Italy; 1 Medical Research Council Population Health Research Unit, Clinical Trial Service Unit and Epidemiological Studies Unit (CTSU), Nuffield Department of Population Health, University of Oxford, Oxford, UK; 2 School of Cardiovascular and Metabolic Health, College of Medical and Veterinary Life Sciences, University of Glasgow, Glasgow, UK; 3 CÚRAM SFI Research Centre for Medical Devices, HRB-Clinical Research Facility Galway, National University of Ireland Galway, Ireland; 4 Würzburg University Clinic, Würzburg, Germany; 5 Department of Nephrology, Medical Faculty, University Hospital Düsseldorf, Heinrich-Heine-University Düsseldorf, Düsseldorf, Germany; CARID, Cardiovascular Research Institute Düsseldorf, Medical Faculty and University Hospital Düsseldorf, Heinrich-Heine-University Düsseldorf, Germany; 6 Oxford Kidney Unit, Oxford University Hospitals NHS Foundation Trust, Oxford, UK; 7 Leeds Teaching Hospitals NHS Trust, Leeds, UK; 8 Duke Clinical Research Institute, Durham, North Carolina, USA; 9 University of Toronto, Toronto, Canada; 10 Department of Medicine and Haemodialysis Unit, Sultanah Aminah Hospital, Johor Bahru, Malaysia; 11 Università degli Studi and IRCCS Ospedale Policlinico San Martino di Genova, Genoa, Italy; 12 Providence Inland Northwest Health, University of Washington, Spokane, Washington, USA; 13 School of Medicine, Keele University, UK; 14 Boehringer Ingelheim International GmbH, Ingelheim / Rhein, Germany; 15 The Fifth Department of Medicine, University Medical Center Mannheim, Mannheim, Germany; 16 University of Heidelberg, Mannheim, Germany; and the Department of Nephrology, Hospital Rechts der Isar, Technical University of Munich, Munich, Germany; 17 The First Department of Medicine, Faculty of Medicine, Mannheim, Germany

**Keywords:** Bioimpedance, SGLT2 inhibitor, overhydration, body composition, adiposity, anthropometry

## Abstract

**Background:**

Chronic kidney disease (CKD) is associated with fluid excess which can be estimated by bioimpedance spectroscopy. We aimed to assess effects of sodium glucose co-transporter 2 inhibition on bioimpedance-derived “Fluid Overload” and adiposity in a CKD population.

**Methods:**

EMPA-KIDNEY was a 6609-participant double-blind placebo-controlled trial of empagliflozin 10 mg once daily in patients with CKD at risk of progression. In a 660-participant substudy, bioimpedance measurements were added to the main trial procedures at randomization, 2- and 18-month follow-up visits. The substudy’s primary outcome was the study-average difference in absolute “Fluid Overload” (an estimate of excess extracellular water) analyzed using a mixed-model repeated measures approach.

**Results:**

The 660 substudy participants were broadly representative of the 6609-participant trial population. Substudy mean baseline absolute “Fluid Overload” was 0.4±1.7 L. Compared to placebo, the overall mean absolute “Fluid Overload” difference among those allocated empagliflozin was -0.24 L (95%CI -0.38, -0.11), with similar-sized differences at 2- and 18-months, and in pre-specified subgroups. Total body water differences comprised between-group differences in extracellular water of -0.49 L (95%CI -0.69, -0.30, including the -0.24 L “Fluid Overload” difference); and a -0.30 L (95%CI -0.57, -0.03) difference in intracellular water. There was no significant effect of empagliflozin on bioimpedance-derived adipose tissue mass (-0.28 [95%CI -1.41, 0.85] kg). The between-group difference in weight was -0.7 kg (95%CI -1.3, -0.1).

**Conclusions:**

In a broad range of patients with CKD, empagliflozin resulted in a sustained reduction in a bioimpedance-derived estimate of fluid overload, with no statistically significant effect on fat mass.

## Key Abbreviations And Acronyms

ATM: Adipose tissue mass
BCM: Body Composition Monitor
FTI: Fat tissue index
LTI: Lean tissue index
LTM: Lean tissue mass
MMRM: Mixed model repeated measures

## Introduction

**Figure 1:**
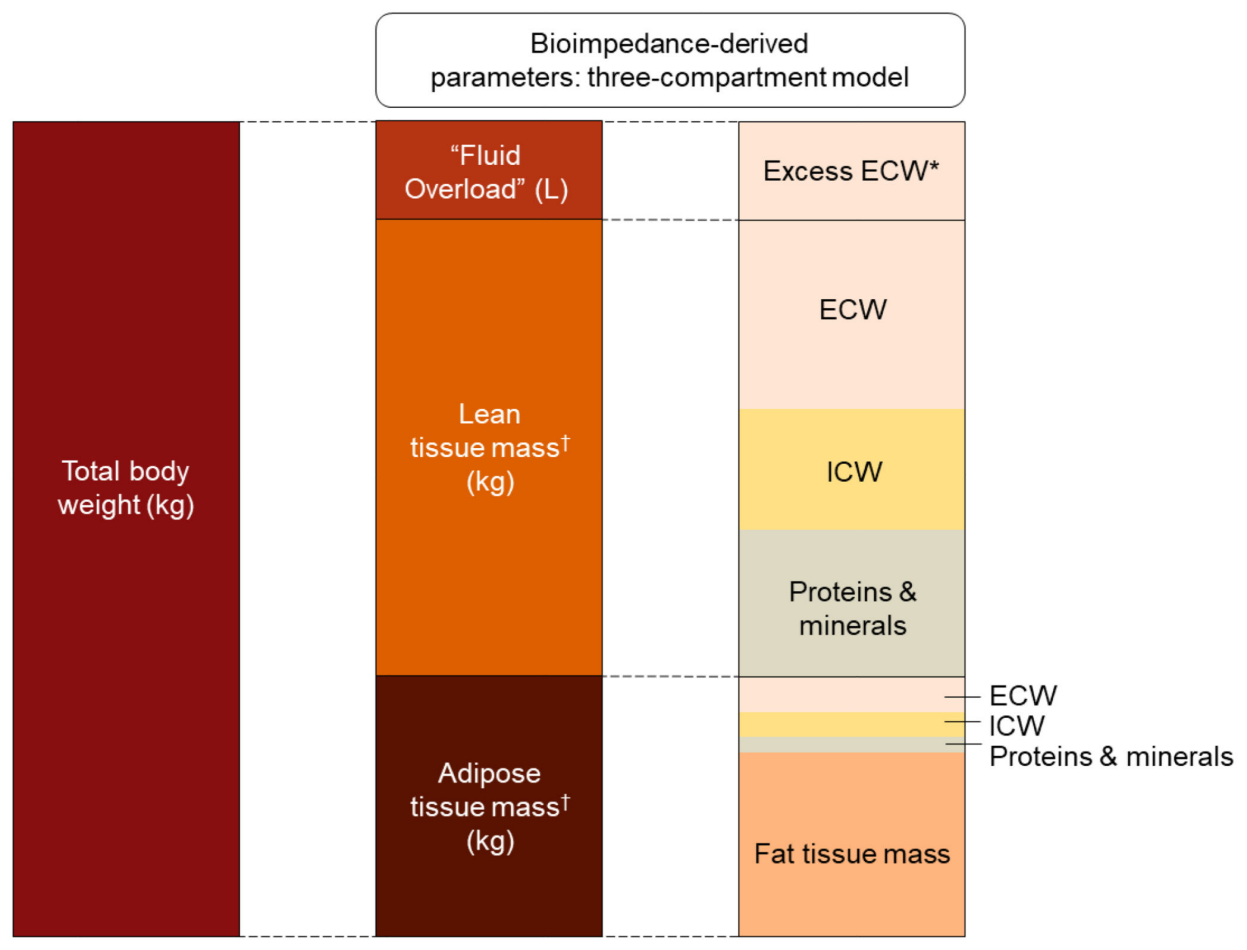
Relationship of the derived “Fluid Overload” parameter to body weight and tissue mass. Based upon the three-compartment model described by Chamney *et al*.^9^ *Excess ECW accumulates both in tissues and the blood (although blood volume is not specifically conceptualized in the three-compartment model), so changes in fluid overload could reflect changes in excess ECW that might be residing in adipose tissue, lean tissue or both. ^†^ Refers to normally-hydrated lean and adipose tissue mass. ECW = extracellular water; ICW = intracellular water. Total body water (TBW) is the sum of ECW and ICW although TBW is not conceptualized in the three-compartment model. The figure is not to scale since compartment proportions vary between individuals and “Fluid Overload” is usually smaller than depicted (and can be a negative value in fluid depletion). The mean baseline values in the EMPA-KIDNEY substudy were: total body weight 88.8 kg; “Fluid Overload” 0.4 L; lean tissue mass 38.8 kg; and adipose tissue mass 49.6 kg. In the EMPA-KIDNEY substudy, mean total ECW at baseline was 18.7 L and ICW 20.4 L.

Patients with chronic kidney disease (CKD) are at increased risk of cardiovascular disease,^1,2^ key features of which are structural heart disease, heart failure and sudden death.^3–5^ These risks increase progressively as estimated glomerular filtration rate (eGFR) decreases,^6^ with risk of death from cardiovascular disease exceeding risk of progression to kidney failure for many people with CKD. Fluid excess is common in CKD, especially when heart failure coexists,^7^ and can be quantified using bioimpedance spectroscopy.^8^ Bioimpedance can estimate a number of fluid and adiposity-related parameters, including the excess constituent of total body extracellular water (ECW) over and above what is considered normohydration. We refer to this parameter as “Fluid Overload” (see Figure 1 and the Supplemental Methods for more details about bioimpedance spectroscopy and a glossary of fluid-related terms).^9^ “Fluid Overload” can be used to guide dialysis prescription,^10^ and epidemiologically there are positive associations between bioimpedance-measured “Fluid Overload” with cardiovascular outcomes and mortality in patients on dialysis, with non-dialysis CKD, or with heart failure.^8^

The double-blind international multicenter EMPA-KIDNEY trial demonstrated that, compared to matching placebo, empagliflozin 10 mg once daily reduced the risk of kidney disease progression or cardiovascular death by 28% (95% CI 18-36%) in 6609 patients with CKD at risk of progression.^11^ A meta-analysis of large placebo-controlled trials extended these findings and showed that in people with CKD, heart failure, or type 2 diabetes at high cardiovascular risk, SGLT2 inhibitors safely reduce the risk of kidney disease progression by about two-fifths and acute kidney injury by about a quarter, with consistent effects irrespective of diabetes status.^12^ SGLT2 inhibitors also reduce the risk of cardiovascular outcomes, particularly hospitalization for heart failure.^12^ These absolute cardiovascular benefits are particularly large in patients with pre-existing heart failure,^12,13^ but smaller numbers of cardiovascular events in patients with CKD without diabetes and at low levels of eGFR mean effects are less certain in these populations.^11,12^ The amount of glycosuria induced by SGLT2 inhibition falls with decreasing eGFR and with ambient normoglycemia,^14^ so it is reasonable to hypothesize that other effects of SGLT2 inhibitors could also be attenuated in such patients.^11,15^ To address uncertainty about the effects of SGLT2 inhibitors on fluid status and adiposity in CKD, we embedded a bioimpedance-based substudy within the EMPA-KIDNEY trial.^11^ The primary aim was to assess the effects of empagliflozin 10 mg once daily versus placebo on fluid status using the bioimpedance-derived parameter of absolute “Fluid Overload” (i.e. estimated excess extracellular water). We also aimed to assess effects on this “Fluid Overload” parameter over time and in different types of patients with CKD. In this report, we also put the substudy findings with respect to empagliflozin’s effects on bioimpedance-derived fluid and adiposity parameters in the context of its potentially related effects on weight, blood pressure, glycated hemoglobin and hematocrit (as observed in the full trial cohort).

## Methods

### Substudy design and population

The full methods of the EMPA-KIDNEY trial and the main results have been reported elsewhere (ClinicalTrials.gov number, NCT03594110; EudraCT number, 2017-002971-24).^11,16^ Briefly, patients with CKD at risk of progression were identified based on historical and screening local laboratory measurements of an eGFR ≥20 but <45 mL/min/1.73m^2^, or an eGFR ≥45 but <90 mL/min/1.73m^2^ with a urinary albumin-to-creatinine ratio (uACR) ≥200 mg/g. This report details the results of an optional substudy conducted in a subset of sites in the United Kingdom (UK) and Germany which added bioimpedance measurements at the randomization, 2- and 18-month follow-up visits to the trial’s main protocol-specified procedures (Substudy Protocol Supplement available in the Supplemental Materials). All participants provided written informed consent. Regulatory authorities, as well as ethics committees at each center, approved the trial and the substudy which adhere to the Declaration of Helsinki.

### Bioimpedance measurements

Bioimpedance spectroscopy is a tool used in the clinical care of patients requiring dialysis to monitor fluid status.^17^ We employed the Fresenius Medical Care Body Composition Monitor (BCM) bioimpedance spectroscopy device as it has been extensively validated for fluid status assessment in kidney failure populations and used in randomized controlled trials.^18–20^ The device passes low level electrical current at frequencies of 5-1000 kHz (with results extrapolated from zero to infinity kHz) between electrodes attached to patients’ hands and feet.^8^ All substudy bioimpedance measurements were performed by trained local research coordinators. Body fluid and adiposity indices were then derived centrally using age, sex, a paired weight measurement, and height data combined with bioimpedance measurements of electrical resistance, and a validated three-compartment model formula using proprietary coefficients.^9,21^

The primary outcome was based on the bioimpedance-derived estimate of excess extracellular water which we refer to as absolute “Fluid Overload” (sometimes referred to as “overhydration”). It is reported in Litres and can have positive or negative values (Figure 1). Its reference range estimated from the 10^th^ and 90^th^ centiles of a reference general population distribution is -1.1 L to +1.1 L.^22^ “Fluid Overload” can be indexed to extracellular water volume and referred to as percentage relative “Fluid Overload”. An absolute value of +1.1 L approximately corresponds to relative “Fluid Overload” of +7%.^23^ Values above this threshold have been consistently associated with increased risk of death and cardiovascular events,^8^ and we refer to it as moderate “Fluid Overload” (>7%, ≤15%) or severe “Fluid Overload” (>15%).^8,23,24^ Bioimpedance measurements were also used to derive estimates of extracellular and intracellular water volume; lean tissue index (LTI) and fat tissue index (FTI) (see Supplemental Methods for more details).

Local research coordinators were trained to repeat measurements when the BCM device’s automated quality score (the Q value) was below 80 (out of 100). Visual inspection of reactance versus resistance plots (known as Cole-Cole plots) were additionally used to assess data quality.^25^ It was not always possible to obtain a Q value ≥80, so any measurement with a Q value <80 had its Cole-Cole plot assessed independently by two researchers to determine data quality and inclusion in the primary assessment using pre-specified rules blind to treatment allocation (see pre-specified Data Analysis Plan for details). Absolute “Fluid Overload” values lower than -5 L were consistently associated with low quality bioimpedance measurement and were considered invalid.

### Outcomes

The substudy’s pre-specified primary outcome was the effect of empagliflozin versus placebo on mean absolute “Fluid Overload” averaged over time, with effects on relative “Fluid Overload” provided for completeness. It was estimated that at least 382 participants would provide >90% power (at a two-sided P value of 0.05) to detect at least a 0.3 L difference in absolute “Fluid Overload” between treatment groups. The key secondary outcome was the effect of empagliflozin versus placebo on time to the first event of a cardiovascular composite defined as death from heart failure, heart failure hospitalization, or development of new moderate or severe “Fluid Overload” (in participants without this level of “Fluid Overload” at baseline). The other secondary outcomes were the effects of empagliflozin versus placebo on “Fluid Overload” at the different measurement time points. Tertiary assessments are detailed in the Supplemental Methods and include analyses of the effects of empagliflozin versus placebo on all extracellular (of which “Fluid Overload” is a constituent) and intracellular water. In addition, the effects of empagliflozin versus placebo on total body water (the sum of all extracellular and intracellular water) were assessed as a *post-hoc* analysis to contextualise effects on “Fluid Overload”.

In order for inferences from the bioimpedance substudy to be put in the context of findings from all the available EMPA-KIDNEY data, additional analyses included assessments of the effects of empagliflozin versus placebo on weight, body mass index (BMI), waist-to-hip ratio, glycated hemoglobin, hematocrit and blood pressure (systolic and diastolic) in the full trial cohort. Analyses emphasized results of study-average effects including all available measurements from routine trial visit time points (with effects at 2 and 18 months also presented). The full cohort results are emphasized due to greater statistical power and wider generalizability than the substudy. Substudy results were compared to results from the full cohort using standard statistical tests of heterogeneity. Analyses of weight and systolic blood pressure also considered results for the same subgroups as the substudy (plus self-reported race – to explore effects by race in the full trial cohort since the substudy took place in the UK and Germany only). Pre-specified sensitivity analysis for the primary outcome included three analyses assessing any effect of data quality assessments.

Analyses of effects of empagliflozin on diuretic use were included *post-hoc*.

### Statistical analysis

Substudy analyses followed the intention-to-treat principle and required a consenting participant to have provided at least one valid bioimpedance measurement. The primary outcome was pre-specified to be assessed using a mixed model repeated measures (MMRM) approach adjusted for age, sex, prior diabetes, eGFR, and uACR in the categories used in the minimized randomization algorithm.^11^ The MMRM model also included fixed categorical effects of time (to avoid assuming a linear association between treatment allocation and “Fluid Overload” over time), treatment allocation, treatment-by-time interaction, and continuous effects of baseline (randomization) measurements, and baseline-by-time interaction. The within-person error correlations were assumed to be unstructured. Analyses of the full trial cohort were additionally adjusted for region.^11^ Effects at each follow-up time point were estimated and used to derive study-average effects (with weights proportional to the amount of time between visits). All between-group differences are reported as empagliflozin minus placebo. To assess effect modification, subgroup-specific treatment effects were estimated by fitting interaction terms in the MMRM models. The null hypothesis was that the treatment effect is the same across all subgroups. This was tested by calculating a heterogeneity or trend statistic from subgroup-specific means and standard errors, without correction for multiplicity of testing.

The key secondary outcome and its components were analysed using an adjusted Cox proportional hazards regression using the same covariates in the minimization algorithm (age, sex, prior diabetes, eGFR and uACR) and included the complete substudy population of 660 participants (i.e. it included participants without a valid follow-up bioimpedance measurement who were excluded from MMRM analyses but were at risk of clinical outcomes). Tertiary analyses used the same MMRM approach as described for the primary outcome and assessed effects on ECW, ICW, LTI, FTI, body weight and BMI. Waist and hip circumference measurements were obtained at a single follow-up time point (18 months) and were therefore analysed by analysis of covariance (ANCOVA), adjusted for the baseline value and minimization variables. Handling of missing data is outlined in the Supplemental Methods. P values for hypothesis testing for outcomes are limited to the primary outcome. P values for testing for any evidence of effect modification between subgroups, and between treatment effect and effects by time are provided. The pre-specified Data Analysis Plan is provided in the Supplemental Materials. Analyses were performed using R Studio version 4.2.2 (RStudio: Integrated Development for R. RStudio, PBC, Boston, MA) and SAS version 9.4 (SAS Institute, Cary NC).

## Results

### Substudy baseline characteristics and adherence

Between 22^nd^ May 2019 and 14^th^ April 2021, 668 participants consented to join the substudy. One was excluded due to a metal knee implant and no usable bioimpedance measurement at baseline excluded a further seven, leaving 660 included in analyses (Figure S1, Supplemental Material). MMRM analyses excluded 40 consenting participants with no valid follow-up bioimpedance measurement (empagliflozin versus placebo: 21 versus 19 respectively; 3 due to death before first follow-up measurement, 28 with no follow-up measurement performed [e.g. due to COVID-19 precluding visits] and 9 due to low data quality). This left a total of 620 participants from which 1047 valid follow-up bioimpedance measurements were available for MMRM analyses.

In the substudy, mean age was 64 (15) years and 205 (31%) participants were female (Table 1). At recruitment, 136 (21%) reported a diagnosis of heart failure and 256 (39%) had diabetes. Mean (SD) eGFR was 36.0 (12.4) mL/min/1.73m^2^ and median (Q1-Q3) N-terminal pro B-type natriuretic peptide (NT pro-BNP) was 211 (93-581) ng/L. Mean body weight was 88.8 (19.8) kg and mean BMI was 30.3 (6.2) kg/m^2^. Mean absolute “Fluid Overload” at baseline was 0.4 (1.7) L with 126 (19%) and 30 (5%) participants with evidence of moderate and severe “Fluid Overload”, respectively (Table 1). Severity of “Fluid Overload” mirrored established markers of fluid excess: heart failure was twice as common in those with severe “Fluid Overload” compared to the normohydrated group, and NT-proBNP was five-fold higher (Table S2). Additionally, participants with “Fluid Overload” were more likely to be older, be male, to have prior diabetes, and have a lower eGFR (Table S2). The substudy cohort characteristics were broadly representative of the full trial cohort,^11^ although were less racially diverse due to being conducted only in the UK and Germany (Table S3).

Substudy adherence to study treatment was consistent with adherence in the full trial population.^11^ At 12 months of follow-up (the approximate midpoint of the trial), of substudy participants who remained alive, 282/318 (88.7%) in the empagliflozin group and 292/320 (91.3%) in the placebo group reported taking at least 80% of their allocated study treatment.

### Effects on bioimpedance-derived parameters

**Figure 2:**
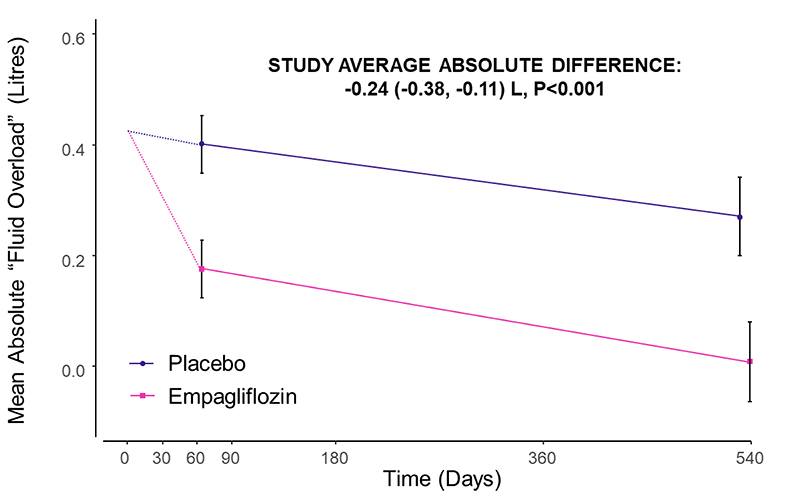
Effects of empagliflozin on mean bioimpedance-derived absolute “Fluid Overload” by time. The value at time 0 is the average across all randomized participants. Follow up means (and their CIs) are derived from a repeated measures mixed model adjusted for baseline values, age, sex, diabetes, eGFR and uACR. Follow-up values are plotted at the median follow-up day in each time window. There was no significant interaction between treatment allocation and time (p=0.11). The study average is the between-group difference (empagliflozin minus placebo) in weighted averages of both time points (see Supplemental Methods). Analyses excluded 40 consenting participants with no valid follow-up measurements. Median (Q1-Q3) follow-up since randomization for empagliflozin vs placebo groups at the 2-month visit: 64 (57-74) vs 64 (57-75) days, Wilcoxon rank sum p = 0.871; and at the 18-month visit: 540 (519-555) vs 532 (505-554) days, p = 0.026.

**Figure 3:**
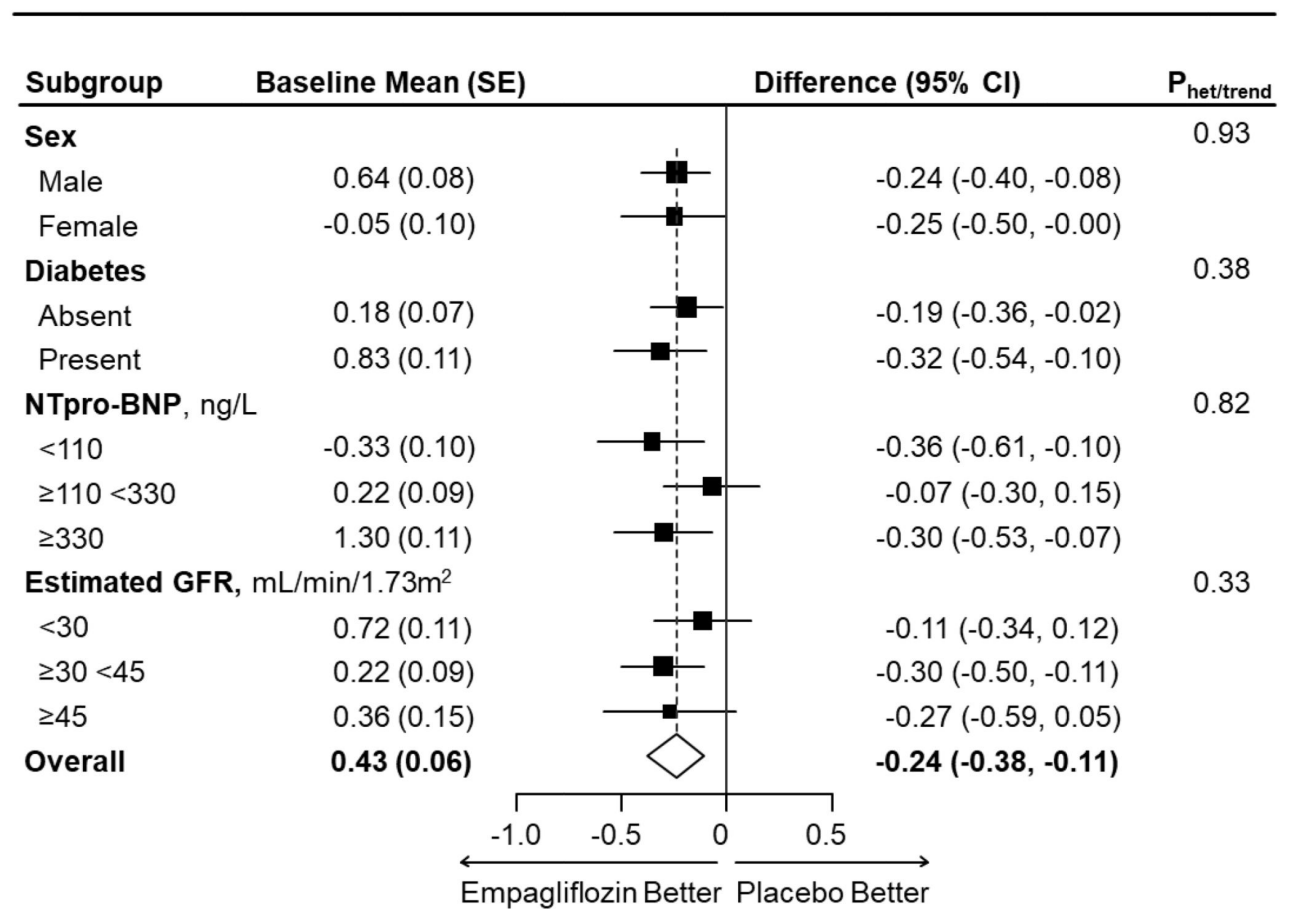
Effects of empagliflozin on mean bioimpedance-derived absolute “Fluid Overload” (in Litres) by pre-specified substudy subgroups. Study-average differences are adjusted for baseline values of the dependent variable (in continuous form) and for any differences in key baseline characteristics (categories of age, sex, diabetes, estimated GFR and urinary albumin-to-creatinine ratio) between treatment groups and weighted in proportion to the amount of time between follow-up visits (see Supplemental Methods). Analysis excluded 40 consenting participants with no valid follow-up measurements (3 deaths before first follow-up measurement, 28 with no measurement performed and 9 excluded due to inadequate data quality). Further details are available in Supplemental Table S5. Abbreviations: NTpro-BNP = N-terminal pro B-type natriuretic peptide; GFR = glomerular filtration rate.

The primary assessment found that the study-average mean absolute “Fluid Overload” was 0.24 L lower in those allocated empagliflozin compared to placebo (absolute difference in means -0.24 L, 95% CI -0.38, -0.11), with similar differences at 2 months (-0.23 L, 95% CI -0.37, -0.08) and 18 months (-0.26 L, 95% CI -0.46, -0.06) (Table 2, Figure 2). Findings were robust in sensitivity analyses assessing the effect of data quality assessments (Table S4). The effect of empagliflozin on the primary outcome was similar in subgroups by sex, diabetes status, and across the spectrum of NT-proBNP and eGFR studied (p-values for heterogeneity or trend >0.3, Figure 3 & Table S5). Neither was there any evidence of heterogeneity in *post-hoc* exploratory subgroups divided by baseline fluid status (fluid depletion, low- and high-normohydration, moderate and severe “Fluid Overload”; p=0.71), diuretic use (p=0.07) or urinary albumin-to-creatinine ratio (p=0.33, Figure S2).

There was no significant difference in the composite outcome between treatment groups (empagliflozin 35/332 [11%] versus placebo 38/328 [12%], hazard ratio (HR) 0.91, 95% CI 0.57-1.45, p=0.69) with consistent effects for its components (Table 3). The number of outcomes was low, limiting statistical power: development of new moderate “Fluid Overload” occurred in 7.8% of substudy participants allocated empagliflozin versus 10.1% allocated placebo; and development of new severe “Fluid Overload” occurred in 2.6% versus 1.3% of empagliflozin and placebo groups, respectively. The tertiary outcome of regression of moderate or severe “Fluid Overload” did not differ significantly between the empagliflozin and placebo groups (54.8% versus 48.6%; Table 3). Heart failure events were also infrequent; there were no deaths due to heart failure in the substudy population. In the full trial cohort, hospitalization for heart failure occurred in 2.7% and 3.2% of participants allocated empagliflozin and placebo, respectively (HR 0.80, 95% CI 0.60-1.06); and findings from the substudy cohort considered in isolation were consistent (empagliflozin 3.3% versus placebo 4.9%; HR 0.67, 95% CI 0.31-1.46; Table 3).

Bioimpedance estimated that the study-average absolute difference in total body water was -0.82 L (-1.24, -0.40). This consisted of differences in extracellular water of -0.49 L (95% CI -0.69, -0.30) (of which the -0.24 L between-group difference in “Fluid Overload” is a constituent); and intracellular water of -0.30 L (95% CI -0.57, -0.03). There were no significant between-group differences in bioimpedance-derived fat or lean tissue index or related tissue mass parameters (lean, fat and adipose tissue mass in kg; Tables 2, S6 & S7). In the bioimpedance substudy population, the study-average between-group difference in weight was -0.7 kg (-1.3, -0.1).

### Effects on anthropometry, blood pressure and relevant laboratory values in the full trial cohort

**Figure 4:**
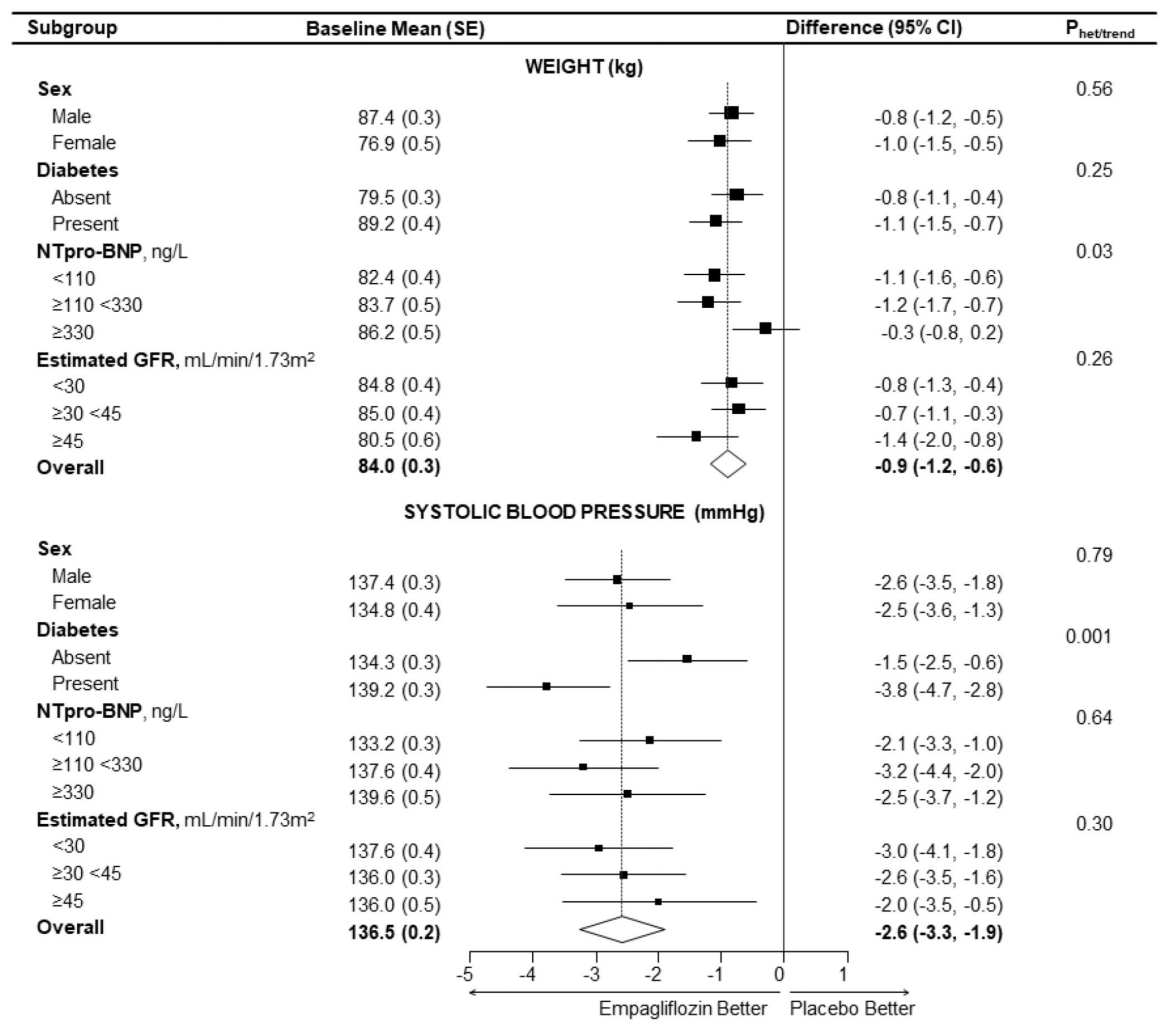
Full trial cohort: effects of empagliflozin on weight and systolic blood pressure overall and by key bioimpedance substudy pre-specified subgroups. Study-average differences are adjusted for baseline values of the dependent variable (in continuous form) and for any differences in key baseline characteristics (categories of age, sex, diabetes, estimated glomerular filtration rate, urinary albumin-to-creatinine ratio and region) between treatment groups and weighted in proportion to the amount of time between follow-up visits (see Supplemental Methods). Each analysis includes all individuals with at least one follow-up measurement of the outcome variable with mean imputation of missing baseline measurements. For comparison, between-group differences in the substudy cohort were -0.7 (95% CI -1.3, -0.1) kg and -3.3 (-5.5, -1.2) mmHg for weight and systolic blood pressure, respectively.

In the full trial cohort, the between-group difference in weight was -0.9 kg (95% CI -1.2, -0.6) (Figure 4, Table S8) and the effect of empagliflozin on weight did not vary significantly over time (interaction p value by time=0.47, Table S8). In the full cohort, there was no evidence of heterogeneity of the effect of empagliflozin on weight in subgroups by sex, baseline eGFR or diabetes (Figure 4, or in *post-hoc* analyses by race: Figure S3). Waist-to-hip ratio at 18 months was also not significantly different between the empagliflozin versus placebo groups (Table S9). The study-average difference in HbA1c in the full cohort was -0.4 mmol/mol (95% CI -0.8, -0.0), with a -0.9 mmol/mol (95% CI -1.6, -0.1) difference in HbA1c in participants with diabetes at randomization and no significant difference in participants without diabetes (0.0 mmol/mol, 95% CI -0.2, 0.2; Table S10). The full trial cohort average between-group difference in hematocrit at 18 months post-randomization was 2.3% (95% CI 1.9, 2.7).

The study-average between-group differences in systolic and diastolic blood pressure were -2.6 mmHg (95% CI -3.3, -1.9) and -0.5 mmHg (95% CI -0.9, -0.1), respectively. In the full trial cohort, there was no evidence of heterogeneity of the effect of empagliflozin on systolic blood pressure when subdivided by sex, baseline eGFR, NTpro-BNP (Figure 4) or race (Figure S3), but there was some evidence to suggest a larger systolic blood pressure difference in patients with diabetes (Figure 4). Effects on anthropometry, HbA1c, hematocrit and blood pressure in the substudy were approximately consistent with the full trial cohort results (Tables S8-S11).

### Effects on diuretic use

Among those participants in the full trial cohort who were not taking a loop diuretic at randomization, 159/2453 (6.5%) in the empagliflozin group compared to 212/2409 (8.8%) in the placebo group started such medication during follow-up, representing a 26% lower likelihood of a new loop diuretic prescription among the empagliflozin group (risk ratio 0.74, 95% CI 0.60-0.90).

## Discussion

In the EMPA-KIDNEY substudy of 660 patients with CKD, empagliflozin resulted in a sustained reduction in bioimpedance-derived “Fluid Overload” for at least 18 months, irrespective of diabetes status or level of kidney function. Using the three-compartment model, we observed a -0.24 L between-group difference in “Fluid Overload” but no significant differences in normally-hydrated lean or adipose tissue compartments. Fluid volume differences consisted of ~0.8 L less total body water of which ~0.5 L was extracellular and ~0.3 L intracellular water (with the ~0.5 L total extracellular water difference including the -0.24 L between-group difference in excess extracellular water referred to as “Fluid Overload”). These data raise a hypothesis that an important determinant of the substudy -0.7 kg weight difference was due to effects on fluid status. Along with other mechanisms,^26^ this effect may contribute to the cardiovascular benefits of SGLT2 inhibitors.

Osmotic diuretic and natriuretic actions are considered potentially important contributing mechanisms to the cardiovascular benefits of SGLT2 inhibitors, but their effect on fluid status in CKD – where effects may be hypothesized to be attenuated by decreased kidney function – have not previously been quantified in randomized trials.^15,26–28^ In patients with type 2 diabetes without kidney disease, mechanistic trials have reported plasma volume reductions by SGLT2 inhibitors,^30^ and raised a hypothesis that SGLT2 inhibitors reduce interstitial volume more than plasma volume.^28^ Previously collected bioimpedance data in patients taking SGLT2 inhibitors is limited to mainly non-randomized studies.^31–34^ To the best of our knowledge, the 16-week DECREASE trial provides the only peer-review published randomized evidence on the effects of SGLT2 inhibitors on bioimpedance parameters to date. It found that, in 66 participants with type 2 diabetes - CKD status not reported - dapagliflozin reduced extracellular fluid by ~1 L and systolic blood pressure by ~4 mmHg at 10 days versus placebo.^35^ EMPA-KIDNEY now substantially extends these previous findings by studying longer term effects (over 18 months) in a much larger number of participants in a placebo-controlled trial.

Before the results of this substudy, attenuation of diuretic effects at low levels of kidney function was considered plausible as SGLT2 inhibitors have little effect on glycemia at lower eGFR due to attenuated levels of glycosuria.^11,14,36–38^ Despite this, we found consistent effects on “Fluid Overload” across the eGFR-based subgroups. Similarly, effects did not vary by baseline fluid status, diuretic use or albuminuria. These findings are analogous to results from large randomized trials in heart failure populations that included a large proportion of patients with CKD and low eGFR and demonstrated consistent effects of SGLT2 inhibitors on cardiovascular death or hospitalization for heart failure irrespective of sex, diabetes, eGFR or NTpro-BNP at baseline.^13^

It is also relevant that the effect of empagliflozin on fluid loss in EMPA-KIDNEY was achieved safely. Although estimates of extracellular water reduction reflected loss of extracellular water that is not considered to be in excess by the three-compartment model, there was no increased risk of participant reports of symptomatic dehydration in the full trial or substudy cohorts (Table S12), nor any increased risk of acute kidney injury.^39^

We also report assessments of the effects of empagliflozin on anthropometry, blood pressure, HbA1c and hematocrit for the full trial and substudy cohorts, with the full trial data providing better statistical power to assess for any effect modification between subgroups of participant. The effects of empagliflozin on weight and HbA1c in EMPA-KIDNEY are generally consistent with results from other CKD trials. CREDENCE studied 4401 participants with type 2 diabetes and a mean eGFR of 56 mL/min/1.73m^2^_._ Compared with placebo, mean weight was 0.80 kg (95% CI 0.69-0.92) lower in the canagliflozin group, and there was a relatively modest difference in HbA1c (-0.25%, 95% CI -0.20, -0.31).^41^ The DAPA-CKD trial studied 4304 participants with a mean eGFR of 43 mL/min/1.73m^2^, and included 2996 participants with diabetes.^42^ The between-group difference in HbA1c in those with diabetes was -1.1 mmol/mol (95% CI –2.1, 0.0).^43^ The overall between-group difference in systolic blood pressure in EMPA-KIDNEY of -2.6 mmHg (95% CI -3.3, -1.9) was also similar to the other large CKD trials: CREDENCE difference -3.3 mmHg (95% CI -2.7, -3.9),^41^ and DAPA-CKD difference -2.9 mmHg (95% CI -3.6, -2,3).^44,45^ In EMPA-KIDNEY there were somewhat larger antihypertensive effects in participants with diabetes (heterogeneity p=0.001). This pattern was not observed in bioimpedance-derived “Fluid Overload” analyses, raising the hypothesis that SGLT2 inhibition may have additional antihypertensive effects which are more prominent in patients with diabetes, and which are distinct from their diuretic effects (possibly through effects on vascular stiffness or endothelial function).^46–48^ The lack of measured effect of empagliflozin on adiposity is consistent with its modest effects on glycated haemoglobin observed in the CKD population.

## Study limitations

EMPA-KIDNEY demonstrated the clear benefits of SGLT2 inhibition on kidney disease progression in a wide range of patients with CKD at risk of progression, including about a one-third reduction in the risk of needing to start kidney replacement therapy.^11^ This large EMPA-KIDNEY substudy benefits from its sample size, long duration, systematic measurements and randomized double-blind design. These help ensure between-group differences are unbiased and reliable. The BCM device has some technical limitations. For example, BCM parameters are derived and not direct measurements and based on formulae normalized to healthy reference populations and estimations may be less accurate at extremes of “Fluid Overload” (although extremes of levels were uncommon in the substudy population). Furthermore, imprecision in fat mass estimates mean the lack of statistical effect on fat mass does not exclude some effect (Supplementary Figure 4). BCM also does not reliably assess subtypes of adiposity (e.g. visceral versus peripheral). Follow-up was affected by COVID-19 restrictions resulting in some missed bioimpedance measurements, and the pre-specified key secondary composite analysis was underpowered due to lower cardiovascular risk in the trial population than was predicted during its design. Nevertheless, this substudy collected sufficient data to provide reliable and clear results for the primary and other continuously measured outcomes. Due to the regions contributing to the substudy, Asian, Black, Mixed and Other races were under-represented, but effects on weight, HbA1c, and blood pressure for the full trial cohort were broadly similar to the substudy results across the studied races, suggesting our conclusions are likely to be generalizable. Lastly, use of other diuretics was determined by local doctors and not controlled by the protocol. We observed more new use of loop diuretics among those allocated to placebo, so the presented estimates of effects on fluid parameters, weight and blood pressure may be slight underestimates of the full effect of empagliflozin.

In summary, the EMPA-KIDNEY bioimpedance substudy found that fluid excess is common in a broad population of patients with CKD at risk of progression, and that empagliflozin resulted in sustained reductions in “Fluid Overload”, weight and blood pressure in patients with CKD with and without diabetes, even in patients with low levels of kidney function.

## Disclosures

The EMPA-KIDNEY bioimpedance substudy was initiated, designed, conducted analysed and reported by the University of Oxford with a Steering Committee of experts. This paper has not been published previously in whole or part. The Clinical Trial Service Unit and Epidemiological Studies Unit (Oxford, UK) has a staff policy of not accepting honorarium or other payments from the pharmaceutical industry, except for the reimbursement of costs to participate in scientific meetings (see https://www.ctsu.ox.ac.uk/about/ctsu_honoraria_25june14-1.pdf).

KJM, NS, PKJ, DP, JE, RD, RL, JN, AO, MJL, CB, RH and WGH report grant funding paid to their institution (the University of Oxford) from Boehringer Ingelheim and Eli Lilly, and funding from the United Kingdom Medical Research Council (MRC) (to the Clinical Trial Service Unit and Epidemiological Studies Unit; reference no., MC_UU_00017/3), the British Heart Foundation, National Institute for Health and Care Research Biomedical Research Council, and Health Data Research (UK). WGH was additionally funded by an MRC Kidney Research UK Professor David Kerr Clinician Scientist Award (MR/R007764/1). DFK reports institutional grant funding from Baxter Healthcare for an Investigator Initiated Research Grant Award. CW reports institutional grant funding from Boehringer Ingelheim and Sanofi; consulting fees from Bayer, Boehringer Ingelheim, Astra Zeneca and Astellas; honoraria for lectures from Bayer, Boehringer Ingelheim, Astra Zeneca, Amgen, Sanofi, MSD, Fresenius Medical Care and CSL Vifor. SB reports participation on a Nephrologist Advisory Board of Boehringer Ingelheim. VC reports support to attend meetings from Boehringer Ingelheim, Lilly and the University of Oxford. JS reports grant funding and honoraria from Boehringer Ingelheim and Astra Zeneca; and support to attend meetings from Boehringer Ingelheim (Annual meeting of the German Society of Nephrology). DT has nothing to disclose. JBG reports grant funding from Merck, Boehringer Ingelheim, Roche and Lilly; consulting fees from Boehringer Ingelheim, Lilly, Bayer, NovoNordisk, Pfizer, Merck, AstraZeneca, Anji and Valo Vertex; honoraria for lectures from Boehringer Ingelheim; support to attend meetings from Bayer, Novo Nordisk and Lilly; andwriting support from Bayer. DZIC reports grant funding from Astra Zeneca, Boehringer Ingelheim, Eli Lilly, Janssen, Merck & Co. Inc., Sanofi, CSL-Behring and Novo Nordisk; and consulting fees from Astra Zeneca, Bayer, Boehringer Ingelheim, Eli Lilly, Janssen, Merck & Co. Inc., Mitsubishi-Tanabe, Novo Nordisk, Prometic, Sanofi, Abbvie, BMS, Maze, Gilead, CSL-Behring, Otsuka, Novartis, Youngene, Lexicon and Inversago. LSH reports support to attends meetings from the Malaysian Society of Nephrology and unpaid leadership roles as Editor, the Malaysian Dialysis Transplant Registry; and Chairman, Nephrology Medical Education Committee of the Malaysian Medical Council. RP reports honoraria from Astra Zeneca, Boehringer Ingelheim, Menarini, Lilly, MSD, Novartis, Alfa-Sigma and Novo Nordisk. KRT reports grant funding from the National Institutes of Health, NIH (NIDDK, NHLBI, NCATS, NIMHD), the Centers for Disease Control and Prevention (CDC), Travere and Bayer; consulting fees from Lilly, Boehringer Ingelheim, Astra Zeneca, Novo Nordisk, Travere, Bayer and Pfizer; honoraria from Lilly, Astra Zeneca, Novo Nordisk and Bayer; support to attend meetings from Novo Nordisk; unpaid roles on Data Safety Monitoring/Advisory Boards for NIDDK and George Clinical; and unpaid leadership roles as Chair, Diabetic Kidney Disease Collaborative, American Society of Nephrology and Board of Directors, Kidney Health Initiative. JSL reports personal lectureship honoraria from Astra Zeneca. PBM reports grant funding (paid to institution) from Astra Zeneca and Boehringer Ingelheim; consulting fees(paid to institution) from GSK, Astellas, Bayer, Astra Zeneca and Boehringer Ingelheim; honoraria (paid to institution) from Astra Zeneca, Boehringer Ingelheim and Pharmacosmos; and personal payment from Novartis for participation on a Data Safety Monitoring Board or Advisory Board. SJD has nothing to disclose. SJH, DS and MB are employees of Boehringer Ingelheim International GmbH.

## Supplementary Material

Supplement

## Figures and Tables

**Table 1:** Bioimpedance substudy cohort: baseline characteristics

	Empagliflozin (N=332)	Placebo (N=328)
**Demographics**		
**Age** (years)	65.2 (14.2)	64.1 (14.9)
**Female sex**	102 (30.7)	103 (31.4)
**White race**	321 (96.7)	315 (96.0)
**Prior Disease**		
**Diabetes**	135 (40.7)	121 (36.9)
**Heart failure**	62 (18.7)	74 (22.6)
**Clinical Measurements**		
**Weight** (kg)	89.8 (20.2)	87.9 (19.3)
**Body mass index** (kg/m^2^)	30.5 (6.2)	30.1 (6.3)
**Waist-to-hip ratio**	1.0 (0.1)	1.0 (0.1)
**Systolic blood pressure** (mmHg)	137.0 (18.8)	137.5 (18.9)
**Diastolic blood pressure** (mmHg)	77.8 (12.2)	78.6 (11.9)
Bioimpedance Measurements*		
**Absolute “Fluid Overload”** (L)	0.45 (1.68)	0.32 (1.68)
**Relative “Fluid Overload”** (%)		
Mean (SD)	1.9 (8.7)	1.3 (8.3)
Moderate “Fluid Overload”	70 (21.1)	56 (17.1)
Severe “Fluid Overload”	14 (4.2)	16 (4.9)
**Extracellular water** (L)	19.0 (3.8)	18.4 (3.7)
**Intracellular water** (L)	20.7 (4.5)	20.1 (4.6)
**Lean tissue index** (kg/m^2^)	13.3 (3.1)	12.9 (3.0)
**Fat tissue index** (kg/m^2^)	12.6 (5.4)	12.5 (5.1)
**Laboratory Measurements**		
**Estimated GFR** (mL/min/1.73m^2^)		
Mean (SD)	36.1 (13.4)	35.8 (11.4)
Distribution		
<30	123 (37.0)	118 (36.0)
≥30 <45	148 (44.6)	154 (47.0)
≥45	61 (18.4)	56 (17.1)
**Urinary albumin-to-creatinine ratio** (mg/g)	203 (26-958)	205 (29-865)
**HbA1c** (mmol/mol)	43.9 (11.3)	43.5 (10.9)
**NTpro-BNP** (ng/L)	197 (90-596)	225 (95-550)
**Medications**		
**RAS inhibitor**	304 (91.6)	288 (87.8)
**Any diuretic therapy**	180 (54.2)	173 (52.7)

Data are presented as mean (SD) or median (Q1-Q3) for continuous variables and n (%) for categorical variables. *Bioimpedance measurements are presented for 644/660 participants with a baseline measurement (missing for 16/660) irrespective of validity for inclusion in the primary analysis. Abbreviations: GFR = glomerular filtration rate; HbA1c = glycated hemoglobin; NTpro-BNP = N-terminal pro-brain-type natriuretic peptide; RAS = renin-angiotensin system.

**Table 2:** Effects of empagliflozin on bioimpedance-derived parameters

	Empagliflozin (N=311)		Placebo (N=309)				
	Adjusted* Mean	SE	Adjusted* Mean	SE	Absolute Difference	95% CI	P value for primary outcome
**Primary Assessments**							
**Absolute “Fluid Overload”**, L							
Study average	0.10	0.05	0.34	0.05	-0.24	(-0.38, -0.11)	<0.001
**Relative “Fluid Overload”**, %							
Study average	0.14	0.25	1.33	0.25	-1.19	(-1.90, -0.48)	0.001
**Secondary Assessments**							
**Absolute “Fluid Overload”**, L							
Randomization	0.50	0.09	0.35	0.09			
2-month follow-up	0.18	0.05	0.40	0.05	-0.23	(-0.37, -0.08)	
18-month follow-up	0.01	0.07	0.27	0.07	-0.26	(-0.46, -0.06)	
**Relative “Fluid Overload”**, %							
Randomization	2.24	0.47	1.39	0.45			
2-month follow-up	0.52	0.27	1.65	0.27	-1.12	(-1.88, -0.37)	
18-month follow-up	-0.36	0.38	0.92	0.37	-1.28	(-2.32, -0.23)	
**Tertiary Assessments**							
**Extracellular Water**, L							
Study average	18.16	0.07	18.66	0.07	-0.49	(-0.69, -0.30)	
**Intracellular Water**, L							
Study average	20.10	0.10	20.40	0.10	-0.30	(-0.57, -0.03)	
**Lean Tissue Index** **(LTI)**, kg/m^2^							
Study average	12.90	0.09	13.05	0.09	-0.14	(-0.39, 0.10)	
**Fat Tissue Index (FTI)**, kg/m^2^							
Study average	12.34	0.10	12.42	0.10	-0.07	(-0.35, 0.20)	

*Mean effects are adjusted for baseline values of the dependent variable (in continuous form) and for any differences in key baseline characteristics (categories of age, sex, diabetes, estimated glomerular filtration rate and urinary albumin-to-creatinine ratio) between treatment groups with study averages weighted in proportion to the amount of time between follow-up visits (see Supplemental Methods). Analysis excluded 40 consenting participants with no valid follow-up measurements (3 deaths before first follow-up measurement, 28 with no measurement performed and 9 excluded due to inadequate data quality). Effects on “Fluid Overload” did not vary by time: p value for interaction with time = 0.11 and 0.39 for absolute and relative “Fluid Overload”, respectively.

**Table 3:** Effects of empagliflozin on cardiovascular composite outcome (bioimpedance substudy cohort)

	Empagliflozin		Placebo				
	n/N	%	n/N	%	Hazard Ratio	95% CI	P value
Key Secondary Assessment Death from heart failure, hospitalization for heart failure, development of new moderate or severe “Fluid Overload”*	35/332	10.5	38/328	11.6	0.91	(0.57-1.45)	0.69
Death from heart failure	0/332	0.0	0/328	0.0	-	-	
Hospitalization for heart failure	11/332	3.3	16/328	4.9	0.67	(0.31-1.46)	
Development of new moderate “Fluid Overload”*	18/232	7.8	25/247	10.1	0.68	(0.37-1.26)	
Development of new severe “Fluid Overload”†	8/302	2.6	4/303	1.3	1.96	(0.57-6.71)	
**Tertiary Assessment**							
Regression of “Fluid Overload”‡	46/84	54.8	35/72	48.6	1.33	(0.82-2.18)	

All analyses use a time-to-first-event approach. Cox proportional hazards models include adjustment for the covariates used in the minimization algorithm: age, sex, diabetes status, estimated glomerular filtration rate and urinary albumin-to-creatinine ratio. Results were consistent in *post-hoc* sensitivity analyses additionally adjusted for use of any diuretic or loop diuretics at baseline (hazard ratios [95% CIs] 0.89 [0.56-1.42] and 0.92 [0.58-1.47]; respectively). * Requires randomization value of relative “Fluid Overload” ≤7% and follow-up value >7%, ≤15%. † Requires randomization value of relative “Fluid Overload” ≤15% and follow-up value >15%. ‡ Requires randomization value consistent with moderate or severe relative “Fluid Overload” and regression to any lower hydration category at any follow-up (limited to first event). All 660 participants were included in the composite outcome analysis since all participants were at risk of the clinical components of the composite. In the full trial cohort there were 88 (2.7%) first hospitalizations for heart failure in the empagliflozin group versus 107 (3.2%) in the placebo group: hazard ratio 0.80, 95% CI 0.60-1.06.

## Data Availability

The complete de-identified patient data set used for presented analyses will be available in due course and the application system to apply to use data will open 6 months after publication. Departmental policy details can be found here: https://www.ndph.ox.ac.uk/data-access. In adherence with the Boehringer Ingelheim Policy on Transparency and Publication of Clinical Study Data, scientific and medical researchers can request access to clinical study data after publication of the primary manuscript and secondary analyses in peer-reviewed journals and regulatory and reimbursement activities are completed, normally within 1 year after the marketing application has been granted by major Regulatory Authorities. Researchers should use the https://vivli.org/ link to request access to study data and visit https://www.mystudywindow.com/msw/datasharing for further information.

## Significance Statement

SGLT2 inhibitors reduce risk of kidney progression, acute kidney injury and cardiovascular disease, but the mechanisms of benefit are incompletely understood. Bioimpedance spectroscopy can estimate body water and fat mass. One quarter of the EMPA-KIDNEY bioimpedance substudy CKD population had clinically significant levels of bioimpedance-derived “Fluid Overload” at recruitment. Empagliflozin induced a prompt and sustained reduction in “Fluid Overload”, irrespective of sex, diabetes, baseline NT-proBNP or eGFR. No significant effect on bioimpedance-derived fat mass was observed. The effects of SGLT2 inhibitors on body water may be one of the contributing mechanisms by which they mediate effects on cardiovascular risk.
