# Supplementary material for: Effects of Empagliflozin on Fluid Overload, Weight, and Blood Pressure in CKD
Source: J Am Soc Nephrol. 2023 Dec 12;35(2):202–15. doi: 10.1681/ASN.0000000000000271 (PMC7615589; doi:10.1681/ASN.0000000000000271)
Supplement: Supplementary file 1 [file jasn-35-202-s001.pdf]

Effects of Empagliflozin on Fluid Overload, Weight and Blood Pressure in CKD

Supplemental Materials

Contents

Members of the EMPA-KIDNEY Collaborative Group 2

Membership of the Executive Committee, Steering Committee and Independent Data Monitoring Committee .. 2

List of Collaborators, by Site 3

Supplemental Methods 6

Bioimpedance-derived parameters using the Body Composition Monitor (BCM) 6

Glossary of fluid-related terms 7

Weighting of the analyses in the bioimpedance substudy 8

Handling of missing and duplicate data 8

Data quality assessment pilot procedure in the bioimpedance substudy 10

Tertiary & exploratory *post-hoc* assessments in the bioimpedance substudy 12

Clinical assessments in the full trial cohort 12

Laboratory assessments in the full trial cohort 13

Supplemental methods references 14

Substudy Protocol Supplement 15

Substudy Data Analysis Plan 22

Supplemental Tables 41

Supplemental Table 1: Bioimpedance substudy cohort: additional baseline characteristics 42

Supplemental Table 2: Bioimpedance substudy cohort: baseline characteristics by categories of baseline bioimpedance-derived "Fluid Overload" 44

Supplemental Table 3: Baseline characteristics for the substudy, substudy region and the full trial cohorts 45

Supplemental Table 4: Sensitivity analyses for the effects of empagliflozin on mean bioimpedance-derived absolute "Fluid Overload" in L 46

Supplemental Table 5: Bioimpedance substudy cohort: unadjusted baseline means and adjusted study averages for "Fluid Overload" for each subgroup by treatment group (additional data to accompany Figure 3) 47

Supplemental Table 6: Effects of empagliflozin on tertiary (and *post-hoc*) bioimpedance-derived parameters assessments by time 48

Supplemental Table 7: Effects of empagliflozin on other bioimpedance-derived adiposity parameters 49

Supplemental Table 8: Effects of empagliflozin on weight and body mass index (bioimpedance substudy & full trial cohorts) 50

Supplemental Table 9: Effects of empagliflozin on waist and hip measurements (bioimpedance substudy & full trial cohorts) 51

Supplemental Table 10: Effects of empagliflozin on glycated hemoglobin and hematocrit (bioimpedance substudy & full trial cohorts) 52

Supplemental Table 11: Effects of empagliflozin on blood pressure (bioimpedance substudy & full trial cohorts)53

Supplemental Table 12: Bioimpedance substudy cohort: effects of empagliflozin on dehydration by categories of baseline bioimpedance-derived "Fluid Overload" 54

Supplemental Figures 55

Supplemental Figure 1: Bioimpedance substudy cohort CONSORT flowchart 56

Supplemental Figure 2: Effects of empagliflozin on mean absolute "Fluid Overload" (bioimpedance substudy cohort: post-hoc subgroups) 57

Supplemental Figure 3: Correlation between change in weight (relative to baseline) with change in different bioimpedance indices at the 2-month follow-up visit, by treatment allocation 58

Supplemental Figure 4: Effects of empagliflozin on weight, body mass index, systolic blood pressure, glycated hemoglobin, and hematocrit by race (full trial cohort) 59

Members of the EMPA-KIDNEY Collaborative Group

Membership of the Executive Committee, Steering Committee and Independent Data Monitoring Committee

Executive Committee

Colin Baigent (co-Chair), Martin J. Landray (co-Chair), Christoph Wanner (Deputy Chair), William G. Herrington (Chief Investigator), Richard Haynes (co-Principal Investigator), Jennifer B. Green, Sibylle J. Hauske*, Martina Brueckmann*, Mark Hopley*.

(Previous members: Maximillian von-Eynatten* & Jyothis George*)

Steering Committee

Executive Committee members plus National Representatives: Susanne Brenner (Germany); Alfred K. Cheung (United States); David Preiss (United Kingdom); Zhi-Hong Liu, Jing Li (China); Laiseong Hooi, Wen Liu (Malaysia); Takashi Kadowaki, Masaomi Nangaku (Japan); Adeera Levin, David Cherney (Canada); Roberto Pontremoli, Aldo P. Maggioni (Italy); plus statistician members: Natalie Staplin, Jonathan Emberson, Stefan Hantel*; plus other expert members: Shinya Goto, Rajat Deo, Katherine R. Tuttle.
Non-voting members: Michael Hill, Parminder Judge, Kaitlin J. Mayne, Sarah Y.A. Ng, Xavier Rossello, Emily Sammons, Doreen Zhu.

* denotes a Boehringer Ingelheim employee

Independent Data Monitoring Committee

Peter Sandercock (Chair), Rudolf Bilous, Charles Herzog, Paul Whelton, Janet Wittes, Derrick Bennett (non-voting statistician).

List of Collaborators, by Site

Central Coordinating Office (Clinical Trial Service Unit and Epidemiological Studies Unit [CTSU], University of Oxford)

Administration: Patricia Achiri, Chrissie Ambrose, Cristina Badin, Jill Barton, Richard Brown, Andy Burke, Sebastian Butler, Rejive Dayanandan, Pia Donaldson, Robert Dykas, Lucy Fletcher, Kate Frederick, Hannah Kingston, Mo Gray, Emily Harding, Akiko Hashimoto, Lyn Howie, Susan Hurley, Ryonfa Lee, Nik Luker, Kevin Murphy, Mariko Nakahara, John Nolan, Michelle Nunn, Sorcha Mulligan, Akiko Omata, Sandra Pickworth, YanRu Qiao, Shraddha Shah, Karen Taylor, Alison Timadjer, Monique Willett, Liz Wincott, Qin Yan, Hui Yu; ***Clinical***: Louise Bowman, Fang Chen, Robert Clarke, Michelle Goonasekera, Richard Haynes, William G. Herrington, Parminder Judge, Waseem Karsan, Marion Mafham, Kaitlin J. Mayne, Sarah Y. A. Ng, David Preiss, Christina Reith, Emily Sammons, Mohammed Zayed, Doreen Zhu; ***Data Analysis***: Ritva Ellison, Rowan Moys, Will Stevens, Kevin Verdel, Karl Wallendszus; ***Finance***: Chris Bowler, Anna Brewer, Andy Measor; ***IT Validation***: Guanguo Cui, Charles Daniels, Angela Field, Bob Goodenough, Ashley Lawson, Youcef Mostefai, Dheeptha Radhakrishnan, Samee Syed, Shuang Xia; ***Laboratory***: Ruth Adewuyi-Dalton, Thomas Arnold, Anne-Marie Beneat, Anoushka Bhatt, Chloe Bird, Andrew Breach, Laura Brown, Mark Caple, Tatyana Chavagnon, Karen Chung, Sarah Clark, Luminita Condurache, Katarzyna Eichstadt, Marta Espino Obrero, Scarlett Forest, Helen French, Nick Goodwin, Andrew Gordon, Joanne Gordon, Cat Guest, Tina Harding, Michael Hill, Michal Hozak, Matthew Lacey, David MacLean, Louise Messinger, Stewart Moffat, Martin Radley, Claire Shenton, Sarah Tipper, Jon Tyler, Lesley Weaving, James Wheeler, Elissa Williams, Tim Williams, Hamish Woodhouse; ***Monitoring***: Angela Chamberlain, Jo Chambers, Joanne Davies, Denise Donaldson, Pati Faria-Shayler, Denise Fleming-Brown, Jennifer Ingell, Carol Knott, Anna Liew, Helen Lochhead, Juliette Meek, Isabel Rodriguez-Bachiller, Andrea Wilson, Patrick Zettergren; ***Programming***: Rach AitSadi, Ian Barton, Alex Baxter, Yonghong Bu, Lukasz Danel, Sonja Grotjahn, Rijo Kurien, Michael Lay, Archie Maskill, Aleksandra Murawska, Rachel Raff, Allen Young; ***Principal Investigators***: Colin Baigent, Richard Haynes, William G. Herrington, Martin J. Landray, David Preiss; ***Statistics***: Jonathan Emberson, Rebecca Sardell, Natalie Staplin.

Regional Coordinating Centres

Germany (Universitätsklinikum Würzburg): Christoph Wanner, Susanne Brenner, Vladimir Cejka, Marcela Fajardo-Moser, Christian Hartner, Doris Poehler, Janina Renner, Franziska

Scheidemantel

United Kingdom (Clinical Trial Service Unit and Epidemiological Studies Unit [CTSU], University of Oxford): Richard Haynes, David Preiss, William G. Herrington, Parminder Judge, Doreen Zhu, Sarah Y. A. Ng, Kaitlin J. Mayne, Cristina Badin, Jo Chambers, Joanne Davies, Denise Donaldson, Mo Gray, Emily Harding, Jenny Ingell, Yanru Qiao, Shraddha Shah, Andrea Wilson, Patrick Zettergren

Local Clinical Centres

Germany: *Universitatsklinikum Wurzburg*: Christoph Wanner, Susanne Brenner, Vladimir Cejka, Sharang Ghavampour, Anja Knoppe; *Zentrum fuer Nieren-, Hochdruck- und Stoffwechselerkrankungen Hannover*: Hans Schmidt-Gurtler, Hubert Dumann, Sybille Merscher, Margret Patecki, Georg Rainer Schlieper, Anke Torp, Bianca Weber, Maja Zietz; *Klinikum der Universitat Munchen*: Thomas Sitter, Louise Fuessl, Julia Krappe, Jerome Loutan, Volker Vielhauer, Luciano Andriaccio, Magdalena Maurer; *ClinPhenomics GmbH Co. KG*: Bernhard Winkelmann, Martin Dursch, Linda Seifert, Linda Tenbusch; *Universitatsmedizin Mainz*: Julia Weinmann-Menke, Simone Boedecker, Wiebke Kaluza-Schilling, Daniel Kraus, Carina Krieger, Margit Schmude, Anne Schreiber, Ewelina Eckrich; *Herz- und Diabeteszentrum Nordrhein-Westfalen*: Diethelm Tschope, Abdulwahab Arbi, Young Lee-Barkey, Bernd Stratmann, Natalie Prib, Sina Rolfsmeier, Irina Schneider; *Universitatsklinikum Dusseldorf*: Lars Rump, Johannes Stegbauer, Christine Pötz, Mara Schemmelmann, Claudia Schmidt; *Studienzentrum fur Nieren- und Hochdruckerkrankungen Hannover*: Hermann Haller, Jessica Kaufeld, Jan Menne, Elisabeth Bahlmann-Kroll, Angela Bergner

UK: *Oxford University Hospitals*: Richard Haynes, William G. Herrington, Doreen Zhu, Madita Gavrila, Kathryn Lafferty, Ria Rabara, Sally Ruse, Maria Weetman; *Nottingham City Hospital*: Cath Byrne, Mark Jesky, Alison Cowley, Emma McHaffie, Holly Waterfall; *Dorset County Hospital*: Jo Taylor, Laura Bough, Thomas Phillips, Barbara Winter- Goodwin; *Imperial College Healthcare NHS Trust*: Andrew Frankel, James Tomlinson, Marlon Alegata, Rashid Almasarwah, Anthoula Apostolidi, Maria Vourvou, Thomas Walters; *The Queen Elizabeth Hospital, King's Lynn*: Shiva Ugni, Smita Gunda, Rotimi Oluyombo, Vicki Brindle, Ping Coutts, Tracy Fuller, Evelyn Nadar; *University Hospital Aintree*: Christopher Wong, Christopher Goldsmith, Sherald Barnes, Ann Bennett, Claire Burston, Samantha Hope, Nicola Hunt, Lini Kurian; *UHNM Royal Stoke University Hospital*: Richard Fish, Daniela Farrugia, Judy Lee,

Emma Sadler, Hannah Turner; *Royal Devon and Exeter Hospital*: Helen Clarke, Victoria Carnall, Sarah Benyon, Caroline Blake, Stephanie Estcourt, Jane Piper; *Daisy Hill Hospital*: Neal Morgan, Carolyn Hutchinson, Teresa McKinley; *Kent & Canterbury Hospital*: Tim Doulton, Michael Delaney, Mahmoud Montasser, Jenny Hansen, David Loader, Angela Moon, Frances Morris; *University Hospital of Wales, Cardiff*: Donald Fraser, Mohammad Alhadj Ali, Sian Griffin, Farah Latif, Justyna Witczak, Alexa Wonnacott, Lynda Jeffers, Yvette Webley; *Ninewells Hospital*: Samira Bell, Leanne Cosgrove, Rachel Craik, Shona Murray; *Northern General Hospital, Sheffield*: Arif Khwaja, Yvonne Jackson, Angeline Mbuyisa, Rachel Sellars; *St. James's University Hospital, Leeds*: Andrew Lewington, Richard Baker, Suzannah Dorey, Kay Tobin, Rosalyn Wheatley; *Glasgow Clinical Research Facility, Queen Elizabeth University Hospital*: Rajan Patel, Patrick Mark, Alastair Rankin, Michael Sullivan, Kirsty Forsyth, Rowan McDougall

Supplemental Methods

Bioimpedance-derived parameters using the Body Composition Monitor (BCM)

The BCM computes extracellular and intracellular resistance measured in ohms. Application of a fluid model with these resistance values allows estimation of body water compartment volumes (extra- and intracellular water, ECW and ICW) and subsequently use of ECW and ICW in a three compartment body composition model with optimised tissue hydration parameters allows estimation of ECW excess (“Fluid Overload”) and estimations of lean and adipose tissue mass.[1] These parameters have been validated against gold standard techniques[2] and reproducibility has been demonstrated.[3,4] Unpublished but optimised tissue hydration parameters were provided by the device manufacturer Fresenius to EMPA-KIDNEY Bioimpedance Substudy collaborators (co-authors DK & DT) to allow derivation of these parameters based on previously published literature.[1]

Absolute “Fluid Overload” is the difference between expected (based on body weight and composition) versus measured ECW volume, reported in Litres. Relative “Fluid Overload” indexes the absolute “Fluid Overload” value to the measured ECW volume, and expressed as a percentage. Lean tissue mass in kilograms is indexed to height squared and expressed as lean tissue index (LTI) in kg/m^2. Estimates are also derived of adipose and fat tissue mass (ATM and FTM); adipose tissue mass consists of the fat tissue mass plus proteins, minerals and fluid. Fat tissue mass is indexed to height squared and expressed as fat tissue index (FTI) in kg/m^2.

Glossary of fluid-related terms

There is no standard nomenclature for bioimpedance-derived fluid overload parameters in existing literature, with a range of terminology and threshold values to infer clinical significance employed. We have used the following approach to report the EMPA-KIDNEY Bioimpedance Substudy.

Term used	Refers to
“Fluid Overload” (each word capitalised)	The bioimpedance-derived parameter
Fluid status (sentence case)	Description of the physiological state
Fluid excess (sentence case)	Description of the physiological state where it is necessary to describe individuals with fluid status in excess

Throughout the manuscript, “Fluid Overload” refers to the bioimpedance-derived parameters and fluid excess or fluid status is used to refer to the physiological state as appropriate.

Outcome definitions using the “Fluid Overload” parameter are further defined as follows:

**EMPA-KIDNEY Terminology**		**Definition**	**Equivalent Terminology (in other literature)**
Absolute “Fluid Overload”		A parameter reflecting excess extracellular water (ECW) (i.e. overhydration) in litres, computed as the difference between expected (based upon weight and body composition) versus measured ECW volume,[5] with positive values representing excess fluid<br>“Fluid Overload” = $ECW_{measured}$ - $ECW_{expected}$	- Overhydration[6]<br>- Hydration status[5] †<br>- Absolute tissue hydration[7]
Relative “Fluid Overload”		Overhydration index relative to measured ECW volume, expressed as a percentage[8]<br>Relative “Fluid Overload” = “Fluid Overload” ÷ $ECW_{measured}$	- Overhydration index[6,9] *<br>- Relative hydration status[8,10] †<br>- Relative tissue hydration[7]
Clinically Significant “Fluid Overload”	Moderate	Relative “Fluid Overload” >7%, ≤15% where 7% reflects the 90th percentile in a healthy reference population[7] and is approximately equivalent to absolute “Fluid Overload” of +1.1L[7]	
	Severe	Relative “Fluid Overload” >15% which represents the highest quartile in a haemodialysis reference population[5,8]; approximately equivalent to absolute “Fluid Overload” of +2.5L[7,8,11,12]	- Hyperhydration[8]

* Some scientific literature has used the term “overhydration index” to refer to both absolute “Fluid Overload” in litres and relative “Fluid Overload”.[9,13] We consider it to more accurately describe overhydration indexed to ECW.

† Hydration status expressed as ΔHS has also been used to refer to both absolute “Fluid Overload” in litres[5] as well as relative “Fluid Overload”[8,10].

The EMPA-KIDNEY definitions of moderate and severe clinically significant “Fluid Overload” are based upon existing literature which largely represents populations with advanced CKD requiring dialysis (because fluid excess is less common in earlier CKD). Wizemann *et al*. established a 15% threshold value of relative “Fluid Overload” (referred to as relative hydration status) based upon the highest quartile of a reference haemodialysis population.[8] This threshold is approximately equivalent to >+2.5L absolute “Fluid Overload” in patients on hemodialysis,[5,8,14] and is strongly associated with mortality.[8,9,11,12,15-19] In EMPA-KIDNEY, the threshold of >15% relative “Fluid Overload” is referred to as “severe” as the study population can be expected to exhibit lower levels of fluid overload than dialysis populations. The moderate “Fluid Overload” threshold of ≥7% has also been associated with risk of death in dialysis cohorts,[12,19-21] and more recently, in those with earlier stages of CKD.[10,22,23] A scoping review was conducted assessing evidence for various other thresholds reported in existing literature before confirming the specified outcome definitions as the most appropriate values. Note that, although the majority of existing data on the use of the bioimpedance in CKD arises from dialysis populations, in a systematic review we identified 11 non-dialysis CKD cohorts comprising almost 7000 participants reporting associations of adverse outcomes with bioimpedance-derived fluid parameters. The BCM device was used in 7 of these studies (>1500 participants in total).[22]

Weighting of the analyses in the bioimpedance substudy

Weighting of values obtained corresponding to the 2- and 18-month follow-up visits were weighted according to the relative duration of each follow-up time period. The ideal time point for a 2-month measurement was 60 days post-randomization but measurements taken on or after day 30 but before day 400 were mapped to the 2-month visit. The 18-month visit ideally occurred on day 540 post-randomization and readings on or after day 400 and before day 680 could be analysed in reference to this time point. The first follow-up window is therefore 370 days in duration (≥30 to <400 days) and the second spanning 280 days (≥400 to <680 days) therefore analyses were pre-specified to weight information from the first time period at approximately 55% compared to 45% for the second time period (weighting factors of 0.569 and 0.431). This was considered appropriate based on a hypothesised larger effect on “Fluid Overload” at the early 2-month time period relative 18 months based on known haemodynamic mechanisms of the intervention.

Handling of missing and duplicate data

In bioimpedance substudy analyses, participants with a missing baseline bioimpedance measurement could still be included if subsequent bioimpedance measurements were obtained within the 2- and/or 18-month follow-up windows. Missing baseline bioimpedance measurements were imputed with the mean observed value across both treatment groups combined. Participants with missing baseline values relevant to subgroup analyses were included in the subgroup containing the mean value (or the most frequent category for a binary variable). Missing follow-up bioimpedance measurements including “Fluid Overload” at 2 and 18 months were handled by the mixed model repeated measures (MMRM) modelling approach.

In all bioimpedance substudy analyses, if more than one valid bioimpedance measurement was available at a single follow-up visit (i.e. date), the measurement with the highest Q value (see Data Analysis Plan appendix 8.1) was used and additional measurements ignored. In all bioimpedance substudy analyses, if valid bioimpedance measurements are made on more than one day within a follow-up period, then the valid bioimpedance measurement made on the day nearest the ideal follow-up day was used.

In analyses of the full trial cohort, participants missing baseline measurements for weight, blood pressure, HbA1c or hematocrit were excluded from the analysis, following the procedure pre-specified in the main EMPA-KIDNEY trial analyses for consistency with previously published results. Analyses of waist and hip circumference and waist-to-hip ratio required a different analytic approach since measurements were only made at a single time-point. All 6609 participants were included, missing baseline measurements were handled by mean imputation and missing follow-up measurements were handled by multiple imputation. The multiple imputation model included non-missing values of baseline and follow-up measurements of waist, hip, waist-to-hip ratio, weight and BMI, as well as the minimization algorithm variables (age, sex, eGFR, uACR and region) and NT-proBNP (which was associated with missingness of waist-to-hip ratio univariable logistic regression). Imputation was carried out separately by treatment allocation. Multiple imputation produced 20 imputed datasets which were each analysed by ANCOVA and treatment-specific estimated marginal means and standard errors were then pooled using the method of Rubin. All multiple imputation analyses were implemented using the multiple imputation procedure in SAS version 9.4 (SAS Institute, Cary NC), using the expectation-maximization algorithm (which assumes a multivariate normal distribution) to impute values.

Data quality assessment pilot procedure in the bioimpedance substudy

Data quality assessment was devised and tested blind to treatment allocation using a preliminary dataset in April 2022. The data quality assessment process was then applied to the complete dataset in November 2022, while reviewers still remained blinded to individual participants' treatment allocation. This was completed independently by two reviewers, with differences resolved by discussion. The main results of the EMPA-KIDNEY trial were published on 4th November 2022 therefore reviewers were inevitably unblinded to the main trial results.

Two levels of assessment were devised with the support of a clinical scientist experienced in the use and interpretation of bioimpedance data:

(A) Criteria to be applied to all bioimpedance readings to identify readings which may be of poor quality and should be further assessed by visual inspection of Cole-Cole plots (see Data Analysis Plan Appendix 8.1)

(B) Criteria to be applied when visually inspecting Cole-Cole plots for readings identified in step (A) to determine inclusion in the primary analysis (see Data Analysis Plan Appendix 8.4)

From the preliminary dataset containing 1495 readings, 172 readings were identified as meeting at least one of the above criteria (A) triggering visual inspection of the Cole-Cole plot (B). Reviewer 1 was trained by expert reviewer 2 in assessment of the Cole-Cole plot. There are no published guidelines for assessment of the Cole-Cole plot therefore these were devised based upon expert knowledge of reviewer 2 (see Data Analysis Plan Appendix 8.4) and revised in an iterative process throughout three rounds of Cole-Cole plot review. Reviewers then agreed upon one key criterion (B) (see Data Analysis Plan Appendix 8.4) to be applied when assessing Cole-Cole plots for measurements in the final dataset to determine inclusion in the primary analysis. This criterion was applied to all identified measurements by both reviewers independently with differences resolved by discussion.

This pilot process also informed revision of criteria (A) which were being used to identify measurements requiring Cole-Cole plot review as the original criteria were thought to identify measurements for review unnecessarily, potentially introducing bias. The revised criteria (see table) aimed to minimise this.

Data quality assessment: Original and revised criteria (A) for identification of bioimpedance measurements to be further assessed by Cole-Cole plot review

Original criterion	Revised criterion	Justification
Exclusion* if Q value <80	Q value <80 triggered manual review of Cole-Cole plot in each case	All Cole-Cole plots with a Q value <80 were reviewed and >50% deemed of acceptable quality based on Cole-Cole plot review therefore this revised criterion is necessary to avoid automatic exclusion of valid readings based on Q<80 alone.<br>A random subset of 50 measurements with a Q score ≥80 were also selected for Cole-Cole plot review to test this criterion. Q scores above this threshold were confirmed to be a reliable indicator of good data quality.
Exclusion* if absolute “Fluid Overload” extreme outlier: >2 standard deviations (SD) from the mean	Absolute “Fluid Overload” more negative than -5L automatically excluded.<br>All other outliers retained and do not warrant Cole-Cole plot review.	Cole-Cole plots for all measurements lying >2 SD from the mean were reviewed. It was found that outlying positive values of “Fluid Overload” were reliably associated with satisfactory Cole-Cole plots whereas outlying negative values were consistently associated with poor Cole-Cole plots. The -5L cut-off was selected based on review of each individual plot combined with clinical reasoning based upon plausibility.
Exclusion* if multiple measurements at the same time with more than 0.5L between the highest and lowest absolute “Fluid Overload” values	If more than one valid bioimpedance measurement is available at a single follow-up visit (i.e. date), the measurement with the highest Q value will be used and additional measurements ignored.	All duplicate measurements were reviewed in the pilot dataset and the measurement with the highest Q score also had the most favourable Cole-Cole plot in every case. MMRM analysis requires a single reading for each participant at each time point.

* exclusion from primary outcome assessment

Tertiary & exploratory *post-hoc* assessments in the bioimpedance substudy

Tertiary assessments included effects of empagliflozin on the primary outcome by four pre-specified substudy subgroups: sex, diabetes status, N-terminal pro-brain-type natriuretic peptide (NT-proBNP) and eGFR at randomization. Post-hoc exploratory subgroup analyses by baseline hydration status, any diuretic use, and race were also performed. Baseline "Fluid Overload" was subcategorized into fluid depletion; normohydration; moderate "Fluid Overload"; and severe "Fluid Overload", based on the following relative "Fluid Overload" cut-offs: ≤ -7%; > -7%, ≤ +7%; > +7%, ≤ +15%; and > +15%. The normohydration category – which included 60% of the substudy population – was further separated into low- (> -7% ≤ 0%) and high-normohydration (> 0% ≤ +7%). Other tertiary assessments included effects of empagliflozin versus placebo on ECW, ICW, LTI, FTI, body weight, body mass index (BMI), waist and hip circumference and their ratio; and effects on each of the four components of the key secondary outcome; and time-to-first outcome of regression of "Fluid Overload" (i.e. regression of moderate or severe "Fluid Overload" at randomization to any lower hydration category).

Clinical assessments in the full trial cohort

Analyses include effects on weight, body mass index, waist-to-hip ratio and blood pressure in the full EMPA-KIDNEY trial cohort and exclude participants with missing baseline values of the outcome variable of interest in each analysis. These measurements were made at routine trial visits using Local Clinical Centre (LCC) equipment (i.e. standardised equipment and techniques were not mandated and calibration certificates were not required). This approach was adopted in accordance with the trial's streamlined design, aiming to provide generalizable results using "real-world" measurements. Weight (kg) and blood pressure (mmHg) were measured at the randomization visit and all subsequent scheduled visits. Height (metres) was measured at randomization and used to calculate BMI as weight divided by height squared for each study visit. Waist (i.e. the smallest part of the trunk or the level of the umbilicus if natural indent not apparent) and hip (the widest area around the hips) circumferences were measured in centimetres at randomization, 18 months and the final visit only. Weight, height and waist/hip circumferences were required to be measured in the specified units – no conversion from imperial units was permitted. Trained LCC Research Co-ordinators (LRC) were advised to obtain measurements without footwear, outer clothing and with items removed from pockets. Guidance was provided to measure waist circumference in the standing position during exhalation, with arms folded. Blood pressure was measured using an automated digital

sphygmomanometer or manual device if more appropriate (e.g. if the participant had an irregular heart beat) using an appropriately sized cuff. LRCs were advised the participant should sit comfortably for five minutes prior; to apply the cuff to the exposed upper arm at the level of the heart; neither the LRC nor the participant should speak during measurement and the participant should be advised to remain still. Only one reading was required, in accordance with streamlined trial principles.

Laboratory assessments in the full trial cohort

Analyses include effects on glycated hemoglobin (HbA1c) and hematocrit in the full EMPA-KIDNEY trial cohort. HbA1c was measured in the central laboratory at randomization, 2 and 18 months and the final follow-up visit (varies by participant); measurements at 2-, 18- 24- and 30-month time points were included in analyses of the full trial cohort. Kit boxes were provided by the Central Co-ordinating Centre to be used to collect and store the samples required for central analysis. Guidance was provided on centrifugation and storage prior to transfer to the central laboratory. HbA1c determination used the high-performance liquid chromatographic (HPLC) method using ethylenediaminetetraacetic acid (EDTA) blood on an Arkray HA8180 analyser and reagents with a calibrator supplied by Menarini Diagnostics UK traceable to International Federation of Clinical Chemistry (IFCC) reference standards. Hematocrit was assessed in an approximately 20% subset of the full trial cohort using local laboratory measurements at randomization and 18 months only. Sample collection bottles/tubes for local laboratory testing were not supplied by the study, so used the bottles which are sourced locally for routine clinical use. LRCs were instructed to enter all test results from the local laboratory into the electronic care report form within 48 hours of collection and were requested to keep a paper copy of any tests results provided by the local laboratory specifically for the study within the participant's study records for monitoring purposes.

Supplemental methods references

1. Chamney PW, Wabel P, Moissl UM, et al. A whole-body model to distinguish excess fluid from the hydration of major body tissues. *Am J Clin Nutr* 2007; **85**(1): 80-9.
2. Wabel P, Chamney P, Moissl U, Jirka T. Importance of whole-body bioimpedance spectroscopy for the management of fluid balance. *Blood Purif* 2009; **27**(1): 75-80.
3. Wabel P CP, Moissl U et al. Reproducibility of bioimpedance spectroscopy (BIS) for

the assessment of body composition and dry weight. . 2007: J Am Soc Nephrol 2007. p. 255.
4. Hannan WJ, Cowen SJ, Plester CE, Fearon KC, deBeau A. Comparison of bio-impedance spectroscopy and multi-frequency bio-impedance analysis for the assessment of extracellular and total body water in surgical patients. *Clin Sci (Lond)* 1995; **89**(6): 651-8.
5. Wabel P, Moissl U, Chamney P, et al. Towards improved cardiovascular management: the necessity of combining blood pressure and fluid overload. *Nephrol Dial Transplant* 2008; **23**(9): 2965-71.
6. Wang Y, Gu Z. Effect of bioimpedance-defined overhydration parameters on mortality and cardiovascular events in patients undergoing dialysis: a systematic review and meta-analysis. *J Int Med Res* 2021; **49**(9): 3000605211031063.
7. Van Biesen W, Williams JD, Covic AC, et al. Fluid status in peritoneal dialysis patients: the European Body Composition Monitoring (EuroBCM) study cohort. *PLoS One* 2011; **6**(2): e17148.
8. Wizemann V, Wabel P, Chamney P, et al. The mortality risk of overhydration in haemodialysis patients. *Nephrol Dial Transplant* 2009; **24**(5): 1574-9.
9. Tabinor M, Elphick E, Dudson M, Kwok CS, Lambie M, Davies SJ. Bioimpedance-defined overhydration predicts survival in end stage kidney failure (ESKF): systematic review and subgroup meta-analysis. *Sci Rep* 2018; **8**(1): 4441.
10. Tsai YC, Chiu YW, Tsai JC, et al. Association of fluid overload with cardiovascular morbidity and all-cause mortality in stages 4 and 5 CKD. *Clin J Am Soc Nephrol* 2015; **10**(1): 39-46.
11. Zoccali C, Moissl U, Chazot C, et al. Chronic Fluid Overload and Mortality in ESRD. *J Am Soc Nephrol* 2017; **28**(8): 2491-7.
12. Dekker MJ, Marcelli D, Canaud BJ, et al. Impact of fluid status and inflammation and their interaction on survival: a study in an international hemodialysis patient cohort. *Kidney Int* 2017; **91**(5): 1214-23.
13. O'Lone EL, Visser A, Finney H, Fan SL. Clinical significance of multi-frequency bioimpedance spectroscopy in peritoneal dialysis patients: independent predictor of patient survival. *Nephrol Dial Transplant* 2014; **29**(7): 1430-7.
14. Wieskotten S, Heinke S, Wabel P, et al. Bioimpedance-based identification of malnutrition using fuzzy logic. *Physiol Meas* 2008; **29**(5): 639-54.
15. Caetano C, Valente A, Oliveira T, Garagarza C. Body Composition and Mortality Predictors in Hemodialysis Patients. *J Ren Nutr* 2016; **26**(2): 81-6.
16. Chazot C, Wabel P, Chamney P, Moissl U, Wieskotten S, Wizemann V. Importance of normohydration for the long-term survival of haemodialysis patients. *Nephrol Dial Transplant* 2012; **27**(6): 2404-10.
17. Kim YJ, Jeon HJ, Kim YH, et al. Overhydration measured by bioimpedance analysis and the survival of patients on maintenance hemodialysis: a single-center study. *Kidney Res Clin Pract* 2015; **34**(4): 212-8.
18. Onofriescu M, Siriopol D, Voroneanu L, et al. Overhydration, Cardiac Function and Survival in Hemodialysis Patients. *PLoS One* 2015; **10**(8): e0135691.
19. Siriopol D, Siriopol M, Stuard S, et al. An analysis of the impact of fluid overload and fluid depletion for all-cause and cardiovascular mortality. *Nephrol Dial Transplant* 2019; **34**(8): 1385-93.
20. Siriopol I, Siriopol D, Voroneanu L, Covic A. Predictive abilities of baseline measurements of fluid overload, assessed by bioimpedance spectroscopy and serum N-terminal pro-B-type natriuretic peptide, for mortality in hemodialysis patients. *Arch Med Sci* 2017; **13**(5): 1121-9.
21. Siriopol D, Voroneanu L, Hogas S, et al. Bioimpedance analysis versus lung ultrasonography for optimal risk prediction in hemodialysis patients. *Int J Cardiovasc Imaging* 2016; **32**(2): 263-70.
22. Mayne KJ, Shemilt R, Keane DF, Lees JS, Mark PB, Herrington WG. Bioimpedance indices of fluid overload and cardiorenal outcomes in heart failure and chronic kidney disease: a systematic review. *J Card Fail* 2022.
23. Hung SC, Lai YS, Kuo KL, Tarng DC. Volume overload and adverse outcomes in chronic kidney disease: clinical observational and animal studies. *J Am Heart Assoc* 2015; **4**(5).

EMPA-KIDNEY Body Composition Measurement Substudy

Study Title: A multicentre international randomized parallel group double-blind placebo-controlled clinical trial of EMPAgliflozin once daily to assess cardio-renal outcomes in patients with chronic KIDNEY disease

Sponsor protocol number: 1245-0137

Protocol identifier: CTSUEMPA-KIDNEY1.4 2018-04-25

EudraCT number: 2017-002971-24

Summary

This document provides the rationale and design of an EMPA-KIDNEY substudy to measure body composition in a subset of the 5000 EMPA-KIDNEY participants using bioimpedenace spectroscopy. The substudy does not alter the main protocol in any respect.

Background

In the EMPA-REG OUTCOME trial, empagliflozin 10-25mg was shown to reduce the composite outcome of death from cardiovascular causes, nonfatal myocardial infarction, or nonfatal stroke by 14% compared to placebo (hazard ratio [HR] 0.86, 0.74-0.99) in 7020 people with type 2 diabetes mellitus (T2DM) and prior atherosclerotic cardiovascular disease.[1] This effect was in large part the result of a highly significant 38% (HR 0.62, 0.49-0.77) reduction in cardiovascular death. The pre-specified secondary outcome of hospitalization for heart failure was reduced by 35% (HR 0.65, 0.50-0.85).[1] Exploration of EMPA-REG OUTCOME data has suggested that the increase in haematocrit caused by empagliflozin, a possible surrogate for reductions in plasma volume, was the intermediate clinical parameter with the largest mediating effect on the reduction in cardiovascular death.[2] These observations may have particular relevance in people with chronic kidney disease (CKD) who have disturbed salt and water homeostasis which may cause chronic fluid overload which in turn contributes to the observed excess of structural heart disease and heart failure.[3]

In EMPA-REG OUTCOME, allocation to empagliflozin led to a sustained loss of weight (of about 2Kg from a mean of 86Kg) and a 2cm reduction in waist circumference (from a mean of 105cm).[1] How much of this weight change reflected reduction in total body water versus adipose tissue is unknown. A previous trial suggested that, after 2 years, weight loss resulting from SGLT-2 inhibition in people with T2DM appears almost completely attributable to reduced adipose tissue (measured using dual energy X-ray absorptiometry).[5] Lower kidney function substantially reduces glycosuric effects of SGLT-2 inhibition, and so reduced calorie loss at lower levels of kidney function may attenuate any loss of adipose tissue. However, no attenuation of the weight-lowering effects of SGLT-2 inhibition

was identified in those with CKD compared to those without (within the range of kidney function studied to date).[6-8] Furthermore, meta-analysis of three large placebo-controlled trials suggests effects of SGLT-2 inhibition on heart failure are at least as large among those with reduced kidney function.[9] Part of the preserved effect of SGLT-2 inhibition on body weight and heart failure in CKD may therefore result from reductions in excess extracellular water (ECW) being preserved in those with CKD, despite attenuated effects on glycosuria. This raises a hypothesis that the effects of empagliflozin on excess ECW and fat levels may be different in people with different levels of kidney function.

Figure 1: Effect of SGLT-2 inhibition versus placebo on hospitalization for heart failure, by baseline kidney function (meta-analysis EMPA-REG OUTCOME, CANVAS and DECLARE) [9]

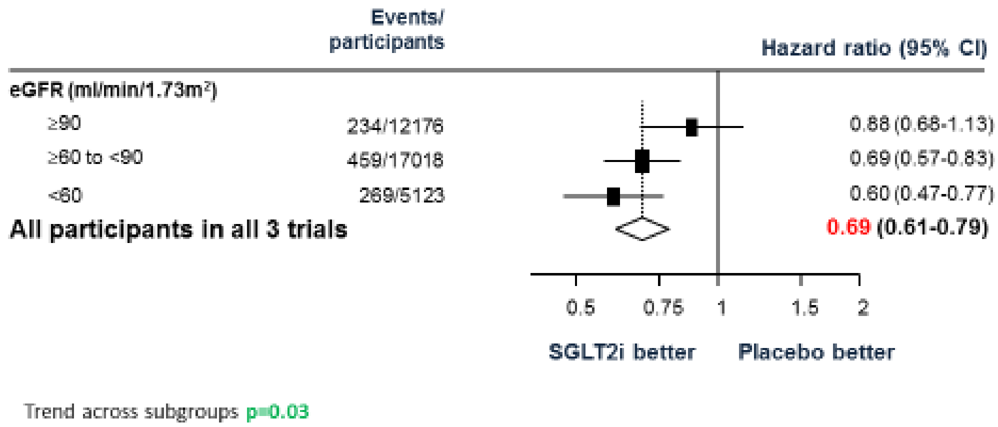

Bioimpedance spectroscopy can assess different resistance patterns in the body which are affected by the amount of water present. Low frequency current exclusively passes extracellularly, whereas high frequencies can pass through all body water compartments. Comparing spectroscopy readings over a range of frequencies it is possible to derive total body water in Litres and separately the volume of ECW. From such measurements it is also possible to estimate normally hydrated adipose tissue and lean tissue mass, from which an index referred to as "Fluid Overload" (or overhydration) can be algorithmically calculated.[10] Sustained "Fluid Overload" measured by bioimpedance spectroscopy has been associated with increased risk of mortality among people on dialysis,[4] and some dialysis units are now using bioimpedance spectroscopy measurements clinically to guide patients' fluid management and dialysis prescription.

At each Follow-up Visit, EMPA-KIDNEY participants will have their weight measured and central plasma/serum blood samples collected. At Randomization, 2 & 18 months and Final Follow-up Visit, they will also have a measure of waist and hip circumference. A substudy using bioimpedance-based body composition measurements will ensure uncertainty about the effects of empagliflozin on ECW, adipose tissue and particularly "Fluid Overload" will be assessed in a CKD population.

Aims

The primary aim of this substudy is to use bioimpedance spectroscopy to assess, in a subset of EMPA-KIDNEY participants, the effect of empagliflozin 10mg versus matching placebo on "Fluid Overload" at the 2 month and 18 month Follow-up Visits.

Secondary aims are to use bioimpedance spectroscopy to assess:

1. Whether any effects of empagliflozin 10mg versus matching placebo on "Fluid Overload" are modified by baseline factors, in particular by level of kidney function, glycosylated haemoglobin, body mass index, NT-proBNP, age, sex, RAS inhibitor use, and different diuretics
2. The effects of empagliflozin 10mg versus matching placebo early and later during follow-up on:
    - ECW
    - Intracellular water (ICW)
    - Adipose tissue mass indexed to weight (i.e. %)
    - Lean tissue mass indexed to weight (i.e. %)

Exploratory aims are to:

- Assess if changes in ECW, ICW, % adipose tissue mass, % lean tissue mass and "Fluid Overload" correlate with changes in blood pressure and relevant other biomarkers.

Sample size estimates

The study will start a vanguard phase in a small number of sites in which bioimpedance spectroscopy will be performed at Randomization, 2 months and 18 months of Follow-up Visits. This vanguard phase will be expanded to other sites once feasiblity of adding a bioimpedance spectroscopy measurement is demonstrated. Feasbility will be based on feedback from the participating sites, successful completion of the other protocol-specified procedures and logistical considerations. It is estimated that at least 400 (of the 5000) EMPA-KIDNEY participants with follow-up bioimpedance spectroscopy measurements will provide ample power (>90%, 2p=0.05) to detect at least a ±300mL difference in "Fluid Overload" (reference range in healthy adults is ± 1100mL with a standard deviation of 900mL) based on an independent 2-sided t-test (Table 1).

Table 1: Sample size calculations for a study with Randomization Visit measurements

Outcome	Effect size	Standard deviation	Required sample size
"Fluid Overload" (ref range: ±1.1L)	±300mL	900mL	382

Note: An estimate of the correlation between successive bioimpedance spectroscopy measurements would be required to calculate the reduction in sample size that could be achieved by using ANCOVA analyses, but no such longitudinal data has yet been collected in a CKD population.

If a bioimpedance spectroscopy measurement at the Randomization Visit is shown to be infeasible, the substudy will be modifed to exclude the measurement at the Randomization Visit and only be performed at the relatively less busy phases of the study (i.e. measurements will be restricted to the 2 and 18 month Follow-up Visits). In this design, the sample size would need to increase to 850. This is because the absence of a bioimpedance spectroscopy measurement at the Randomization Visit means any imbalances in "Fluid Overload" between treatment arms at baseline cannot be corrected for. These imbalances could result in either the treatment effect being overestimated or a smaller than expected difference in mean "Fluid Overload" at follow-up. However, with a sample size of 850, the probability of large baseline imbalances is small, making it unlikely that the treatment effect would be overstated by more than 100mL (Table 2). With a sample size of 850, there would be sufficient power to detect a reduced difference in mean "Fluid Overload" of ± 200 mL at follow-up. This calculation is based on an independent 2-sided t-test using data from a healthy population (Note: sample size estimates differ little if dialysis population data are used).

Table 2: Sample size calculations for a study without Randomization Visit measurements

Sample size	Assumed possible baseline imbalance in Fluid Overload (mL)	Probability of a baseline imbalance at least this size due to chance (1-sided)	Difference between groups at follow-up (mL) after subtracting possible baseline imbalance from assumed treatment effect of 300 mL	Power to detect reduced difference in follow-up values at 2p=0.05
850	0	Not applicable	300	>99%
	50	12.6%	250	98%
	100	1.1%	200	90%
	150	0.03%	150	68%
	200	0.0002%	100	37%

Data Analysis Plan

The primary analysis will estimate the differences in "Fluid Overload" between treatment groups across all time points, regardless of whether a participant received all, some or none of their allocated treatment (i.e. intention-to-treat analyses). Secondary outcomes include ECW, ICW, % adipose tissue mass, and % lean tissue mass“. Differences in "Fluid Overload” and the secondary outcomes between treatment groups overall, and separately at 2 and 18 months, will be calculated using linear regression adjusted (or stratified) for the elements included in the minimization algorithm. The primary analysis will focus on a weighted average of the values at the two time points (with weights proportional to the amount of time between visits). Missing measurements will be imputed. Results from the imputed analyses will be compared with those from equivalent “complete-case” analyses, but primary emphasis will be placed on the results after multiple imputation. More complete details of statistical methods, including definitions of subgroups, methods of imputation, approaches to adjustments and

weighting of averages will be set out in a separate full Data Analysis Plan which will be consistent with the main study Data Analysis Plan.

Flowchart of Substudy Activities

**INVITATION**
• Invite potential participants shortly before or at the time of the Randomization Visit<br>• Written informed consent is sought from willing individuals at the first visit when a bioimpedance spectroscopy measurement is offered
**RANDOMIZATION VISIT AND AT 2 & 18 MONTHS OF FOLLOW-UP**
• A bioimpedance spectroscopy measurement is added to the protocol-specified study follow-up visit procedures

Design

Eligibility: In selected regions, EMPA-KIDNEY Local Clinical Centres (LCCs) with a Fresenius Medical Care Body Composition Monitor (BCM) machine will be invited to join this optional substudy. All those participants at these LCCs who have yet to attend the relevant scheduled study visit are eligible for invitation. There are no exclusion criteria.

Invitation and methods: Potential participants will be invited to join this substudy at before or around the time of their Randomization Visit. At the relevant visit, clinic staff will explain the substudy to potential participants using the Participant Information Leaflet and Consent Form. Consenting participants will have a measure of bioimpedance made in addition to the protocol-specified follow-up procedures. Bioimpedance measurements take about 2 minutes to record and pose no risk to health (although it is conceivable the 4 self-adhesive pads could rarely cause a skin reaction).

Body Composition Measurement

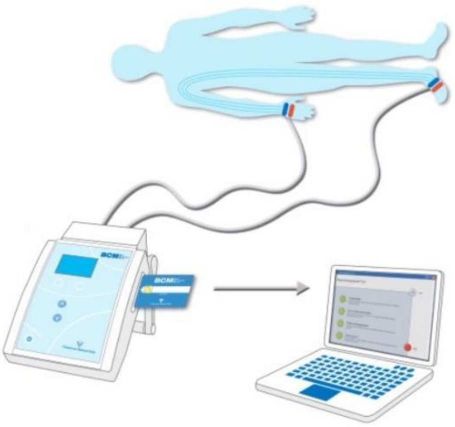

Training materials on how to perform Body Composition Measurements will be provided. The measurement requires four disposable self-adhesive electrode pads (2x on a wrist and the other 2x on an ankle) to be attached to a portable machine whilst a participant is lying supine. Bioimpedance spectroscopy readings are made automatically across about 50 frequencies over a range of 5-1000 kHz. The measurements take about 2 minutes to make. Data are then automatically transferrable onto a Storage Card which is linked securely to the participant by means of a unique Storage Card ID entered onto the relevant study visit form on trial's web-based data entry system (i.e. Storage Cards containing results are pseudonymised). The Storage Card will be stored securely before being transferred securely to the Central Coordinating Office in Oxford for downloading into the study database. Data may be transferred securely to specialists in bioimpedance for Quality Control review.

References

1. Zinman B, Wanner C, Lachin JM, et al. Empagliflozin, Cardiovascular Outcomes, and Mortality in Type 2 Diabetes. *N Engl J Med.* 2015;373(22):2117-2128.
2. Inzucchi SE, Zinman B, Fitchett D, et al. How Does Empagliflozin Reduce Cardiovascular Mortality? Insights From a Mediation Analysis of the EMPA-REG OUTCOME Trial. *Diabetes Care.* 2018;41(2):356-363.
3. Bui AL, Horwich TB, Fonarow GC. Epidemiology and risk profile of heart failure. *Nat Rev Cardiol.* 2011;8(1):30-41.
4. Zoccali C, Moissl U, Chazot C, et al. Chronic Fluid Overload and Mortality in ESRD. *J Am Soc Nephrol.* 2017;28(8):2491-2497.
5. Ridderstrale M, Andersen KR, Zeller C, et al. Comparison of empagliflozin and glimepiride as add-on to metformin in patients with type 2 diabetes: a 104-week randomised, active-controlled, double-blind, phase 3 trial. *Lancet Diabetes Endocrinol.* 2014;2(9):691-700.
6. Petrykiv S, Sjostrom CD, Greasley PJ, Xu J, Persson F, Heerspink HJL. Differential Effects of Dapagliflozin on Cardiovascular Risk Factors at Varying Degrees of Renal Function. *Clin J Am Soc Nephrol.* 2017;12(5):751-759.
7. Kohan DE, Fioretto P, Tang W, List JF. Long-term study of patients with type 2 diabetes and moderate renal impairment shows that dapagliflozin reduces weight and blood pressure but does not improve glycemic control. *Kidney Int.* 2014;85(4):962-971.
8. Cherney DZI, Cooper ME, Tikkanen I, et al. Pooled analysis of Phase III trials indicate contrasting influences of renal function on blood pressure, body weight, and HbA1c reductions with empagliflozin. *Kidney Int.* 2018;93(1):231-244.
9. Zelniker TA, Wiviott SD, Raz I, et al. SGLT2 inhibitors for primary and secondary prevention of cardiovascular and renal outcomes in type 2 diabetes: a systematic review and meta-analysis of cardiovascular outcome trials. *Lancet.* 2019;393(10166):31-39.
10. Wabel P, Chamney P, Moissl U, Jirka T. Importance of whole-body bioimpedance spectroscopy for the management of fluid balance. *Blood Purif.* 2009;27(1):75-80.
11. Moissl U, Arias-Guillen M, Wabel P, et al. Bioimpedance-guided fluid management in hemodialysis patients. *Clin J Am Soc Nephrol.* 2013;8(9):1575-1582.

EMPA-KIDNEY Body Composition Monitor Substudy Data Analysis Plan (EDMS 7635)

Version History

Version	Description	Authors
1.0	First released version	Created by: Kaitlin J. Mayne, Natalie Staplin, Richard Haynes & William G. Herrington (JAN-MAR2022)<br>Reviewed by Rebecca Sardell, Will Stevens, Karl Wallendszus, Jonathan Emberson, John Nolan, Dani Trinca, David Keane, Simon Davies, Rejive Dayanandan, Akiko Omata, Parminder Judge, Ryonfa Lee, Patrick Mark, Jennifer Lees, Vladimir Cejka, Christoph Wanner (MAR2022) & Colin Baigent (APR2022)<br>Review by Steering Committee (18MAY2022)<br>Released for use by William G. Herrington (08JUN2022)
1.1	Revision to Appendix 8.1: Definition of a valid BCM measurement<br>Addition of Appendices 8.4 and 8.5.	Revised by: Kaitlin J. Mayne, David Keane, & William G, Herrington (18OCT-16NOV2022)<br>Released for use by William G. Herrington (17NOV2022)

This is a controlled document. Distribution and approval is to be managed using the Electronic Document Management System.

TABLE OF CONTENTS

Contents

1 RELEVANT PROCEDURAL DOCUMENTS 3
2 ABBREVIATIONS 3
3 INTRODUCTION 4
4 KEY FLUID OVERLOAD DEFINITIONS 5
5 BASELINE CHARACTERISTICS 6
6 DEFINITIONS OF KEY RANDOMIZED ASSESSMENTS 8
6.1 Hypotheses 8
6.2 Primary randomized assessment 8
6.3 Key secondary randomized assessment 9
6.4 Other secondary randomized assessment 9
6.5 Tertiary randomized assessments including subgroup analyses 9
6.6 Additional exploratory analyses 10
7 STATISTICAL METHODOLOGY 11
7.1 Handling of missing and extreme values 11
7.2 Methods of analysis 11
7.2.1 Primary randomized assessment 11
7.2.2 Assessment for key secondary randomized assessment 12
7.2.3 Other secondary randomized assessment 12
7.2.4 Tertiary randomized assessments including subgroup analyses 12
8 APPENDIX: DEFINITION OF VALID BCM MEASUREMENTS AND DATA HANDLING CONSIDERATIONS 13
8.1 Definition of a valid BCM measurement 13
8.2 Handling multiple BCM measurements 14
8.2.1 Multiple valid BCM measurements at the same visit 14
8.2.2 Multiple valid BCM measurements within a Follow-up window 14
8.2.3 Multiple measurements at different visits on a single BCM card 14
8.3 Data processing: BCM variables 15
8.4 Criteria for rejecting BCM measurements by Cole-Cole plot visual inspection 16
8.5 Sensitivity analyses 17
REFERENCES 18

1 RELEVANT PROCEDURAL DOCUMENTS

Document title	EDMS#
EMPA-KIDNEY Protocol	5434
EMPA-KIDNEY BCM Substudy Protocol Supplement	6251
EMPA-KIDNEY Data Analysis Plan (SOP11)	6290
EMPA-KIDNEY BCM datacard download IOP	6433
EMPA-KIDNEY Leeds BCM Card Data Transfer for Outcome Derivation	7248
EMPA-KIDNEY BCM kit leaflet	6240

2 ABBREVIATIONS

Abbreviation	Definition
ACR	Albumin-to-creatinine ratio
ATM	Adipose tissue mass
BCM	Body composition monitor
BMI	Body mass index
CKD-EPI	Chronic Kidney Disease Epidemiology Collaboration
DPP-4	Dipeptidyl peptidase-4
ECW	Extracellular water
EDMS	Electronic document management system
eGFR	Estimated glomerular filtration rate
FTI	Fat tissue index
GLP-1	Glucagon-like peptide-1
HbA1c	Glycosylated haemoglobin
ICW	Intracellular water
LTI	Lean tissue index
LTM	Lean tissue mass
MMRM	Mixed model repeated measures
NT-proBNP	N-terminus prohormone of brain natriuretic peptide
RAS	Renin-angiotensin system
SOP	Standard operating procedure
TBW	Total body water

3 INTRODUCTION

This document provides a Data Analysis Plan for the EMPA-KIDNEY substudy, which has measured body composition of a subset of approximately 650 EMPA-KIDNEY participants recruited from the UK and Germany using bioimpedenace spectroscopy on a body composition monitor (BCM). An outline BCM data analysis plan was provided in the BCM substudy's Protocol Supplement (EDMS#6251). The purpose of this BCM Data Analysis Plan is to define, before unblinding of the treatment allocation, detail of pre-specified randomized analyses to be presented in initial publication(s) of the substudy. The nature of all analyses (randomized or observational) including those related to subsequent publications and exploratory analyses cannot be specified in detail but, where appropriate, a general analytical approach is set out. Approaches, wherever possible, will follow those set out in EMPA-KIDNEY's main data analysis plan (SOP11; EDMS#6290).

Note: this pre-specified Data Analysis Plan re-orders the priority of some of the assessments set out in the BCM substudy Protocol Supplement (EDMS#6251). Certain assessments have been moved from secondary to tertiary assessments, and a new key secondary assessment introduced. This follows a more detailed review of data whilst compiling this plan. This pre-specified Data Analysis Plan therefore supersedes the proposed assessments set out in the Protocol Supplement and prevails in the event of any discrepancies between the two documents. In addition to the pre-specified comparisons, other post-hoc analyses may be performed with due allowance for their exploratory and, perhaps, data-dependent nature.

4 KEY FLUID OVERLOAD DEFINITIONS

There is no standard nomenclature for BCM-derived fluid overload parameters in existing literature, with a range of terminology and threshold values to infer clinical significance employed. We have used the following approach to report the EMPA-KIDNEY BCM substudy.

**Terminology**		**Definition**
Fluid Overload		Overhydration in litres, computed as the difference between expected (based upon weight and body composition) versus measured extracellular water (ECW) volume (1), with positive values representing excess fluid.<br>Fluid Overload = $ECW_{measured}$ - $ECW_{expected}$.
Relative Fluid Overload		Overhydration index* relative to measured ECW volume, expressed as a percentage (2).<br>Relative Fluid Overload = Fluid Overload ÷ $ECW_{measured}$.
Clinically Significant Fluid Overload	Moderate	Relative Fluid Overload >7% to ≤15% [where 7% reflects the 90th percentile in a healthy reference population and is approximately equivalent to absolute Fluid Overload of +1.1L (3)].
	Severe	Relative Fluid Overload >15% [which represents the highest quartile in a haemodialysis reference population (1, 2); approximately equivalent to absolute Fluid Overload of +2.5L (2-5)].

Note: In the original table, "Clinically Significant Fluid Overload" spans both the Moderate and Severe rows.

Footnote: **Although scientific literature has used the term "overhydration index" to refer to both absolute Fluid Overload in litres and Relative Fluid Overload (6, 7), we consider it to most accurately describe overhydration indexed to ECW.*

5 BASELINE CHARACTERISTICS

In order to assess balance of baseline characteristics between randomized arms of BCM substudy, the following variables recorded at Randomization (or at Screening) will be presented for each of the empagliflozin and placebo groups. All participants with at least one valid BCM measurement will be included, with missing baseline BCM values imputed using methods set out in section 7.1.

Note that these are a subset of the characteristics pre-specified in the main Data Analysis Plan (SOP11; EDMS#6290) plus other measures of anthropometry and BCM measurement variables. Categories will be consistent with those from the main trial publications or subgroup analyses:

a. History of prior disease:
    i. Diabetes mellitus (presence *vs* absence);
    ii. Self-reported heart failure (presence *vs* absence);
    iii. Primary renal diagnosis (diabetic kidney disease, hypertensive/renovascular disease, glomerular disease, other or unknown [1])
b. Patient characteristics;
    i. Age (continuous and categorised: <60; ⩾60 <70; ⩾70 years);
    ii. Sex (male *vs* female);
    iii. Race (White, Black/African American, South Asian, Southeast Asian, Mixed or Other);
    iv. Smoking status (ever smoked regularly at Randomization, yes *vs* no);
    v. Weight in kg*;
    vi. Body mass index (BMI) (continuous and categorised: <25; ≥25 <30; ≥30 kg/m^2);
    vii. Waist-to-hip ratio*;
    viii. Extracelllular water (ECW) in litres*;
    ix. Intracellular water (ICW) in litres*;
    x. Fluid Overload in litres*;
    xi. Relative Fluid Overload (%)*;
    xii. Clinically Significant Fluid Overload (%, presence *vs* absence)*;
        - Moderate

[1] Other includes tubulointerstitial disease, familial/hereditary nephropathies, other systemic disorders and miscellaneous renal disorders. Glomerular disease is subcategorised as follows: focal segmental glomerulosclerosis, IgA nephropathy, membranous nephropathy, minimal change disease and other glomerular disease.

- Severe (see section 4 for definitions)

xiii. Lean tissue index (LTI) (lean tissue mass [LTM] indexed to height) *;
xiv. Fat tissue index (FTI) (adipose tissue mass [ATM] indexed to height) *;
xv. Systolic blood pressure (continuous and categorised: <130; ≥130 <145; ≥145 mmHg);
xvi. Diastolic blood pressure (continuous and categorised: <75; ≥75 <85; ≥85 mmHg);

c. Laboratory values at Randomization:
   a. CKD-EPI estimated glomerular filtration rate (eGFR) (continuous and categorised: <30, ≥30 <45, ≥45 mL/min/1.73m^2 estimated from central enzymatic creatinine [or local creatinine where central value unavailable])
   b. Urinary albumin:creatinine ratio (ACR): (continuous and categorised: <30, ≥30 ≤300, >300 mg/g)
   c. Glycosylated haemoglobin (HbA1c) (continuous and categorised: <39 [normoglycaemia], ≥39<48 [pre-diabetes], ≥48<75 [well-controlled diabetes], ≥75 [poor glycaemic control] mmol/mol, or missing
   d. N-terminus prohormone of brain natriuretic peptide (NT-proBNP) (continuous and categorised: <110, ≥110 <330, ≥330 ng/L)
   e. Haematocrit (continuous and categorised: <37%; ≥37% <41%; ≥41%)

d. Medication use at randomization:
   i. RAS inhibition (yes *vs* no);
   ii. Diuretics (yes *vs* no, and analyses by type [loop *vs* thiazide *vs* mineralocorticoid receptor antagonist *vs* other potassium-sparing].
   iii. Antidiabetic medications (yes *vs* no, and analyses by type [biguanide *vs* sulphonylurea *vs* insulin *vs* DPP-4 inhibitor *vs* GLP-1 agonist *vs* other]

* continuous and categorized into approximate thirds of the distribution.

In general, baseline characteristics presented in publications will include all those listed above, with those provided in main versus subsidiary tables selected based upon relevance to the publication. For continuous variables, mean (standard deviation) will be presented unless the variable has a skewed distribution, in which case median (interquartile range) will be used. For all categorical variables, the number and percentage of participants in the category will be presented. All possible categories will be displayed, zero-filled where necessary, the category ‘missing’ will only be displayed (e.g. in footnotes) if there are actually missing values.

6 DEFINITIONS OF KEY RANDOMIZED ASSESSMENTS

BCM measurements were specified to be performed at Randomization, 2 and 18 months of Follow-up Visits (EDMS#6251). At these visits, weight, waist circumference, and hip circumference were measured together with blood and urine for central analysis and storage. The COVID-19 pandemic caused a substantial proportion of face-to-face Follow-up Visits to be delayed, however BCM measurements were permitted at later attended Follow-up Visit appointments, as outlined in the table below. Unless otherwise specified, all analyses will involve an intention-to-treat comparison among all randomized participants with at least one valid BCM measurement during Follow-up of the effects of allocation to empagliflozin versus placebo during the scheduled treatment period (i.e. all participants will be included irrespective of whether they take none, some or all of their allocated treatment) (8-10). Handling of missing valid BCM measurements is described in <u>section 7.1</u>.

Scheduled Follow-up Visits relative to the Randomization Visit date

Trial visit number	Follow-up month	Follow-up period	Ideal Follow-up day
1	2	≥30, <400 days	60 days
4	18	≥400 days, until Final Follow-up*	540 days

* Assume **<**680 days for maximum window for purposes of calculating weighting.

6.1 Hypotheses

For all statistical tests (other than tests for heterogeneity or trend), the null hypothesis will be that the effect of allocation to empagliflozin on the parameter of interest (e.g. Fluid Overload) in the target population is the same as the effect of allocation to placebo (and hence the alternative hypothesis will be that the effect of allocation to empagliflozin is not the same as the effect of allocation to placebo).

6.2 Primary randomized assessment

The primary assessment will be the effect of allocation to empagliflozin on mean absolute Fluid Overload in litres. Effects on Relative Fluid Overload (overhydration indexed to ECW, expressed as a percentage) will be presented alongside. Effects will be averaged over the two Follow-up time points (with weights proportional to the amount of time between visits, see <u>section 7.2.1</u>), adjusted for Randomization Fluid Overload values. The details of analysis methods for the primary assessment are described in <u>section 7.2.1</u>.

6.3 Key secondary randomized assessment

The key secondary composite outcome combines clinical outcome data with BCM measurements. Important data on fluid overload captured by BCM measurements is missed when remote Follow-up visits are necessary (e.g. as a result of the COVID-19 pandemic) or after death, so the composite outcome serves to capture all recorded data on fluid overload and its clinical consequences (whether measured by BCM or reflected in reported adverse events). The key secondary assessment is time-to-first development or worsening of Clinically Significant Fluid Overload. The composite outcome is defined as:

- Death from Heart Failure;
- Hospitalization for Heart Failure (as defined for the main trial analyses in SOP11; EDMS#6290); or
- Development of moderate Clinically Significant Fluid Overload (defined as >7% to ≤15% Relative Fluid Overload) among those without any Clinically Significant Fluid Overload at baseline; or
- Development of severe Clinically Significant Fluid Overload (defined as >15% Relative Fluid Overload) among those without this outcome at baseline.

The analysis method is described in section 7.2.2.

6.4 Other secondary randomized assessment

The other secondary assessment is to test whether the effects of empagliflozin 10mg versus matching placebo on Fluid Overload vary with time – in addition to the primary randomized assessment, analyses will be presented for the separate early (2-month) versus late (18-month) time points. The analysis method is described in section 7.2.3.

6.5 Tertiary randomized assessments including subgroup analyses

Tertiary assessments include:

i. Whether any effects of empagliflozin 10mg versus matching placebo are modified by baseline factors listed in section 5 for the primary assessment (absolute Fluid Overload). Subgroups based on sex, diabetes status, NT-proBNP, and eGFR will be the key subgroups and will be emphasised in presentation and interpretation. The sensitivity of subgroup assessments to indexing to ECW will be assessed by repeating subgroup analyses for the outcome of Relative Fluid Overload.

ii. The effects of empagliflozin 10mg versus matching placebo overall, and also early versus later during follow-up on:

a. Extracellular water (ECW)
b. Intracellular water (ICW)
c. Lean tissue index (LTI) (lean tissue mass [LTM] indexed to height)
d. Fat tissue index (FTI) (adipose tissue mass [ATM] indexed to height)
e. Body weight
f. BMI
g. Waist circumference
h. Hip circumference
i. Waist-to-hip ratio

iii. The effects of empagliflozin 10mg versus matching placebo on the four separate components of the key secondary outcome of development or worsening of Clinically Significant Fluid Overload.

iv. The effects of empagliflozin 10mg versus matching placebo on regression of Clinically Significant Fluid Overload from Severe (>15%) to Moderate (>7%); Severe to normal (≤7%); or Moderate to normal.

The analysis method for tertiary assessments is described in section 7.2.4.

6.6 Additional exploratory analyses

Additional exploratory analyses are planned however these are beyond the scope of this DAP and will be described in detail elsewhere.

7 STATISTICAL METHODOLOGY

7.1 Handling of missing and extreme values

Participants with a missing baseline BCM measurement will still be included in analyses if subsequent BCM measurements are obtained within the 2- and/or 18-month Follow-up windows. Missing baseline BCM measurements will be imputed with the average observed value (in both treatment groups combined). Sensitivity analyses will be performed limited to participants with complete baseline BCM data. Participants with missing baseline values relevant to subgroup analyses will be included in the subgroup containing the average value (or the most frequent category for a binary variable). Missing Follow-up BCM measurements including Fluid Overload at 2 and 18 months will be handled in the mixed model repeated measures (MMRM) approach (as outlined in section 7.2.1).

7.2 Methods of analysis

7.2.1 Primary randomized assessment

Absolute Fluid Overload in litres will be analysed as a continuous variable. Extreme outliers (defined as >2 standard deviations from the mean) will be reviewed prior to unblinding to assess data quality and plausibility (see Appendix section 8.1). These analyses will be completed before any randomized comparisons are conducted. Differences in Fluid Overload between treatment groups will be assessed using a mixed model repeated measures (MMRM) approach adjusted for the elements included in the minimization algorithm which determined treatment allocation (age, sex, prior diabetes, eGFR, and urinary ACR [but not region as the BCM substudy was only conducted in Europe]).

The primary assessment will focus on a weighted average of the values at the two Follow-up time points with weighting based on the relative size of each Follow-up window as set out in section 6. As the first Follow-up window (2-month Follow-up) is 370 days (days 30-400 post-Randomization) and the second window (18-month Follow-up) assumed to be 280 days (days 400-680 post-Randomization), this effectively weights information at the first Follow-up visits as 55% compared to 45% at the second. This is appropriate as we hypothesise that there will be a greater effect of empagliflozin versus placebo on Fluid Overload at 2 months versus 18 months as the effect of empagliflozin on Fluid Overload is expected to develop rapidly and diminish over time. Additionally, changes to other medication which can influence fluid balance may occur over time. Time will be included in the model as a categorical variable to avoid assuming a linear association between treatment allocation and Fluid Overload over time. The model will include fixed, categorical effects of treatment allocation, treatment-by-time interaction, and the prognostic variables used in the minimization algorithm (in the same

categories used in the minimization process) along with continuous effects of baseline (randomization) measurements and baseline-by-time interaction. The within-person error correlations will be assumed to be unstructured.

7.2.2 Assessment for key secondary randomized assessment

Time-to-first event analyses will use adjusted Cox regression. The general statistical methods and approaches to subgroup analyses are set out in the main Data Analysis Plan (SOP11; EDMS#6290). Follow-up for the clinical components of the composite outcome will be censored according to the main Data Analysis Plan. Follow-up for the BCM-derived components of the development or worsening of Fluid Overload outcomes (see section 4 for definitions) will be censored on the day after the last valid BCM measurement (but these individuals may remain at risk of clinical outcomes) or at death/withdrawal of consent.

7.2.3 Other secondary randomized assessment

The effect of treatment allocation on Fluid Overload separately at 2 and 18 months (see section 6.4) will be analysed using the same MMRM approach outlined in 7.2.1.

7.2.4 Tertiary randomized assessments including subgroup analyses

The same MMRM approach outlined in section 7.2.1 will be used for tertiary assessments (i) and (ii) as described in section 6.5. Tertiary assessment (i) is an analysis of the primary outcome by subgroup. Subgroup analysis will be performed by fitting relevant interaction terms for subgroups in the MMRM model with the aim of assessing whether the proportional effects in specific subgroups are statistically different from the overall effect. Interpretation will take into account the number of subgroups assessed as well as biological rationale. Tertiary assessment (ii) will use the same MMRM approach as for the primary assessment (section 7.2.1). Tertiary assessments (iii) and (iv) which analyse effects of treatment allocation on the components of the composite key secondary outcome and regression of Clinically Significant Fluid Overload will be analysed according to the same time-to-event approach outlined in section 7.2.2.

Further technical documentation to accompany this Data Analysis Plan may also be added as an appendix, if additional methodological details for the approaches described in section 7 are found to be required.

8 APPENDIX: DEFINITION OF VALID BCM MEASUREMENTS AND DATA HANDLING CONSIDERATIONS

8.1 Definition of a valid BCM measurement

To be included in analyses, an EMPA-KIDNEY participant must have at least one valid BCM measurement during Follow-up and been allocated to empagliflozin 10mg or matching placebo. To be included in analyses, each BCM measurement must have a corresponding weight measurement recorded at the same visit, from which BCM parameters can be derived according to the procedure set out in EDMS#7248.

Validity of BCM measurements will be assessed, prior to unblinding. Measurements with an absolute Fluid Overload value more negative than -5 litres will be excluded due to implausibility[1]. Measurements with a Q value[2] of <80 (site staff were trained to repeat BCM measurements if the Q value was <80; EDMS#6240) will be identified for visual inspection of the associated Cole-Cole plot[3] to assess data quality and determine inclusion in analyses. Two observers blind to treatment allocation will independently assess Cole-Cole plots by visual inspection, applying pre-specified criteria (outlined in section 8.4), with any differences resolved by consensus discussion.

Information on completeness of valid BCM data at each visit (i.e. number of participants with at least one valid BCM measurement at each visit, no valid BCM measurement but at least one invalid measure, or no BCM measurement) will be presented in the substudy CONSORT flow diagram. Statistical comparisons by treatment will be presented for the following parameters:

- The distribution of Q values for measurements included in the main comparison and sensitivity analyses
- The distribution of time-to-measurements from Randomization for each Follow-up window.

---

[1] In pilot work, Cole-Cole plots were reviewed for all measurements with absolute Fluid Overload values >2 standard deviations from the mean in a preliminary dataset to inform this cut-off. Values more negative than -5 litres were consistently associated with poor quality Cole-Cole plots. Conversely, outlying positive values were found to consistently have good quality Cole-Cole plots (and are considered plausible results).

[2] The Q score is an assessment of data quality generated by the BCM where 100 is a perfect Q value. In pilot work, a random subset of 50 measurements with a Q score ≥80 were selected for Cole-Cole plot review. Q scores above this threshold were confirmed to be a reliable indicator of good data quality in the cohort.

[3] The Cole-Cole plot generated by the BCM device fits a curve to the measured impedance data and defines the extracellular and intracellular resistances upon which all body composition data are based. Visual inspection of Cole-Cole plots identifies artefact within the impedance data.

8.2 Handling multiple BCM measurements

8.2.1 Multiple valid BCM measurements at the same visit

In all analyses, if more than one valid BCM measurement is available at a single Follow-up visit (i.e. date), the measurement with the highest Q value will be used and additional measurements ignored. In the situation where >1 valid measurements are obtained with an identical Q value, the first measurement will be used.

8.2.2 Multiple valid BCM measurements within a Follow-up window

In all analyses, if valid BCM measurements are made on more than one day within a Follow-up period, then the valid BCM measurement made on the day nearest the ideal follow-up day will be used and other BCM measurement excluded (see section 6 for Follow-up days). In the situation where >1 valid BCM measurements are obtained within the Follow-up window on dates which are equidistant from the ideal Follow-up date, a mean value will be calculated and used in analyses. This is considered a more scientifically robust approach in this unique situation due to the hypothesised interaction of time in the association between treatment allocation and Fluid Overload which means that selecting one or other equidistant measurement on the basis of Q values could introduce bias.

8.2.3 Multiple measurements at different visits on a single BCM card

Where data for two separate visits is recorded on a single BCM card, valid BCM results will be derived for the separate visits, wherever possible.

8.3 Data processing: BCM variables

The BCM provides measurement of:

- Extracellular water (ECW) resistance (denoted as R_e)
- Intracellular water (ICW) resistance (denoted as R_i)

BCM data are downloaded to study-specific laptops in a .pat file format and imported into a Microsoft Excel™ spreadsheet according to the procedure set out in EDMS#7248.

The following data are extracted from the analysis database to allow processing of the BCM data:

- Age, recorded in whole years at the time of each BCM measurement
- Weight, measured in kilograms, at the time of each BCM measurement
- Height, measured in centimetres, at Randomization
- Sex, recorded as male or female, at Randomization

along with Re and Ri reported by the BCM

Standard formulae will be applied to methodology described by Moissl and Chamney et al (11, 12) [1] to derive the following:

- Body mass index (BMI) in kg/m^2 using height and weight
- Extracellular water (ECW) in litres
- Intracellular water (ICW) in litres
- Total body water (TBW) in litres, by addition of ECW and ICW values
- Absolute Fluid Overload in litres
- Relative Fluid Overload (indexed to ECW), expressed as %
- Lean tissue index (LTI)
- Fat tissue index (FTI)

[1] Methods will use different coefficients to those available in published the current literature (coefficients which have been shared with permission).

8.4 Criteria for rejecting BCM measurements by Cole-Cole plot visual inspection

The two diagrams below provide a basic interpretation of the Cole-Cole plot:

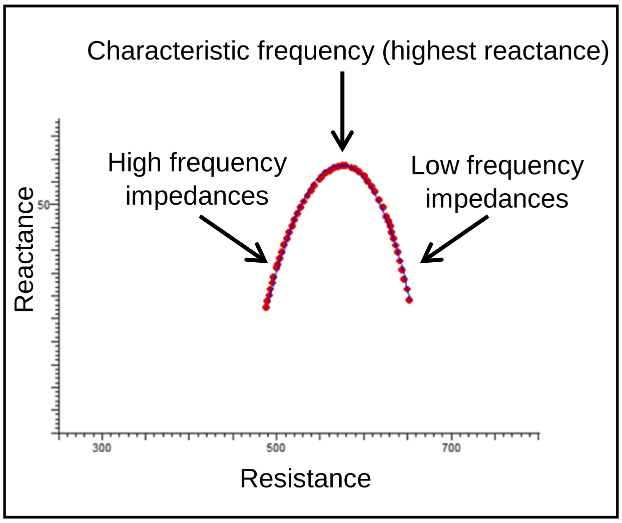

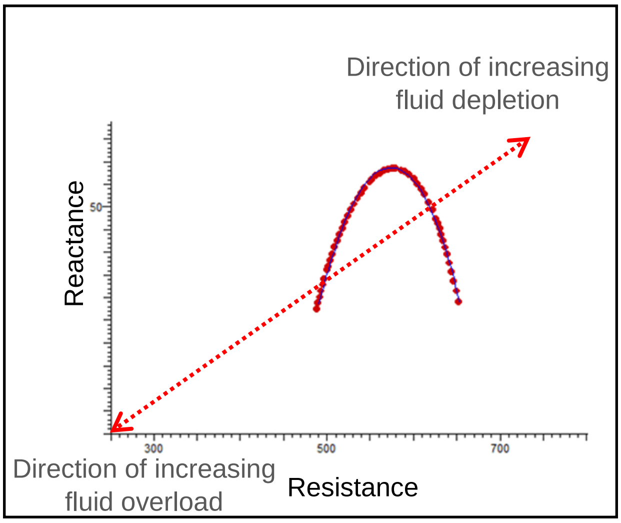

When manually reviewing Cole-Cole plots generated by the BCM for quality assurance, the following rule will be used to classify measurements as having poor data quality.

KEY CRITERION: In the opinion of the observer blind to treatment allocation, a good quality Cole-Cole plot should have the basic structure of a parabola, ignoring any artefacts at the high and low frequency end, and the plotted blue curve should closely fit the raw data red. Examples of good (“pass”) and poor (“fail”) quality bioimpedance data are provided below:

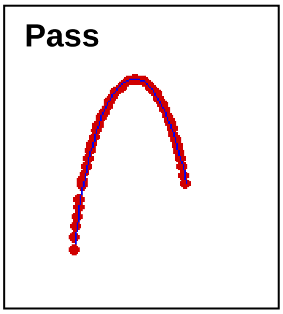

Parabola with good fit of plotted curve against raw data

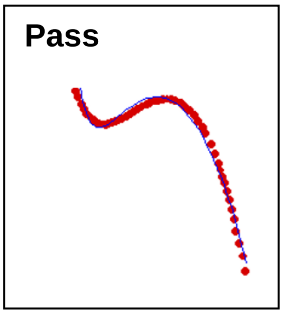

Ignoring artefact at high frequency, acceptable parabola with good fit

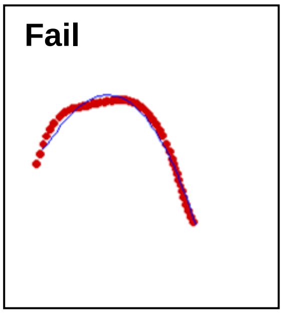

Raw data is not parabolic in shape & consequently poor fit

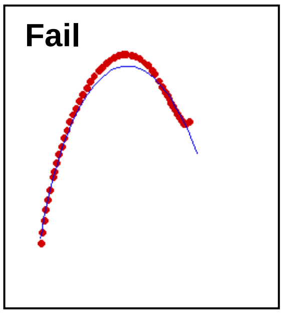

Unacceptable fit of plotted curve against raw data

Note: review of the Cole-Cole plot is not affected by the height or width of the plot, length of either end of a parabola, nor its position in the plot region.

8.5 Sensitivity analyses

Data quality assessment outlined in section 8.1 will be used to determine data inclusion in the primary analysis. Sensitivity analyses will also be conducted to assess the impact of the data quality assessments on the effects of empagliflozin versus placebo on the primary randomized assessment. These include analyses:

1. Of all single BCM measurements, irrespective of quality assessment or outlying values (i.e. the complete “unreviewed” set)
2. Restricted to single BCM measurements with a Q value ≥80 (i.e. a stricter criterion than the primary approach)

The criteria outlined in section 8.1 are thought to represent the optimal data quality assessment procedure to determine inclusion in the primary analysis and these sensitivity analyses represent the two alternative most extreme approaches.

REFERENCES

1. Wabel P, Moissl U, Chamney P, Jirka T, Machek P, Ponce P, et al. Towards improved cardiovascular management: the necessity of combining blood pressure and fluid overload. Nephrol Dial Transplant. 2008;23(9):2965-71.

2. Wizemann V, Wabel P, Chamney P, Zaluska W, Moissl U, Rode C, et al. The mortality risk of overhydration in haemodialysis patients. Nephrol Dial Transplant. 2009;24(5):1574-9.

3. Van Biesen W, Williams JD, Covic AC, Fan S, Claes K, Lichodziejewska-Niemierko M, et al. Fluid status in peritoneal dialysis patients: the European Body Composition Monitoring (EuroBCM) study cohort. PLoS One. 2011;6(2):e17148.

4. Zoccali C, Moissl U, Chazot C, Mallamaci F, Tripepi G, Arkossy O, et al. Chronic Fluid Overload and Mortality in ESRD. J Am Soc Nephrol. 2017;28(8):2491-7.

5. Dekker MJ, Marcelli D, Canaud BJ, Carioni P, Wang Y, Grassmann A, et al. Impact of fluid status and inflammation and their interaction on survival: a study in an international hemodialysis patient cohort. Kidney Int. 2017;91(5):1214-23.

6. Tabinor M, Elphick E, Dudson M, Kwok CS, Lambie M, Davies SJ. Bioimpedance-defined overhydration predicts survival in end stage kidney failure (ESKF): systematic review and subgroup meta-analysis. Sci Rep. 2018;8(1):4441.

7. O'Lone EL, Visser A, Finney H, Fan SL. Clinical significance of multi-frequency bioimpedance spectroscopy in peritoneal dialysis patients: independent predictor of patient survival. Nephrol Dial Transplant. 2014;29(7):1430-7.

8. Peto R, Peto J. Asymptotically Efficient Rank Invariant Test Procedures. 1972;135(2):185-207.

9. Peto R, Pike MC, Armitage P, Breslow NE, Cox DR, Howard SV, et al. Design and analysis of randomized clinical trials requiring prolonged observation of each patient. I. Introduction and design. Br J Cancer. 1976;34(6):585-612.

10. Peto R, Pike MC, Armitage P, Breslow NE, Cox DR, Howard SV, et al. Design and analysis of randomized clinical trials requiring prolonged observation of each patient. II. analysis and examples. Br J Cancer. 1977;35(1):1-39.

11. Moissl UM, Wabel P, Chamney PW, Bosaeus I, Levin NW, Bosy-Westphal A, et al. Body fluid volume determination via body composition spectroscopy in health and disease. Physiol Meas. 2006;27(9):921-33.

12. Chamney PW, Wabel P, Moissl UM, Müller MJ, Bosy-Westphal A, Korth O, et al. A whole-body model to distinguish excess fluid from the hydration of major body tissues. Am J Clin Nutr. 2007;85(1):80-9.

Supplemental Tables

Supplemental Table 1: Bioimpedance substudy cohort: additional baseline characteristics

	Empagliflozin (n=332)	Placebo (n=328)	Overall (n=660)
**DEMOGRAPHICS**			
**Age** (years)			
<60	103 (31.0)	107 (32.6)	210 (31.8)
≥60 <70	81 (24.4)	83 (25.3)	164 (24.8)
≥70	148 (44.6)	138 (42.1)	286 (43.3)
**CAUSE OF KIDNEY DISEASE**			
Diabetic kidney disease	70 (21.1)	52 (15.9)	122 (18.5)
Hypertension/renovascular	64 (19.3)	75 (22.9)	139 (21.1)
Glomerular	87 (26.2)	87 (26.5)	174 (26.4)
Other	47 (14.2)	61 (18.6)	108 (16.4)
Unknown	64 (19.3)	53 (16.2)	117 (17.7)
**HISTORY OF SMOKING**	178 (53.6)	161 (49.1)	122 (18.5)
**CLINICAL MEASUREMENTS**			
**Weight** (kg)			
<80	107 (32.2)	121 (36.9)	228 (34.5)
≥80 <95	113 (34.0)	100 (30.5)	213 (32.3)
≥95	112 (33.7)	106 (32.3)	218 (33.0)
Missing	0 (0.0)	1 (0.3)	1 (0.2)
**Waist-to-hip ratio**			
<0.9	39 (11.7)	40 (12.2)	79 (12.0)
≥0.9 <1.0	101 (30.4)	96 (29.3)	197 (29.8)
≥1.0	192 (57.8)	192 (58.5)	384 (58.2)
Missing	0 (0)	0 (0)	0 (0)
**Body mass index** (kg/m^2)			
<25	54 (16.3)	62 (18.9)	116 (17.6)
≥25 <30	133 (40.1)	133 (40.5)	266 (40.3)
≥30	145 (43.7)	132 (40.2)	277 (42.0)
Missing	0 (0)	1 (0.3)	1 (0.2)
**Systolic BP** (mmHg)			
<130	111 (33.4)	114 (34.8)	225 (34.1)
≥130 <145	112 (33.7)	106 (32.3)	218 (33.0)
≥145	109 (32.8)	108 (32.9)	217 (32.9)
Missing	0 (0)	0 (0)	0 (0)
**Diastolic BP** (mmHg)			
<75	140 (42.2)	126 (38.4)	266 (40.3)
≥75 <85	86 (25.9)	102 (31.1)	188 (28.5)
≥85	106 (31.9)	100 (30.5)	206 (31.2)
Missing	0 (0)	0 (0)	0 (0)
**BIOIMPEDANCE-DERIVED PARAMETERS***			
**Absolute "Fluid Overload"** (L)			
Mean (SD)	0.45 (1.68)	0.32 (1.68)	0.39 (1.68)
Distribution			
<-1	32 (9.6)	34 (10.4)	66 (10.0)
≥-1 <+1	136 (41.0)	155 (47.3)	291 (44.1)
≥+1	154 (46.4)	133 (40.5)	287 (43.5)
**Relative "Fluid Overload"** (%)			
Mean (SD)	1.9 (8.7)	1.3 (8.3)	1.6 (8.5)
Distribution			
<-3	75 (22.6)	90 (27.4)	165 (25.0)
≥-3 <+5	117 (35.2)	129 (39.3)	246 (37.3)
≥+5	130 (39.2)	103 (31.4)	233 (35.3)
**Extracellular water** (L)			
Mean (SD)	19.0 (3.8)	18.4 (3.7)	18.7 (3.8)
Distribution			
<16	61 (18.4)	72 (22.0)	133 (20.2)
≥16 <20	122 (36.7)	133 (40.5)	255 (38.6)
≥20	139 (41.9)	117 (35.7)	256 (38.8)
**Intracellular water** (L)			
Mean (SD)	20.7 (4.5)	20.1 (4.6)	20.4 (4.6)
Distribution			
<17	61 (18.4)	68 (20.7)	129 (19.5)
≥17 <22	135 (40.7)	138 (42.1)	273 (41.4)
≥22	126 (38.0)	116 (35.4)	242 (36.7)

Supplemental Table 1 (continued)

	Empagliflozin (n=332)	Placebo (n=328)	Overall (n=660)
**Lean tissue index** (kg/m^2)			
Mean (SD)	13.3 (3.1)	12.9 (3.0)	13.1 (3.1)
Distribution			
<11	57 (17.2)	69 (21.0)	126 (19.1)
≥11 <14	126 (38.0)	128 (39.0)	254 (38.5)
≥14	139 (41.9)	125 (38.1)	264 (40.0)
**Fat tissue index** (kg/m^2)			
Mean (SD)	12.6 (5.4)	12.5 (5.1)	12.5 (5.3)
Distribution			
<10	105 (31.6)	91 (27.7)	196 (29.7)
≥10 <14	92 (27.7)	123 (37.5)	215 (32.6)
≥14	125 (37.7)	108 (32.9)	233 (35.3)
**LABORATORY MEASUREMENTS**			
**Urinary albumin-to-creatinine ratio** (mg/g)			
<30	91 (27.4)	84 (25.6)	175 (26.5)
≥30 ≤300	90 (27.1)	101 (30.8)	191 (28.9)
>300	151 (45.5)	143 (43.6)	294 (44.5)
**Glycated hemoglobin** (mmol/mol)			
<39	129 (38.9)	141 (43.0)	270 (40.9)
≥39 <48	107 (32.2)	96 (29.3)	203 (30.8)
≥48 <75	86 (25.9)	78 (23.8)	164 (24.8)
≥75	4 (1.2)	6 (1.8)	10 (1.5)
Missing	6 (1.8)	7 (2.1)	13 (2.0)
**NT-proBNP** (ng/L)			
<110	100 (30.1)	90 (27.4)	190 (28.8)
≥110 <330	110 (33.1)	114 (34.8)	224 (33.9)
≥330	117 (35.2)	117 (35.7)	234 (35.5)
Missing	5 (1.5)	7 (2.1)	12 (1.8)
**Hematocrit** (%)			
Mean (SD)	39.2 (4.8)	39.3 (4.9)	39.3 (4.8)
Distribution			
<37	84 (25.3)	98 (29.9)	182 (27.6)
≥37 <41	107 (32.2)	89 (27.1)	196 (29.7)
≥41	124 (37.3)	131 (39.9)	255 (38.6)
Missing	17 (5.1)	10 (3.0)	27 (4.1)
**MEDICATIONS**			
Mineralocorticoid receptor antagonist	42 (12.7)	42 (12.8)	84 (12.7)
Loop diuretic	120 (36.1)	123 (37.5)	243 (36.8)
Thiazide diuretic	64 (19.3)	57 (17.4)	121 (18.3)
Potassium-sparing diuretic	5 (1.5)	0 (0)	5 (0.8)
Any diabetes therapy	119 (35.8)	102 (31.1)	221 (33.5)
Insulin	77 (23.2)	62 (18.9)	139 (21.1)
Sulfonylurea	19 (5.7)	14 (4.3)	33 (5.0)
Metformin	41 (12.3)	35 (10.7)	76 (11.5)
GLP-1 agonist	19 (5.7)	12 (3.7)	31 (4.7)
DPP-4 inhibitor	38 (11.4)	40 (12.2)	78 (11.8)
Other diabetes drug	4 (1.2)	1 (0.3)	5 (0.8)

Data are presented as mean (SD) or median (Q1-Q3) for continuous variables and n (%) for categorical variables. History of smoking = “ever smoked tobacco regularly” as determined by the participant. Weight, waist-to-hip ratio and all bioimpedance-derived parameters are presented as approximate tertiles, all other categorizations use pre-specified groupings as per the Data Analysis Plan. Bioimpedance-derived parameters were missing at baseline for 10 participants in the empagliflozin group (3.0%) and 6 participants in the placebo group (1.8%). *Bioimpedance measurements are presented for 644/660 participants with a baseline measurement (missing for 16/660) irrespective of validity for inclusion in the primary analysis. Abbreviations: NT-proBNP = N-terminal pro-brain-type natriuretic peptide; GLP-1 = glucagon-like peptide-1 receptor; DPP-4 = dipeptidyl-peptidase 4.

Supplemental Table 2: Bioimpedance substudy cohort: baseline characteristics by categories of baseline bioimpedance-derived “Fluid Overload”

	Fluid-deplete (n=89)	Normohydrated (n=399)	Moderate “Fluid Overload” (n=126)	Severe “Fluid Overload” (n=30)
	≤ -7%	> -7% ≤ +7%	> +7% ≤ +15%	> +15%*
**DEMOGRAPHICS**				
**Age** (years)	58.9 (14.6)	63.9 (14.9)	68.7 (12.8)	71.0 (9.1)
**Female sex**	47 (52.8)	115 (28.8)	37 (29.4)	3 (10.0)
**Race**				
Black/African American	0 (0.0)	3 (0.8)	0 (0.0)	0 (0.0)
Asian	4 (4.5)	7 (1.8)	1 (0.8)	0 (0.0)
Mixed/Other	0 (0.0)	6 (1.6)	3 (2.4)	0 (0.0)
White	85 (95.5)	383 (96.0)	122 (96.8)	30 (100.0)
**PRIOR DISEASE**				
**Diabetes**	26 (29.2)	143 (35.8)	58 (46.0)	21 (70.0)
**Heart failure**	10 (11.2)	78 (19.5)	34 (27.0)	13 (43.3)
**CLINICAL MEASUREMENTS**				
**Weight** (kg)	97.0 (22.9)	88.2 (18.9)	84.8 (20.1)	89.2 (15.6)
**BMI** (kg/m^2)	34.1 (7.2)	29.9 (5.9)	28.9 (6.0)	29.2 (4.6)
**Waist-to-hip ratio**	0.9 (0.1)	1.0 (0.1)	0.9 (0.1)	1.0 (0.2)
**Systolic BP** (mmHg)	132.4 (15.4)	137.5 (18.9)	137.0 (20.1)	148.8 (19.4)
**Diastolic BP** (mmHg)	80.3 (10.7)	79.2 (11.7)	74.4 (12.7)	76.4 (13.7)
**BIOIMPEDANCE-DERIVED PARAMETERS**				
**Absolute “Fluid Overload”** (L)	-2.1 (1.4)	0.1 (0.7)	2.1 (0.7)	4.1 (1.0)
**Relative “Fluid Overload”** (%)	-12.0 (7.2)	0.5 (3.8)	10.6 (2.4)	18.2 (2.9)
**Extracellular water** (L)	17.4 (3.6)	18.4 (3.5)	19.5 (4.1)	22.4 (3.2)
**Intracellular water** (L)	21.3 (5.9)	20.6 (4.4)	19.3 (4.3)	20.2 (2.9)
**Lean tissue index** (kg/m^2)	13.4 (4.3)	13.2 (2.9)	12.5 (2.6)	12.8 (2.2)
**Fat tissue index** (kg/m^2)	15.8 (6.7)	12.3 (5.0)	11.5 (4.4)	11.0 (3.7)
**LABORATORY MEASUREMENTS**				
**Estimated GFR** (mL/min/1.73m^2)				
Mean (SD)	39.5 (12.9)	36.0 (12.2)	34.5 (12.7)	33.0 (11.0)
Distribution				
<30	17 (19.1)	141 (35.3)	61 (48.4)	15 (50.0)
≥30 <45	48 (53.9)	192 (48.1)	43 (34.1)	11 (36.7)
≥45	24 (27.0)	66 (16.5)	22 (17.5)	4 (13.3)
**Urinary albumin-to-creatinine ratio** (mg/g)	231 (21-852)	207 (22-938)	166 (29-982)	311 (47-1466)
**Glycated hemoglobin (mmol/mol)**	43.3 (12.3)	43.3 (10.9)	44.5 (10.8)	46.7 (11.6)
**NT-proBNP** (ng/L)	100 (48-223)	187 (88-399)	549 (172-1250)	953 (463-2168)
**MEDICATIONS**				
**RAS inhibitor**	84 (94.4)	357 (89.5)	111 (88.1)	26 (86.7)
**Any diuretic therapy**	49 (55.1)	191 (47.9)	86 (68.3)	21 (70.0)

Data are not presented for 16/660 (2%) participants with missing bioimpedance data at baseline. *Categorization uses relative “Fluid Overload”; 7% and 15% approximately equate to absolute “Fluid Overload” thresholds of 1.1 and 2.5 L.

Supplemental Table 3: Baseline characteristics for the substudy, substudy region and the full trial cohorts

	**Bioimpedance Substudy Cohort (n=660)**	**Bioimpedance Substudy Countries** (i.e. all United Kingdom & Germany participants) **(n=2402)**	**Full Trial Cohort (N=6609)**
**DEMOGRAPHICS**			
**Age** (years)	64.6 (14.5)	65.5 (13.9)	63.8 (13.9)
**Female sex**	205 (31.1)	710 (29.6)	2192 (33.2)
**Race**			
Black/African American	3 (0.5)	74 (3.1)	262 (4.0)
Asian	12 (1.8)	38 (1.6)	2393 (36.2)
Mixed/Other	9 (1.4)	34 (1.4)	95 (1.4)
White	636 (96.4)	2256 (93.9)	3859 (58.4)
**PRIOR DISEASE**			
**Diabetes**	256 (38.8)	966 (40.2)	3040 (46.0)
**Heart failure**	136 (20.6)	380 (15.8)	658 (10.0)
**CLINICAL MEASUREMENTS**			
**Weight** (kg)	88.8 (19.8)	89.9 (19.9)	84.1 (21.4)
**Body mass index** (kg/m^2)	30.3 (6.2)	30.7 (6.3)	29.7 (6.8)
**Waist-to-hip ratio**	1.0 (0.1)	1.0 (0.1)	1.0 (0.1)
**Systolic BP** (mmHg)	137.3 (18.9)	137.0 (18.6)	136.5 (18.3)
**Diastolic BP** (mmHg)	78.2 (12.0)	78.2 (11.6)	78.1 (11.8)
**LABORATORY MEASUREMENTS**			
**Estimated GFR** ($mL/min/1.73m^2$)			
Mean (SD)	36.0 (12.4)	35.0 (12.0)	37.3 (14.4)
Distribution, n (%)			
<30	241 (36.5)	933 (38.8)	2282 (34.5)
≥30 <45	302 (45.8)	1098 (45.7)	2928 (44.3)
≥45	117 (17.7)	371 (15.4)	1399 (21.2)
**Urinary albumin-to-creatinine ratio** (mg/g)	203 (26-936)	220 (29-909)	329 (49-1069)
**HbA1c** (mmol/mol)	43.7 (11.1)	44.5 (12.7)	45.0 (13.6)
**NT-proBNP** (ng/L)	211 (93-581)	209 (92-571)	160 (69-419)
**MEDICATIONS**			
**RAS inhibitor**	592 (89.7)	2092 (87.1)	5628 (85.2)
**Any diuretic therapy**	353 (53.5)	1270 (52.9)	2815 (42.6)

Abbreviations: GFR = glomerular filtration rate; HbA1c = glycated haemoglobin; NT-proBNP = N-terminal pro-brain-type natriuretic peptide; RAS = renin-angiotensin system.

Supplemental Table 4: Sensitivity analyses for the effects of empagliflozin on mean bioimpedance-derived absolute "Fluid Overload" in L

			Empagliflozin		Placebo			
	Follow-up measurements analyzed	Participants analyzed	Mean	SE	Mean	SE	Absolute Difference	95% CI
**PRIMARY ASSESSMENT**	1047	620	0.10	0.05	0.34	0.05	-0.24	(-0.38, -0.11)
**Sensitivity analysis 1**								
Maximal inclusion: irrespective of quality assessment/implausible values	1082	629	-0.07	0.07	0.25	0.07	-0.32	(-0.52, -0.12)
**Sensitivity analysis 2**								
Limited to measurements with Q value ≥80	1029	614	0.07	0.06	0.37	0.06	-0.30	(-0.46, -0.14)
**Sensitivity analysis 3**								
Cohort with complete baseline bioimpedance data	1008	595	0.09	0.05	0.33	0.05	-0.24	(-0.38, -0.11)

All results are study averages (and absolute difference between treatment groups) for absolute "Fluid Overload" in L. Mean effects are adjusted for baseline absolute "Fluid Overload" (in continuous form) and for any differences in key baseline characteristics (categories of age, sex, diabetes, estimated glomerular filtration rate and urinary albumin-to-creatinine ratio) between treatment groups and weighted in proportion to the amount of follow-up time represented using an MMRM model (see Supplemental Methods). Out of a total of 1726 measurements from all participants: (a) 1650 measurements had Q scores ≥80 and 76 were <80; (b) 17 measurements had absolute "Fluid Overload" values more negative than -5 L (range -5.1, -13.0), of which 10 had a Q score <80, 7 had a Q score ≥80. All measurements with Q<80 and/or absolute "Fluid Overload" < -5 L were assessed by manual review of the Cole-Cole plot. 44 measurements (from 42 participants) were finally consider to be invalid (17 with extreme negative values and 27 identified based on Q score), the remaining 1682 measurements (for 660 participants) could be included in analyses. All analyses excluded the 40 consenting participants with no valid follow-up measurements (3 deaths before first follow-up measurement, 28 with no measurement performed and 9 excluded due to inadequate data quality). The median (Q1-Q3) Q value (quality score) for the empagliflozin vs placebo groups for each analysis were as follows, primary assessment: 94.0 (90.2-96.4) vs 94.5 (91.2-96.7), Wilcoxon rank sum p = 0.05; sensitivity analysis 1: 93.8 (89.7-96.3) vs 94.4 (90.7-96.7), p=0.05; sensitivity analysis 2: 94.1 (90.6-96.5) vs 94.6 (91.6-96.7), p=0.03; sensitivity analysis 3: 94.0 (90.2-96.4) vs 94.4 (91.1-96.7), p=0.09.

Supplemental Table 5: Bioimpedance substudy cohort: unadjusted baseline means and adjusted study averages for “Fluid Overload” for each subgroup by treatment group (additional data to accompany Figure 3)

Subgroup	Baseline mean (SE) "Fluid Overload" (L)		Adjusted study average (SE) "Fluid Overload" (L)		
	**Empagliflozin**	**Placebo**	**Empagliflozin**	**Placebo**	**Absolute difference (95% CI)**
**Sex**					
Male	0.67 (0.11)	0.60 (0.11)	0.17 (0.06)	0.41 (0.06)	-0.24 (-0.40, -0.08)
Female	0.12 (0.16)	-0.22 (0.11)	-0.05 (0.09)	0.21 (0.09)	-0.25 (-0.50, -0.00)
**Diabetes**					
Diabetes absent	0.14 (0.11)	0.21 (0.10)	0.10 (0.06)	0.29 (0.06)	-0.19 (-0.36, -0.02)
Diabetes present	1.02 (0.16)	0.61 (0.16)	0.13 (0.08)	0.44 (0.08)	-0.32 (-0.54, -0.10)
**NT-proBNP,** ng/L					
<110	-0.39 (0.14)	-0.27 (0.14)	0.03 (0.09)	0.38 (0.10)	-0.36 (-0.61, -0.10)
≥110 <330	0.40 (0.13)	0.06 (0.13)	0.15 (0.08)	0.23 (0.08)	-0.07 (-0.30, 0.15)
≥330	1.42 (0.16)	1.18 (0.15)	0.13 (0.09)	0.43 (0.08)	-0.30 (-0.53, -0.07)
**Estimated GFR,** mL/min/1.73m^2					
<30	0.94 (0.16)	0.51 (0.16)	0.22 (0.08)	0.33 (0.08)	-0.11 (-0.34, 0.12)
≥30 <45	0.17 (0.13)	0.26 (0.13)	0.06 (0.07)	0.37 (0.07)	-0.30 (-0.50, -0.11)
≥45	0.43 (0.22)	0.27 (0.19)	0.03 (0.11)	0.30 (0.12)	-0.27 (-0.59, 0.05)
**Overall**	**0.50 (0.09)**	**0.35 (0.09)**	**0.10 (0.05)**	**0.34 (0.05)**	**-0.24 (-0.38, -0.11)**

The adjusted effects of empagliflozin versus placebo on absolute "Fluid Overload" for each of these subgroups is plotted in Figure 3. Abbreviations: NT-proBNP = N-terminal pro-brain-type natriuretic peptide; GFR = glomerular filtration rate.

Supplemental Table 6: Effects of empagliflozin on tertiary (and *post-hoc*) bioimpedance-derived parameters assessments by time

	Empagliflozin (n=311)		Placebo (n=309)			
	Mean	SE	Mean	SE	Absolute Difference	95% CI
**EXTRACELLULAR WATER**, L						
Randomization	18.96	0.22	18.40	0.21		
2-month follow-up	18.19	0.07	18.70	0.07	-0.52	(-0.72, -0.32)
18-month follow-up	18.13	0.10	18.59	0.10	-0.46	(-0.74, -0.19)
**Study average**	**18.16**	**0.07**	**18.66**	**0.07**	**-0.49**	**(-0.69, -0.30)**
**INTRACELLULAR WATER**, L						
Randomization	20.63	0.27	20.12	0.25		
2-month follow-up	20.02	0.10	20.37	0.10	-0.35	(-0.64, -0.07)
18-month follow-up	20.20	0.14	20.44	0.13	-0.24	(-0.61, 0.14)
**Study average**	**20.10**	**0.10**	**20.40**	**0.10**	**-0.30**	**(-0.57, -0.03)**
**TOTAL BODY WATER**, L						
Randomization	39.59	0.46	38.51	0.44		
2-month follow-up	38.19	0.16	39.09	0.16	-0.89	(-1.33, -0.45)
18-month follow-up	38.32	0.21	39.04	0.21	-0.73	(-1.30, -0.15)
**Study average**	**38.25**	**0.15**	**39.07**	**0.15**	**-0.82**	**(-1.24, -0.40)**
**LEAN TISSUE INDEX**, kg/m^2						
Randomization	13.22	0.18	12.92	0.16		
2-month follow-up	12.81	0.09	13.00	0.09	-0.20	(-0.46, 0.06)
18-month follow-up	13.02	0.13	13.10	0.13	-0.08	(-0.43, 0.28)
**Study average**	**12.90**	**0.09**	**13.05**	**0.09**	**-0.14**	**(-0.39, 0.10)**
**FAT TISSUE INDEX**, kg/m^2						
Randomization	12.43	0.30	12.48	0.30		
2-month follow-up	12.44	0.10	12.50	0.10	-0.06	(-0.33, 0.22)
18-month follow-up	12.21	0.14	12.30	0.14	-0.10	(-0.49, 0.30)
**Study average**	**12.34**	**0.10**	**12.42**	**0.10**	**-0.07**	**(-0.35, 0.20)**

Mean effects are adjusted for baseline values of the dependent variable (in continuous form) and for any differences in key baseline characteristics (categories of age, sex, diabetes, estimated glomerular filtration rate and urinary albumin-to-creatinine ratio) between treatment groups and weighted in proportion to the amount of follow-up time represented using an MMRM model (see Supplemental Methods). Analysis excluded 40 consenting participants with no valid follow-up measurements (3 deaths before first follow-up measurement, 28 with no measurement performed and 9 excluded due to inadequate data quality). Analyses of effects on total body water were conducted as *post-hoc* exploratory analyses to aid interpretation of effects on "Fluid Overload"; total body water is the sum of extracellular and intracellular water.

Supplemental Table 7: Effects of empagliflozin on other bioimpedance-derived adiposity parameters

	Empagliflozin (n=311)		Placebo (n=309)			
	**Mean**	**SE**	**Mean**	**SE**	**Absolute Difference**	**95% CI**
**LEAN TISSUE MASS**, kg						
Randomization	39.40	0.66	38.24	0.61		
2-month follow-up	38.01	0.29	38.73	0.29	-0.72	(-1.52, 0.09)
18-month follow-up	38.65	0.39	39.05	0.38	-0.40	(-1.47, 0.66)
**Study average**	**38.28**	**0.27**	**38.87**	**0.27**	**-0.58**	**(-1.34, 0.18)**
**FAT TISSUE MASS**, kg						
Randomization	36.30	0.88	36.11	0.81		
2-month follow-up	36.19	0.30	36.38	0.30	-0.18	(-1.01, 0.64)
18-month follow-up	35.52	0.42	35.76	0.41	-0.24	(-1.39, 0.91)
**Study average**	**35.90**	**0.30**	**36.11**	**0.30**	**-0.21**	**(-1.04, 0.62)**
**ADIPOSE TISSUE MASS**, kg						
Randomization	49.39	1.19	49.13	1.10		
2-month follow-up	49.24	0.40	49.49	0.40	-0.25	(-1.37, 0.87)
18-month follow-up	48.33	0.57	48.66	0.56	-0.32	(-1.89, 1.24)
**Study average**	**48.85**	**0.41**	**49.13**	**0.41**	**-0.28**	**(-1.41, 0.85)**

The pre-specified analysis parameters lean tissue index and fat tissue index in kg/m^2 are calculated from lean tissue mass and adipose tissue mass in kg indexed to height squared. Adipose tissue mass consists of the fat tissue mass plus proteins, minerals and fluid. Mean effects are adjusted for baseline values of the dependent variable (in continuous form) and for any differences in key baseline characteristics (categories of age, sex, diabetes, estimated glomerular filtration rate and urinary albumin-to-creatinine ratio) between treatment groups and weighted in proportion to the amount of follow-up time represented using an MMRM model (see Supplemental Methods). Analysis excluded 40 consenting participants with no valid follow-up measurements (3 deaths before first follow-up measurement, 28 with no measurement performed and 9 excluded due to inadequate data quality).

Supplemental Table 8: Effects of empagliflozin on weight and body mass index (bioimpedance substudy & full trial cohorts)

	Empagliflozin		Placebo			
	Mean	SE	Mean	SE	Absolute Difference	95% CI
**WEIGHT**, kg						
**Bioimpedance substudy cohort**[*]						
Randomization	89.0	1.1	88.2	1.1		
2-month follow-up	87.8	0.2	88.7	0.2	-0.9	(-1.4, -0.3)
18-month follow-up	87.4	0.3	88.0	0.3	-0.6	(-1.5, 0.4)
**Study average**	**87.6**	**0.2**	**88.4**	**0.2**	**-0.7**	**(-1.3, -0.1)**
**Full trial cohort**[†]						
Randomization	84.0	0.4	83.9	0.4		
2-month follow-up	83.3	0.1	84.1	0.1	-0.7	(-1.0, -0.5)
18-month follow-up	82.4	0.1	83.3	0.1	-0.9	(-1.2, -0.5)
**Study average**	**82.3**	**0.1**	**83.2**	**0.1**	**-0.9**	**(-1.2, -0.6)**
**BODY MASS INDEX**, kg/m^2						
**Bioimpedance substudy cohort**[*]						
Randomization	30.2	0.3	30.1	0.4		
2-month follow-up	29.9	0.1	30.1	0.1	-0.3	(-0.5, -0.1)
18-month follow-up	29.7	0.1	29.9	0.1	-0.2	(-0.5, 0.1)
**Study average**	**29.8**	**0.1**	**30.0**	**0.1**	**-0.3**	**(-0.5, -0.1)**
**Full trial cohort**[†]						
Randomization	29.7	0.1	29.8	0.1		
2-month follow-up	29.5	0.0	29.8	0.0	-0.3	(-0.4, -0.2)
18-month follow-up	29.2	0.0	29.5	0.0	-0.3	(-0.4, -0.2)
**Study average**	**29.1**	**0.0**	**29.5**	**0.0**	**-0.3**	**(-0.4, -0.2)**

Mean effects are adjusted for baseline values of the dependent variable (in continuous form) and for any differences in key baseline characteristics (categories of age, sex, diabetes, estimated glomerular filtration rate, urinary albumin-to-creatinine ratio and, in the full trial cohort, region) between treatment groups and weighted in proportion to the amount of follow-up time represented using an MMRM model (see Supplemental Methods). Analyses include all individuals with at least one follow-up weight measurement. [*]Analyses in the bioimpedance substudy cohort use the 2 and 18 month time windows pre-specified in the substudy Data Analysis Plan and analyze the 620 individuals included in the MMRM analyses of bioimpedance parameters. [†]Analyses in the full trial cohort use all available measurements; effects by time at 6-, 12-, 24-, 30- and 36-month study visits are not shown. The effects of study treatment on weight were similar in substudy versus non-substudy participants (heterogeneity p value = 0.60) and there was no significant interaction between time and treatment effect on weight (p for interaction with time in full trial cohort = 0.47; substudy cohort = 0.44).

Supplemental Table 9: Effects of empagliflozin on waist and hip measurements (bioimpedance substudy & full trial cohorts)

	Empagliflozin		Placebo			
	Mean	SE	Mean	SE	Absolute Difference	95% CI
**WAIST CIRCUMFERENCE**, cm						
**Bioimpedance substudy cohort**						
Randomization	105.2	(0.8)	105.5	(0.8)		
**18-month follow-up**	106.1	(0.5)	105.5	(0.4)	0.6	(-0.7, 1.8)
**Full trial cohort**						
Randomization	102.8	(0.2)	102.7	(0.2)		
**18-month follow-up**	102.2	(0.2)	102.9	(0.2)	-0.8	(-1.2, -0.3)
**HIP CIRCUMFERENCE**, cm						
**Bioimpedance substudy cohort**						
Randomization	109.3	(0.7)	109.4	(0.7)		
**18-month follow-up**	109.6	(0.4)	109.1	(0.4)	0.4	(-0.6, 1.5)
**Full trial cohort**						
Randomization	107.3	(0.2)	107.2	(0.2)		
**18-month follow-up**	106.4	(0.2)	107.0	(0.2)	-0.6	(-1.1, -0.1)
**WAIST-TO-HIP RATIO**						
**Bioimpedance substudy cohort**						
Randomization	0.96	(0.01)	0.96	(0.01)		
**18-month follow-up**	0.97	(0.00)	0.97	(0.01)	0.00	(-0.02, 0.02)
**Full trial cohort**						
Randomization	0.96	(0.00)	0.96	(0.00)		
**18-month follow-up**	0.96	(0.00)	0.97	(0.00)	-0.00	(-0.01, 0.00)

Mean effects are adjusted for baseline values of the dependent variable (in continuous form) and for any differences in key baseline characteristics (categories of age, sex, diabetes, estimated glomerular filtration rate, urinary albumin-to-creatinine ratio and, in the full trial cohort, region). Waist, hip and the associated ratio measures were analyzed at a single follow-up time point and are therefore analyzed by analysis of covariance (ANCOVA). Full trial cohort analyses include all 6009 participants; missing measurements were handled by mean imputation for baseline and multiple imputation for follow-up measurements (see Supplemental Methods). Substudy cohort analyses include the 620 individuals included in the MMRM analyses of bioimpedance parameters, all of whom had a baseline waist-to-hip measurement; missing follow-up measurements were imputed following the same procedure for the full trial cohort.

Supplemental Table 10: Effects of empagliflozin on glycated hemoglobin and hematocrit (bioimpedance substudy & full trial cohorts)

		Empagliflozin		Placebo			
	**Participants analyzed**	**Mean**	**SE**	**Mean**	**SE**	**Absolute Difference (95% CI)**	
**GLYCATED HEMOGLOBIN**, mmol/mol							
**Bioimpedance substudy cohort**							
Prior diabetes	240	53.8	0.6	53.8	0.6	0.0	(-1.6, 1.6)
No prior diabetes	379	37.1	0.2	36.8	0.1	0.3	(-0.2, 0.7)
All	619	43.5	0.2	43.3	0.2	0.2	(-0.5, 0.9)
**Full trial cohort**							
Prior diabetes	2914	53.4	0.3	54.3	0.3	-0.9	(-1.6, -0.1)
No prior diabetes	3444	36.9	0.1	36.9	0.1	0.0	(-0.2, 0.2)
All	6358	44.5	0.1	44.9	0.1	-0.4	(-0.8, -0.0)
**HEMATOCRIT**, %							
**Bioimpedance substudy cohort**	196	41.7	0.3	39.1	0.3	2.5	(1.7, 3.4)
**Full trial cohort**	1368	40.7	0.1	38.4	0.1	2.3	(1.9, 2.7)

Study averages are adjusted for baseline values of the dependent variable (in continuous form) and for any differences in key baseline characteristics (categories of age, sex, diabetes, estimated glomerular filtration rate, urinary albumin-to-creatinine ratio and, in the full trial cohort, region) between treatment groups. Analyses of glycated hemoglobin use central laboratory samples from randomization, 2-, 18-, 24- and 30-month follow-up visits, weighted in proportion to the amount of follow-up time represented (see Supplemental Methods) in MMRM analyses. All participants with at least one follow-up measurement of glycated hemoglobin were included, full trial cohort analyses exclude those with missing baseline measurements. Hematocrit was only assessed in a ~20% subset of the full trial cohort using local laboratory measurements at randomization and 18 months using analysis of covariance (ANCOVA) and excludes those with missing baseline measurements. Hematocrit is analyzed in the 196 of the 620 bioimpedance substudy cohort with an 18-month measurement with mean imputation of missing baseline measurements for consistency with the substudy analysis approach.

Supplemental Table 11: Effects of empagliflozin on blood pressure (bioimpedance substudy & full trial cohorts)

	Empagliflozin		Placebo				
	**Mean**	**SE**	**Mean**	**SE**	**Absolute Difference**	**95% CI**	**P value for interaction with time**
**SYSTOLIC BLOOD PRESSURE**, mmHg							
**Bioimpedance substudy cohort***							0.029
Randomization	137.0	1.1	137.3	1.1			
2-month follow-up	132.0	0.9	136.3	0.9	-4.3	(-6.7, -1.9)	
18-month follow-up	132.2	1.1	134.2	1.1	-2.0	(-4.9, 0.9)	
**Study average**	**132.1**	**0.8**	**135.4**	**0.8**	**-3.3**	**(-5.5, -1.2)**	
**Full trial cohort†**							<0.001
Randomization	136.4	0.3	136.7	0.3			
2-month follow-up	131.8	0.3	135.8	0.3	-4.0	(-4.7, -3.2)	
18-month follow-up	133.0	0.3	135.3	0.3	-2.3	(-3.2, -1.4)	
**Study average**	**132.7**	**0.2**	**135.3**	**0.2**	**-2.6**	**(-3.3, -1.9)**	
**DIASTOLIC BLOOD PRESSURE**, mmHg							
**Bioimpedance substudy cohort***							0.14
Randomization	77.9	0.7	78.8	0.7			
2-month follow-up	77.3	0.5	77.8	0.5	-0.5	(-1.9, 0.9)	
18-month follow-up	77.7	0.6	77.5	0.6	0.2	(-1.5, 1.9)	
**Study average**	**77.5**	**0.4**	**77.7**	**0.4**	**-0.2**	**(-1.4, 1.0)**	
**Full trial cohort†**							0.004
Randomization	78.1	0.2	78.1	0.2			
2-month follow-up	76.3	0.2	77.4	0.2	-1.1	(-1.6, -0.7)	
18-month follow-up	76.6	0.2	77.0	0.2	-0.4	(-0.9, 0.1)	
**Study average**	**76.3**	**0.1**	**76.8**	**0.1**	**-0.5**	**(-0.9, -0.1)**	

Mean effects are adjusted for baseline values of the dependent variable (in continuous form) and for any differences in key baseline characteristics (categories of age, sex, diabetes, estimated glomerular filtration rate, urinary albumin-to-creatinine ratio and, in the full trial cohort, region) between treatment groups and weighted in proportion to the amount of follow-up time represented using an MMRM model (see Supplemental Methods). *Analyses in the bioimpedance substudy cohort use the 2 and 18 month time windows pre-specified in the substudy Data Analysis Plan and analyze the 620 individuals included in the MMRM analyses of bioimpedance parameters. †Analyses in the full trial cohort include all individuals with at least one follow-up measurement and use all available measurements; effects by time at 6, 12, 24, 30 and 36 month study visits are not shown. The p value for the interaction with time are extracted from likelihood ratio tests comparing models with and without an interaction term testing for significant interaction between treatment allocation and time (using all available time points). The effects of study treatment on systolic blood pressure were similar in substudy versus non-substudy participants (heterogeneity p value = 0.52).

Supplemental Table 12: Bioimpedance substudy cohort: effects of empagliflozin on dehydration by categories of baseline bioimpedance-derived “Fluid Overload”

	Empagliflozin		Placebo	
	**Number of participants/total**	**%**	**Number of participants/total**	**%**
**SERIOUS DEHYDRATION**				
Fluid-deplete	0/41	0.0	0/45	0.0
Normohydrated	1/207	0.5	2/211	0.9
Moderate “Fluid Overload”	3/70	4.3	2/56	3.6
Severe “Fluid Overload”	0/14	0.0	0/16	0.0
**Bioimpedance substudy overall**	**4/332**	**1.2**	**4/328**	**1.2**
**Full trial cohort**	**30/3304**	**0.9**	**24/3305**	**0.7**
**SYMPTOMATIC DEHYDRATION**				
Fluid-deplete	1/42	2.4	0/45	0.0
Normohydrated	3/207	1.4	3/211	1.4
Moderate “Fluid Overload”	6/70	8.6	2/56	3.6
Severe “Fluid Overload”	0/14	0.0	0/16	0.0
**Bioimpedance substudy overall**	**10/332**	**3.0**	**5/328**	**1.5**
**Full trial cohort**	**83/3304**	**2.5**	**76/3305**	**2.3**

Baseline “Fluid Overload” is categorised using relative “Fluid Overload”: fluid depletion = ≤ -7%, normohydration = > -7% ≤ +7%, moderate “Fluid Overload” = > +7% ≤ +15%, severe “Fluid Overload” = > +15%; participants without a valid baseline bioimpedance measurement are included in the normohydrated category based upon the imputed mean value. Symptomatic dehydration was defined as symptoms attributed by participants to dehydration, such as feeling faint or fainting. In the full trial cohort, there were 54 reports of serious dehydration (empagliflozin 30/3304 [%] vs placebo 24/3305 [%]; hazard ratio 1.25, 95% CI 0.73–2.14) and 159 reports of symptomatic dehydration (empagliflozin 83/3304 [%] vs placebo 76/3305 [%]; hazard ratio 1.10, 95% CI 0.81–1.51). In the full trial cohort, loop diuretic therapy was initiated during follow-up when not recorded at randomization in 159/2453 (6.5%) participants allocated empagliflozin versus 212/2409 (8.8%) allocated placebo; representing a 26% lower risk of requiring to start loop diuretics during follow-up among participants allocated empagliflozin versus placebo (RR 0.74, 95% CI 0.60-0.90).

Supplemental Figures

Supplemental Figure 1: Bioimpedance substudy cohort CONSORT flowchart

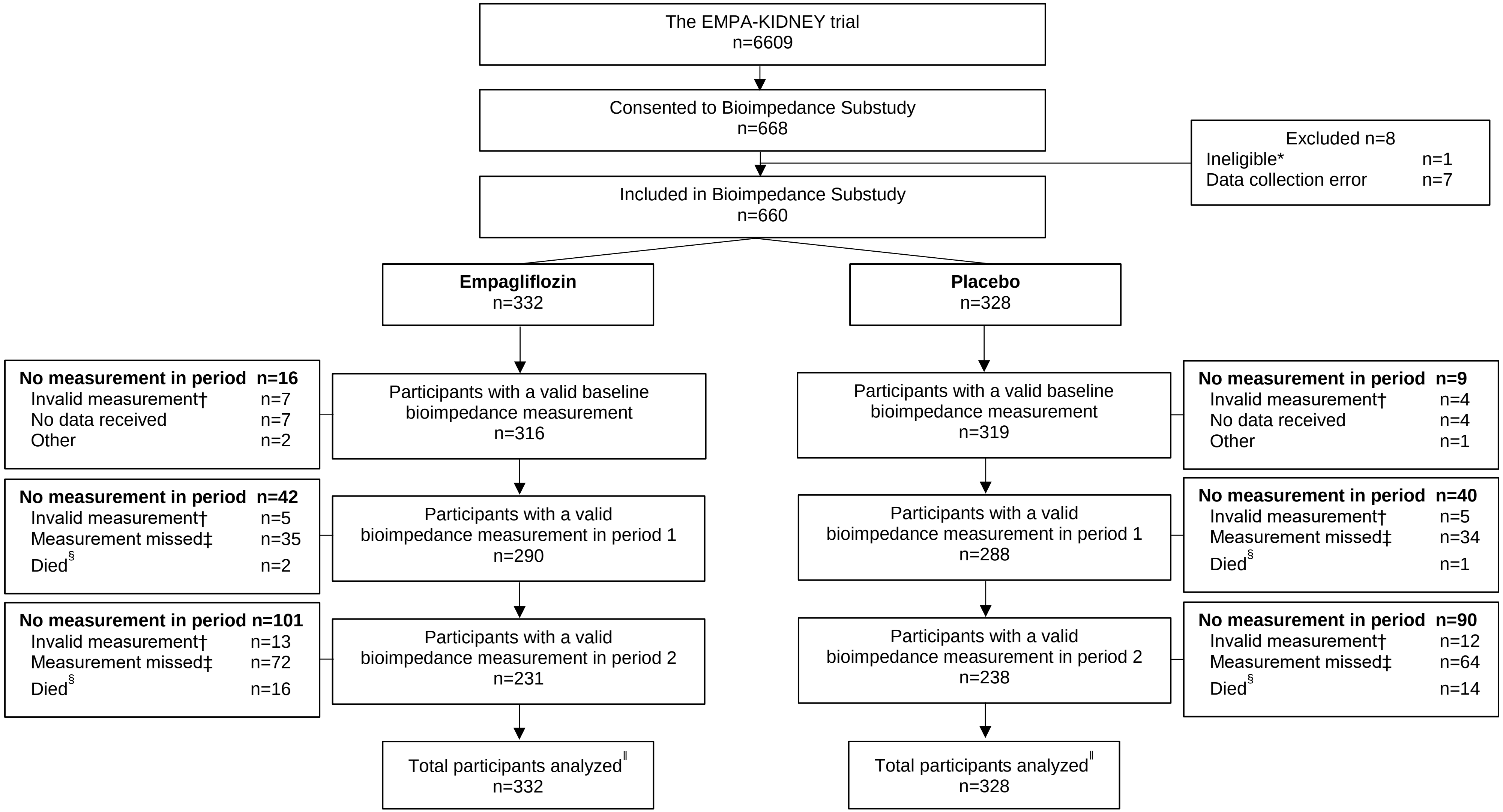

* Metal knee implants. † Invalid reasons: inadequate data quality, implausible outlying values or missing accompanying weight measurement (see Data Analysis Plan – Supplemental Material). ‡ Reasons for missed measurements were not recorded for all participants but include telephone follow-up (due to COVID-19), missed follow-up or rarely patient refusal/technical failure; missed measurement in one time period did not preclude a participant from future measurements. § Died within time window of missing bioimpedance measurement; does not include deaths after valid measurement obtained; total deaths until end of follow-up = 35 (empagliflozin=18; placebo=17). ‖ Number (proportion) of participants who reported taking "most" of their study treatment at 12 months: empagliflozin 282 (89%); placebo 292 (91%). All 660 participants were included in secondary analyses. The analysis population for MMRM analyses excluded 40 participants who did not have a single valid follow-up measurement.

Supplemental Figure 2: Effects of empagliflozin on mean absolute "Fluid Overload" (bioimpedance substudy cohort: post-hoc subgroups)

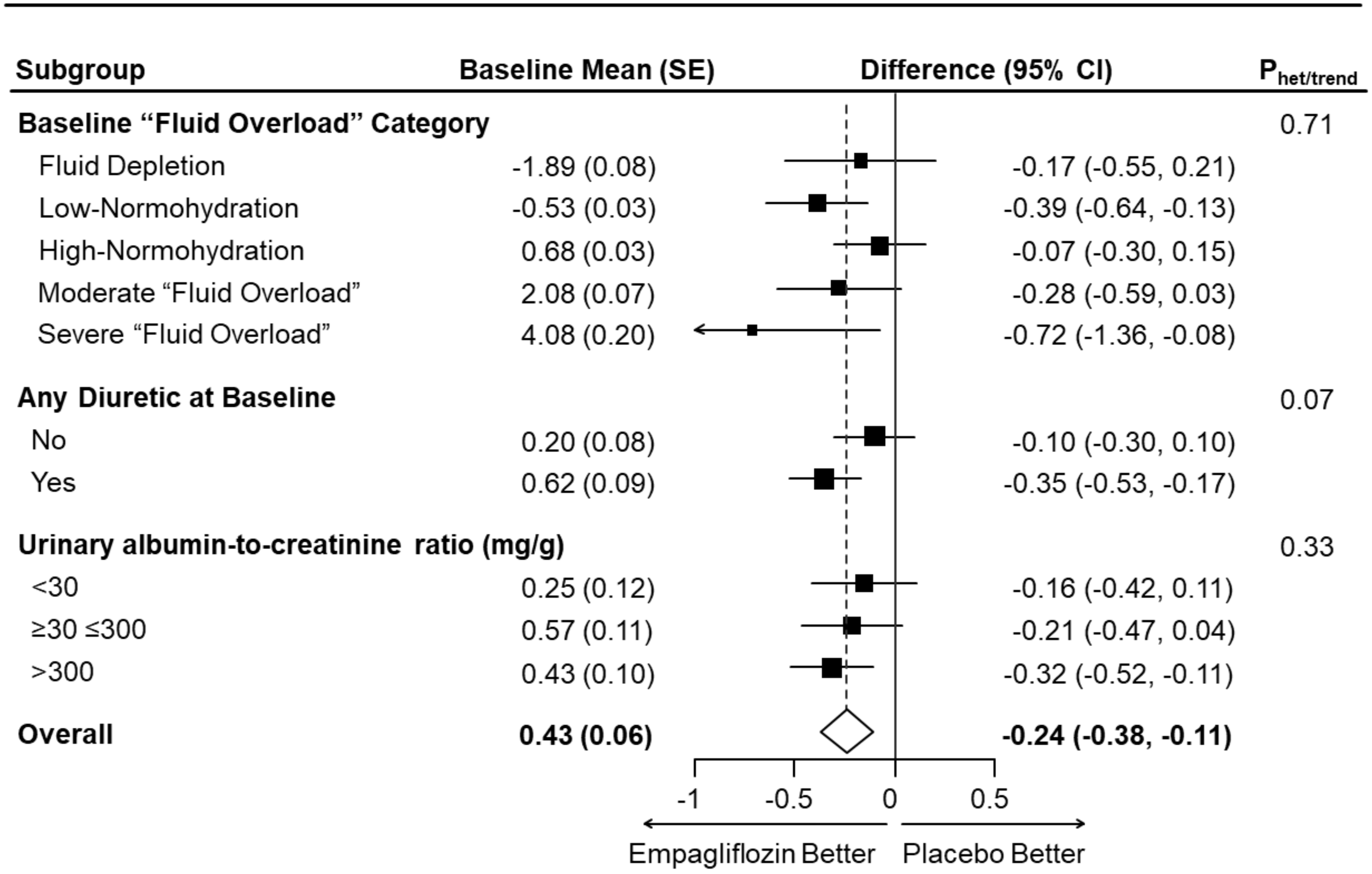

Baseline "Fluid Overload" subgroup analysis was conducted post-hoc and is categorised using relative "Fluid Overload": fluid depletion = ≤ -7%, low-normohydration = > -7% ≤ 0%, high-normohydration = >0% ≤ +7%, moderate "Fluid Overload" = > +7% ≤ +15%, severe "Fluid Overload" = > +15%; participants without a valid baseline bioimpedance measurement are included in the high-normohydration category based upon the imputed mean value. Mean effects are adjusted for baseline values of the dependent variable (in continuous form) and for any differences in key baseline characteristics (categories of age, sex, diabetes, estimated glomerular filtration rate, urinary albumin-to-creatinine ratio and, in the full trial cohort, region) between treatment groups and weighted in proportion to the amount of follow-up time represented using an MMRM model (see Supplemental Methods). Analysis excluded 40 consenting participants with no valid follow-up measurements (3 deaths before first follow-up measurement, 28 with no measurement performed and 9 excluded due to inadequate data quality).

Supplemental Figure 3: Correlation between change in weight (relative to baseline) with change in different bioimpedance indices at the 2-month follow-up visit, by treatment allocation

EMPAGLIFLOZIN **PLACEBO**

Total Body Water (TBW), L

2-month adjusted mean (SE): empagliflozin 38.2 (0.2); placebo 39.1 (0.2); between-group difference (95% CI) -0.9 (-1.3, -0.5) L

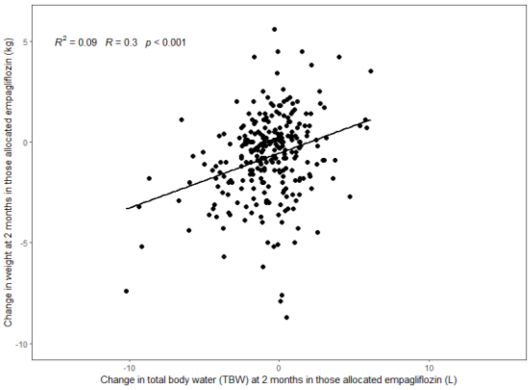

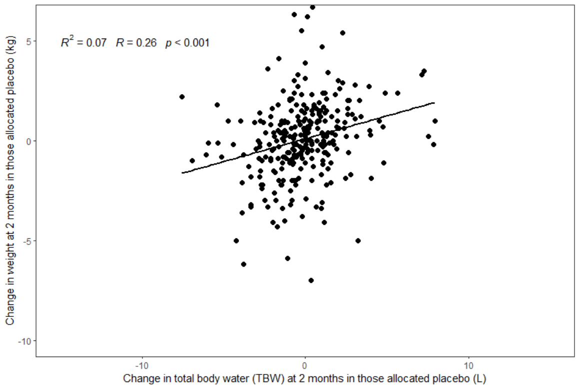

Adipose Tissue Mass (ATM), kg

2-month adjusted mean (SE): empagliflozin 49.2 (0.4); placebo 49.5 (0.4); between-group difference (95% CI) -0.3 (-1.4, 0.9) kg

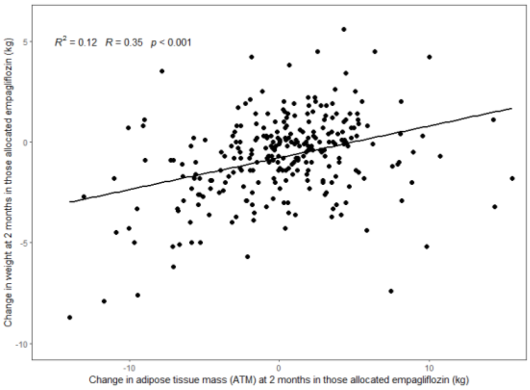

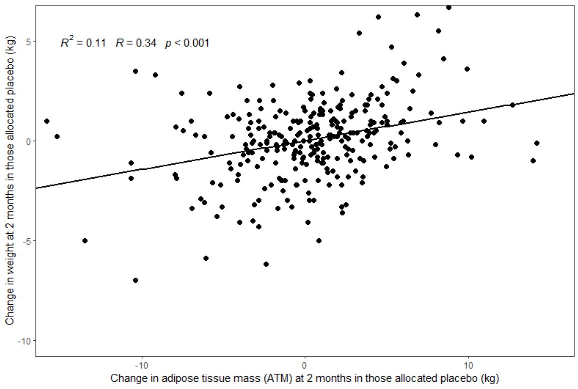

Lean Tissue Mass (LTM), kg

2-month adjusted mean (SE): empagliflozin 38.0 (0.3); placebo 38.7 (0.3); between-group difference (95% CI) -0.7 (-1.5, 0.1) kg

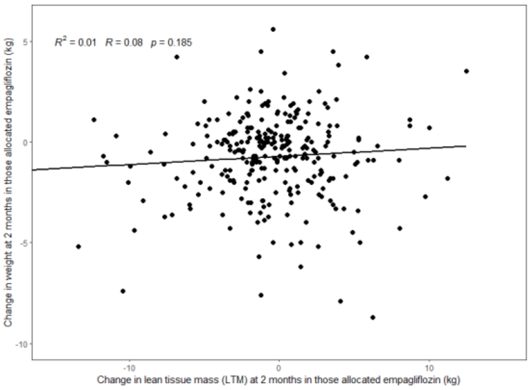

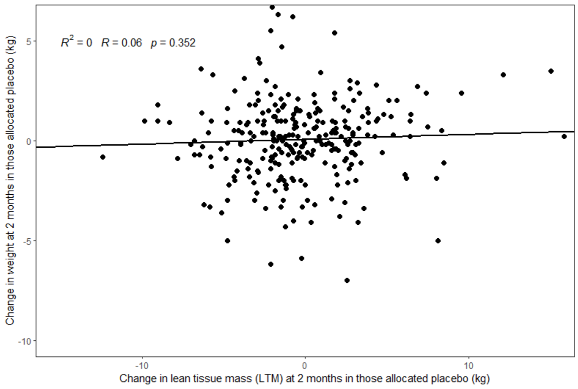

Correlations analysed post-hoc at peer reviewer request. Correlations shown are for substudy participants with valid and non-missing bioimpedance and weight measurements at the 2-month time point (empagliflozin n=273; placebo n=279). The correlations are non-randomized observational analyses and should not be attributed to effects of allocated treatment. Perfect correlation is not expected due to multiple determinants of weight (3 available bioimpedance indices are shown) and any measurement error in weight or bioimpedance indices. Randomized analyses assessing effects of allocated treatment (i.e. between-group difference with 95% CI) are provided for context with extracellular water and intracellular water combined to formulate total body water (in order to put effects on total fluid differences in the context of effects on body weight). At 2 months, the adjusted mean (SE) body weight in the substudy was: empagliflozin 87.8 (0.2); placebo 88.7 (0.2); between-group difference (95% CI) -0.9 (-1.4, -0.3) kg. Note that any measurement error between treatment groups would be expected to be non-differential with respect to treatment allocation due to the systematic use of the same methods of measurement and the trial's randomized double-blind design. The between group differences should therefore be considered to be reliable and unbiased with respect to treatment allocation. Any measurement error in weight or bioimpedance parameters would only result in lower precision (i.e. widened confidence intervals for the between-group differences) which increases the chances of a type 2 error (i.e. increases the chances of falsely concluding an absence of effect). A simulation-based example of classical measurement error in randomized trials is provided in Nab et al. Statistics in Medicine. 2019;38:5182–5196.

Supplemental Figure 4: Effects of empagliflozin on weight, body mass index, systolic blood pressure, glycated hemoglobin, and hematocrit by race (full trial cohort)

Race	Baseline Mean (SE)	Difference (95% CI)	p_{het}
**WEIGHT (kg)**			0.53
Asian	71.0 (0.3)	-1.1 (-1.6, -0.6)	
Black	98.5 (1.4)	-0.5 (-1.9, 1.0)	
Mixed/Other	87.5 (2.2)	-1.8 (-4.4, 0.7)	
White	91.0 (0.3)	-0.8 (-1.2, -0.4)	
**Overall**	**84.0 (0.3)**	**-0.9 (-1.2, -0.6)**	
**BODY MASS INDEX (kg/m²)**			0.38
Asian	26.4 (0.1)	-0.4 (-0.6, -0.2)	
Black	34.5 (0.5)	-0.2 (-0.7, 0.3)	
Mixed/Other	31.0 (0.7)	-0.7 (-1.6, 0.2)	
White	31.4 (0.1)	-0.3 (-0.4, -0.2)	
**Overall**	**29.7 (0.1)**	**-0.3 (-0.4, -0.2)**	
**SYSTOLIC BLOOD PRESSURE (mmHg)**			0.60
Asian	138.2 (0.4)	-2.6 (-3.9, -1.4)	
Black	135.9 (1.1)	-4.8 (-8.1, -1.6)	
Mixed/Other	138.0 (1.9)	-0.6 (-7.5, 6.3)	
White	135.5 (0.3)	-2.6 (-3.5, -1.7)	
**Overall**	**136.5 (0.2)**	**-2.6 (-3.3, -1.9)**	
**GLYCATED HEMOGLOBIN (mmol/mol)**			0.87
Asian	43.9 (0.3)	-0.5 (-1.1, 0.1)	
Black	48.5 (1.0)	1.4 (-0.6, 3.4)	
Mixed/Other	47.1 (1.4)	-2.8 (-6.2, 0.5)	
White	45.3 (0.2)	-0.3 (-0.8, 0.2)	
**Overall**	**44.9 (0.2)**	**-0.4 (-0.8, -0.0)**	
**HEMATOCRIT (%)**			0.90
Asian	39.0 (0.2)	2.6 (1.9, 3.2)	
Black	36.0 (0.9)	1.9 (-0.5, 4.3)	
Mixed/Other	38.7 (0.9)	1.0 (-2.1, 4.0)	
White	39.0 (0.2)	2.2 (1.7, 2.7)	
**Overall**	**38.9 (0.1)**	**2.3 (1.9, 2.7)**	

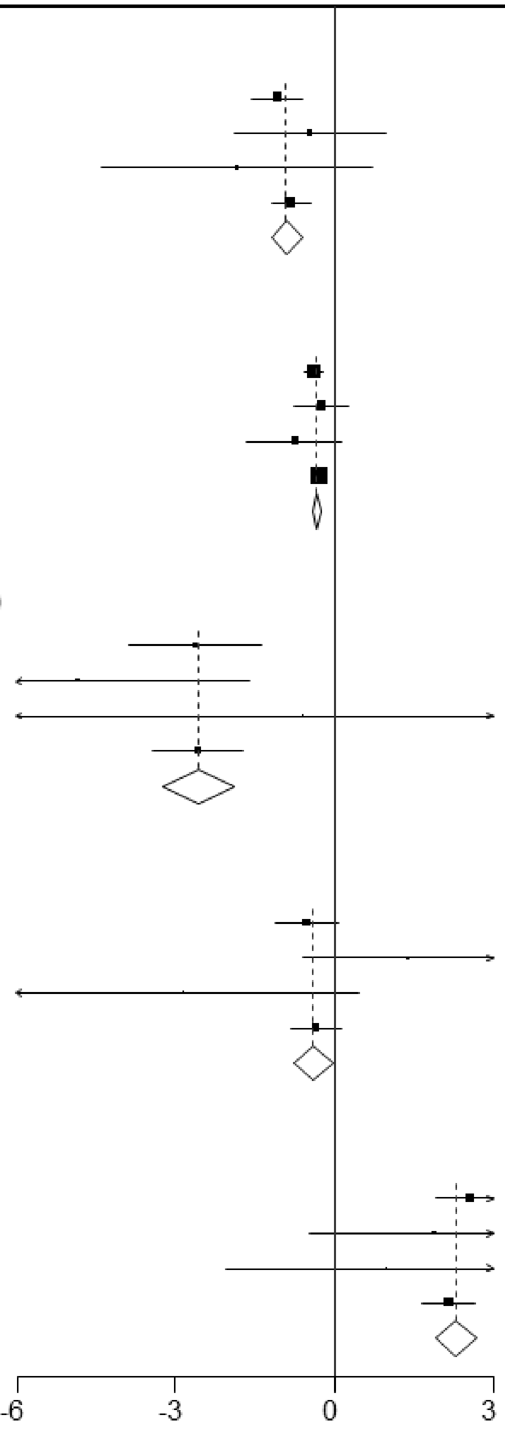

Overall results shown are the study averages using all available measurements for weight, body mass index and blood pressure; glycated hemoglobin and hematocrit analyses exclude measurements made outside of the specified time points in the Data Analysis Plan. Mean effects are adjusted for baseline values of the dependent variable (in continuous form) and for any differences in key baseline characteristics (categories of age, sex, diabetes, estimated glomerular filtration rate, urinary albumin-to-creatinine ratio and region) between treatment groups and weighted in proportion to the amount of follow-up time represented using an MMRM model (see Supplemental Methods). Analyses include all individuals with at least one measurement of the outcome variable during follow-up.
